# Molecular Solution to the Paradox of Ancient Brain Preservation

**DOI:** 10.1021/acs.jproteome.6c00200

**Published:** 2026-07-03

**Authors:** Alexandra Morton-Hayward, Sarah Flannery, Peter Berry, Iolanda Vendrell, Anders Johansen, Martin Hansen, Roman Fischer

**Affiliations:** † Department of Earth Sciences, 6396 University of Oxford, 3 South Parks Road, Oxford OX1 3AN, U.K.; ‡ Centre for Medicines Discovery, Nuffield Department of Medicine, University of Oxford, Roosevelt Drive, Oxford OX3 7FZ, U.K.; § Department of Environmental Science, Aarhus University, Frederiksborgvej 399, DK-4000 Roskilde, Denmark; ∥ Centre for Proteome Research, Institute of Systems, Molecular and Integrative Biology, 270024 University of Liverpool, Crown Street, Liverpool L69 7ZB, U.K.; ⊥ Department of Environmental Science and Resource Engineering, DTU Sustain, 5205 Technical University of Denmark, Bygningstorvet 115, DK-2800 Kongens Lyngby, Denmark

**Keywords:** taphonomy, bioarcheology, forensic proteomics, palaeoproteomics, neuropathology, neurodegeneration, brain aging, decay

## Abstract

Brains decompose rapidly after death yet frequently survive in the archeological record, including >1300 cases of waterlogged, oxygen-poor graves in which the brain is the only preserved soft tissue amongst otherwise skeletonized remains. To address this paradox, we decayed mouse carcasses for six months in four burial regimes varying in water and oxygen availability, characterized the brain proteome by high-resolution liquid chromatography–tandem mass spectrometry using parallel data-dependent and -independent acquisition workflows at six *post-mortem* intervals, and modeled >1.26 million peptide-specific decay trajectories to identify decay-prone and -resistant sequences. Oxygen availability exerted the main control on molecular fate: oxic burials produced widespread protein loss, whereas wet, hypoxic conditions favored retention of a distinctive subset of decay-resistant peptides. These surviving sequences were structurally ordered, enriched in redox-active residues and in regions that bind metals and lipids, and bore modification patterns consistent with radical-mediated oxidative cross-linking rather than fragmentation. By linking intrinsic tissue chemistry and environmental context, our results move brain preservation from anomaly to expectation: resolving why brains outlast other soft tissues in waterlogged, oxygen-poor burials, and revealing that the molecular signatures of *post-mortem* peptide persistence closely mirror those of pathological protein stabilization in brain aging and neurodegeneration.

## Graphical abstract

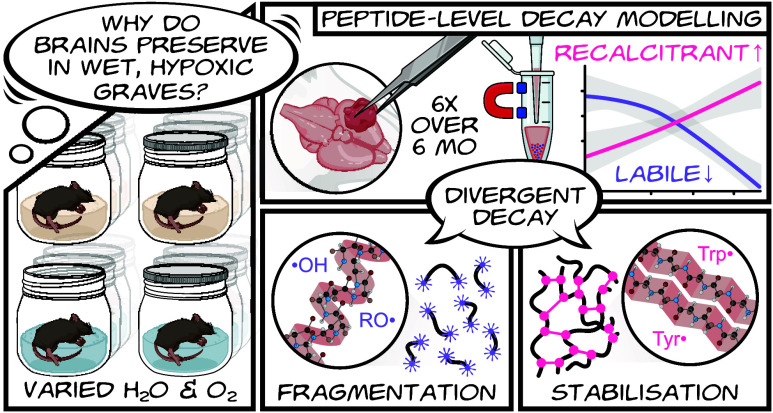

## Introduction

More than 4400 human brains have been recovered from burial sites spanning the last 12,000 years,^1^ and some of the oldest animal fossils preserve nervous tissue long after muscles and viscera have disappeared (e.g., refs 2–5 ); yet the brain is among the first organs to break down after death.^6^ This contradiction between the brain’s persistence over millennia and its biochemical lability *post-mortem* suggests that the central nervous system follows a taphonomic trajectory—a pathway by which biological tissues are altered, degraded, or preserved from the moment of death to their eventual recovery—that is both organ-specific and mechanistically distinct from those stabilizing other soft tissues.

In many cases, brain preservation fits familiar taphonomic trajectories. Freezing, dehydration, tanning, and saponification can stabilize multiple organs simultaneously, and preserved brains are often recovered alongside other soft tissues under these regimes.^1^ However, nearly one-third of archeological human brains (>1300) belong to an “unknown” preservation type: a resilient, shrunken mass that occurs as the sole surviving soft tissue within otherwise skeletonized remains, most commonly in waterlogged, low-oxygen environments.^1^ On the basis of their burial context and molecular composition, it has been proposed that these brains follow an organ-specific taphonomic trajectory governed by extensive molecular cross-linking,^7,8^ catalyzed by metals such as iron and copper,^1^ conceptually consistent with iron-mediated oxidative^9–11^ and glycoxidative/lipoxidative^12^ pathways implicated in deep-time fossilization of other soft tissues.^13^ This hypothesis remains conjectural, given that we lack a time-resolved picture of how the brain decomposes in different burial environments.

Although archeological brain preservation unfolds over centuries to millennia, the taphonomic trajectory that enables such survival is expected to be established much earlier. The early *post-mortem* interval (PMI)—on the order of days to months—is critical for determining long-term molecular fate^14^ because it is during this period that a range of biogeochemical factors^15^ interact either to permit continued degradation, or to divert tissues onto stabilizing pathways.^16–19^ Once labile components are lost and diffusion-limited chemistries become established, subsequent molecular change is expected to proceed slowly and conservatively.^14^ Thus, identifying the molecular mechanisms that operate during early decay is essential for understanding why certain tissues persist on archeological and, occasionally, paleontological time scales.

The existence of organ-specific taphonomic trajectories^20–24^ also bears on how *post-mortem* molecular profiles are interpreted more broadly. Preserved brains constitute the richest known reservoirs of ancient proteins,^25^ and *post-mortem* brain tissue is routinely examined in forensic^26^ and neuropathological contexts^27^; yet, across these settings, late-stage proteomic profiles are increasingly treated as informative rather than incidental. It is often assumed that, after the initial decomposition phase of autolysis (i.e., digestion of a tissue by its own enzymes), recovered proteins and their associated modifications primarily reflect *ante-mortem* biology rather than *post-mortem* chemistry.^28^ The prevalence of selectively preserved brains in waterlogged, oxygen-poor environments challenges this assumption, suggesting instead that burial conditions can actively redirect molecular fate and thereby shape the composition of the surviving proteome.

To test this possibility, we tracked time-resolved changes in the brain proteome of mice following burial under environmental conditions differing in water and oxygen availability (Figure 1). Using high-resolution liquid chromatography–tandem mass spectrometry (LC–MS/MS) with parallel data-dependent and -independent acquisition (DDA and DIA) workflows, we quantified peptide and protein abundance across a six-month PMI, and modeled >1.26 million peptide-specific decay profiles. We show that brain preservation in waterlogged, low-oxygen settings reflects the selective survival of peptides with shared sequence, structural, domain, and modification features, rather than uniform retention of the proteome. Identifying these features provides a basis for predicting which regions and types of proteins are likely to persist after death in different burial environments, and highlights mechanistic parallels with peptide architectures associated with pathological protein stabilization in life.

**Figure 1:**
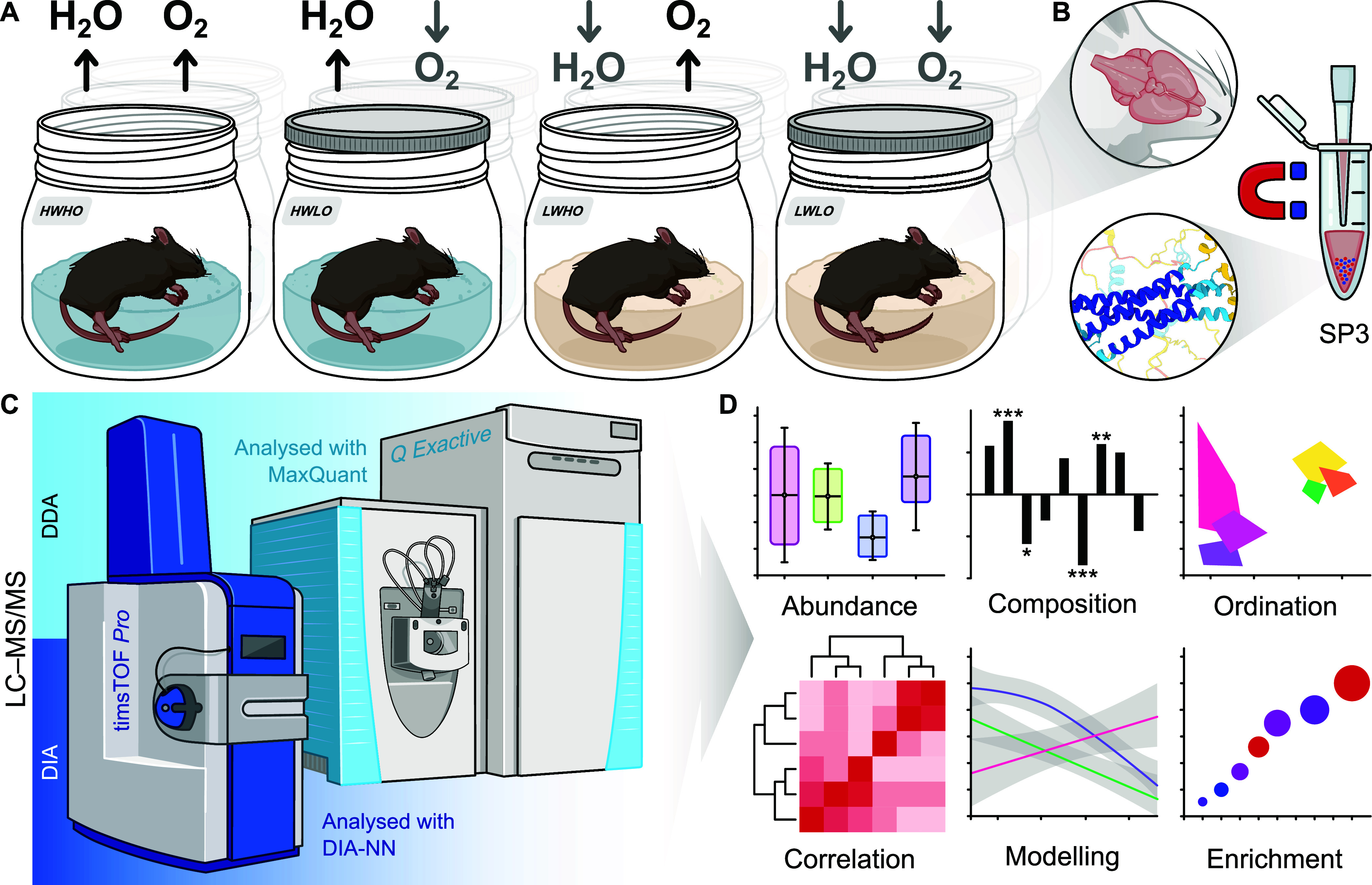
Overview of experimental and analytical workflow. (A) Experimental design. Whole mouse carcasses (*n* = 72) were buried individually in sterilized glass jars filled with sterilized quartz sand and ultrapure water under four burial regimes differing in water and oxygen availability: wet–oxic (HWHO), wet–hypoxic (HWLO), dry–oxic (LWHO), and dry–hypoxic (LWLO). Upward arrows indicate greater water and/or oxygen availability, and downward arrows indicate restricted availability. Carcasses were sampled at six time points (24 h, 72 h, 1 week, 6 weeks, three months, and six months), with three biological replicates per condition–time combination. (B) Brains were dissected, briefly homogenized, and proteins extracted using SP3-based digestion. (3D protein structure illustrated with AlphaFold).^29^ (C) Parallel liquid chromatography–tandem mass spectrometry (LC–MS/MS) acquisition strategies were employed^30^: data-dependent acquisition (DDA; *n* = 72) on a Q Exactive Plus Hybrid Quadrupole-Orbitrap mass spectrometer (Thermo Fisher Scientific) to maximize peptide and post-translational modification identification, and data-independent acquisition (DIA; *n* = 72) on a TimsTOF Pro mass spectrometer (Bruker) for quantitative proteome-wide profiling. DDA data were analyzed with MaxQuant (v.2.4.3.0)^31^ and DIA data with DIA-NN (v.2.0).^32^ (D) Downstream analytical framework applied to identify biochemical, compositional, structural, functional, and post-translational correlates of peptide and protein preservation, including multivariate ordination of scaled protein abundances, correlation and clustering analyses, modeling of peptide-specific decay profiles across burial conditions and PMIs, and differential abundance and functional enrichment analyses.

## Methods

### Experimental Taphonomy

A comprehensive description of all experimental and analytical procedures is provided in the Supporting Methods. All experimental procedures complied with the Danish Animal Experimentation Inspectorate (2020–15–0201–00432), and ethical approval was obtained from the Central University Research Ethics Committee at the University of Oxford (R80617/RE001). In brief, male, specific pathogen-free mice (*n* = 72; C57BL/6JRj, Janvier Laboratories) were reared and sacrificed under identical conditions for the purposes of other scientific research,^33^ and were donated to our study. Immediately after death, whole carcasses were flash-frozen in liquid N_2_ and stored at −80 °C. Immediately prior to the decay experiment, carcasses were thawed to room temperature. All equipment underwent dry heat sterilization at 160 °C for 12 h prior to use. Carcasses were buried individually in 500 mL glass Kilner jars containing 500 g of sterile quartz sand (210–297 μm granularity; Sigma–Aldrich), and four burial environments were established by factorial variation of waterlogging and oxygenation: wet–oxic (HWHO), wet–hypoxic (HWLO), dry–oxic (LWHO), and dry–hypoxic (LWLO). Wet and dry conditions were emulated by adding 180 and 90 mL ultrapure Milli-Q H_2_O (Sigma–Aldrich), respectively; oxic and hypoxic conditions (i.e., with and without atmospheric exchange and oxygen replenishment) were emulated by leaving jars open, or sealing them with an airtight silicon gasket. In wet conditions, water loss was periodically assessed by mass and corrected by replacement to maintain the target moisture. All jars were placed together in a fume hood with controlled temperature (20 °C), ambient laboratory lighting, and air circulation. Three biological replicates were decayed per condition at each of six time points (24 h, 72 h, 1 week, 6 weeks, three months, and six months; *n* = 12 mice per time point). At each time point, brains were dissected, homogenized, subdivided into five aliquots of approximately 80 mg, flash-frozen in liquid N_2_, and stored at −80 °C prior to proteomic analysis.

### Proteomics

For DDA (*n* = 72), tissue was lysed in 100 mM ammonium bicarbonate with 2% SDS (both Sigma–Aldrich), homogenized using a Precellys 24 Touch homogenizer (Bertin Technologies), and protein concentration estimated by bicinchoninic assay (Supporting Information, Table S24). For DIA (*n* = 72), an analogous workflow was used with Pierce RIPA lysis (Thermo Fisher Scientific) and adjusted protein input (Table S25). Proteins were reduced, alkylated, and processed by SP3 bead-assisted tryptic digestion^34^ prior to analysis by LC–MS/MS.

### Data Analysis

DDA and DIA raw files (*n* = 144) were searched against a curated*Mus musculus*brain reference proteome (*n* = 6252 proteins)^35^ using semispecific tryptic digestion, the fixed modification of carbamidomethylation of Cys, and the following variable PTMs: oxidation of Met and Pro; deamidation of Arg, Gln, and Asn; and protein N-terminal acetylation. DDA data were searched in MaxQuant (v.2.4.3.0)^31^ using label-free quantification (LFQ) with match between runs and dependent peptide search. DIA data were searched in DIA-NN (v.2.0)^32^ using a library-free workflow and processed in *DIAgui* (v.1.4.2)^36^ to perform MaxLFQ^37^ and missing-value imputation by quantile regression of left-censored data.^38,39^ To reduce the dependence of raw LFQ intensities on protein size, protein-level analyses were conducted on log_2_-transformed intensity-based absolute quantification (iBAQ) values, whereas peptide-level analyses were performed on the peptide intensities.

Global patterns in proteome composition were explored using principal component analysis (PCA) and uniform manifold approximation and projection (UMAP) of scaled log_2_ iBAQ values, revealing clear separation between “early” (<6 weeks) and “late” (≥6 weeks) PMIs. Differential protein abundance between early and late PMI was assessed on a per-condition basis with empirical-Bayes-moderated linear modeling, which stabilizes variance estimates across proteins and improves statistical power in high-dimensional proteomic data sets with limited replicate numbers. Significantly differentially abundant proteins were then grouped by *k*-means clustering according to whether they decreased or became relatively enriched throughout the PMI; these groups were then subjected to Gene Ontology (GO) enrichment analysis to identify the biological processes, molecular functions, and cellular components associated with each. Peptide preservation was quantified by modeling log_2_-intensity as a function of PMI to obtain peptide-specific decay slopes, and data-driven thresholds on these slopes were used to classify peptides as labile (decay-prone), putative (intermediate), and recalcitrant (decay-resistant). Biochemical, compositional, structural, functional, and post-translational correlates of preservation were then investigated by comparing known biochemical determinants of protein degradation rates^40^—including hydropathy, net charge, isoelectric point, sequence coverage, peptide length, amino acid composition, predicted secondary structure, intrinsic disorder, functional features, and modification frequencies—between preservation classes using appropriate regression and enrichment frameworks with multiple-testing correction.

## Results

A comprehensive description of all findings is provided in the Supporting Results.

### Divergent Taphonomic Trajectories under Oxic and Hypoxic Burial

To quantify how burial environment shapes decomposition of the brain proteome, we measured total protein concentration and protein identifications through time across four environments varying in water and oxygen availability, and summarized compositional change with multivariate ordination. The mean number of identified proteins decreased as decomposition progressed (Supporting Data S1 and Table S1), although a small, nonsignificant increase in mean protein concentration was observed at 72 h relative to 24 h, potentially reflective of early autolytic accumulation effects (Figure S1). Protein recovery was strongly environment-dependent, with the lowest mean recoveries in wet–oxic samples and the highest in dry–hypoxic samples (Figure S2a). Although most unique proteins (i.e., distinct protein groups; 69.6%) were identified at every time point (Figure S3b), and almost all unique proteins were identified in every condition (93.8%; Figure S2b), significant differences emerged between early (<6 weeks) and late (≥6 weeks) PMIs, and in comparisons involving wet–oxic burial (Tables S2 and S3). Temporal profiles indicated an apparent “pseudo-enrichment” of proteins under hypoxia (Figure 2a), with mean identifications at six months higher than at three months, whereas oxic conditions evidenced continued decline, including an order-of-magnitude reduction in wet–oxic samples by six months.

**Figure 2:**
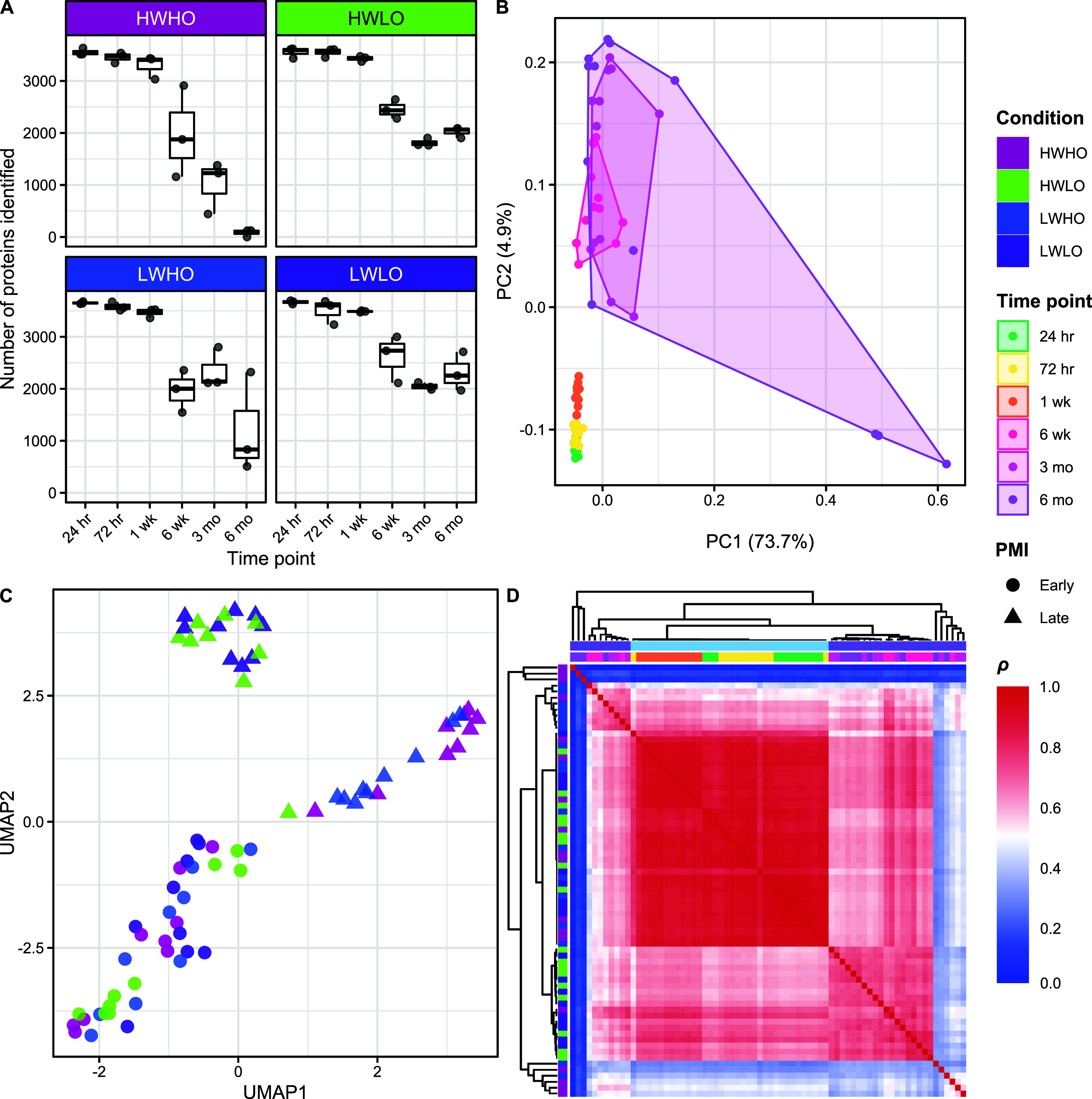
Divergent taphonomic trajectories under oxic and hypoxic burial. (A) Jittered box plots of the number of proteins identified at 1% false discovery rate (FDR) and *q*-value ≤ 0.01 recovered at each time point, in each burial condition. (B) PCA plot of normalized, batch effect-corrected abundance data (standardized and mean-centered) for all samples (*n* = 72; Supporting Methods). Each point represents the principal component (PC) score of a unique protein (*n* = 3910), with colors reflecting the time point at which that protein was in its highest mean condition. Together, PCs 1 and 2 explain 78.6% of the variation in the data set. (C) UMAP plot of normalized, batch effect-corrected abundance data (standardized and scaled) for all samples (*n* = 72; Supporting Methods). Each point represents a sample, with colors reflecting the burial condition, and shapes reflecting the associated PMI. (D) Pearson’s correlation coefficient (ρ) matrix with hierarchically clustered dendrograms of unique proteins (*n* = 3910; Supporting Methods). As a normalized measure of covariance, ρ has a value between −1 (perfect negative correlation) and 1 (perfect positive correlation); however, note the restricted range illustrated (0.0–1.0), reflecting the fact that only positive correlations were observed between proteomic profiles.

To visualize these taphonomic trajectories, we ordinated samples by proteomic composition using PCA and UMAP. PCA separated early from late PMIs along the first principal component (Figure 2b), which explained 73.7% of the total variance, and showed tight clustering of early time points but greater dispersion among later time points. UMAP recapitulated this temporal ordering and, at late PMI, highlighted oxygenation as a major axis of divergence (Figure 2c): oxic samples extended the early-PMI trajectory, whereas hypoxic samples formed a distinct late-PMI cluster. Correlation analysis with hierarchical clustering supported this separation (Figure 2d), with stronger within-group similarity among late-hypoxic samples and greater variability among late-oxic samples. Together, these analyses indicate that oxygen availability is a dominant factor shaping late-stage proteomic composition, consistent with divergent taphonomic trajectories under oxic *vs* hypoxic burial.

### *Post-Mortem* Shifts in Brain Proteome Composition

We next asked how the functional composition of the brain proteome changes along different taphonomic trajectories. Using linear modeling with *k*-means and hierarchical clustering of protein abundance through time, we defined two major groups: proteins generally more abundant at early PMI across environments (*n* = 1886), and proteins relatively more abundant at late PMI in hypoxic conditions (*n* = 1112; Supporting information, Figures S4–S8).

Gene Ontology (GO) enrichment of these clusters revealed clear *post-mortem* shifts in biological processes, molecular functions, and cellular components (Figures S9–S11). Proteins peaking in abundance at early PMI were characterized by mRNA- and translation-related processes (e.g., peptide biosynthesis, ribonucleoprotein complex biogenesis), with molecular functions reflecting (m)­RNA binding, GTPase regulator activity, and structural constituents of the ribosome. Cellular component annotations were dominated by ribonucleoprotein assemblies, nuclear bodies, and chromosomal regions. By contrast, proteins peaking at late PMI were associated with energy and nucleotide metabolism (e.g., aerobic respiration, ATP metabolic process) and redox chemistry, with molecular functions dominated by oxidoreductase (including peroxidase) activities, cytoskeletal protein binding, and chaperone or receptor binding. Cellular component terms localized to membrane protein complexes—particularly mitochondrial and organelle inner membranes—as well as neuronal structures such as synapses, axons, and myelin, and to supramolecular fibers. Together, these results indicate a *post-mortem* progression from a relatively life-like, regulatory- and translation-focused intracellular proteome, to an enzyme-rich, membrane-associated proteome characterizing late-stage taphonomic trajectories.

### Peptide-Specific Decay Profiles in Different Environments

To resolve taphonomic trajectories at the peptide level, we fit >1.26 million peptide-specific decay profiles across environments and time points, and grouped peptides into labile, putative, and recalcitrant preservation classes based on data-driven thresholds applied to peptide-specific decay slopes (Supporting Information, Data S2). No peptides from wet–oxic samples met the quality criteria (all exhibited standard errors >90th percentile and/or detection frequencies <90th percentile; Supporting Methods) and were therefore excluded from peptide-level decay profiling; however, their low abundance and high variability at late PMI were themselves inconsistent with stability. Across the remaining conditions, labile peptides decayed rapidly, losing five log_2_ units of intensity over the PMI (Figure 3a), whereas putatively preserved peptides exhibited a shallower decline, with loss of two log_2_ units. By contrast, recalcitrant peptides evidenced little net loss overall and, in wet–hypoxic and dry–oxic conditions, increased in relative intensity with PMI. In dry–hypoxic conditions, recalcitrant peptides showed a modest early-to-mid PMI decrease, followed by a rebound to levels exceeding those at 24 h. In wet–hypoxic samples, labile peptides exhibited an initial phase of relative stability not evident in other environments, followed by accelerated loss after an estimated breakpoint around the first week *post-mortem*, although the timing approximated by segmented regression was uncertain. Thus, although the overall stability hierarchy (labile ≪ putative ≪ recalcitrant) was conserved, peptide decay profiles were modulated by burial environment.

**Figure 3:**
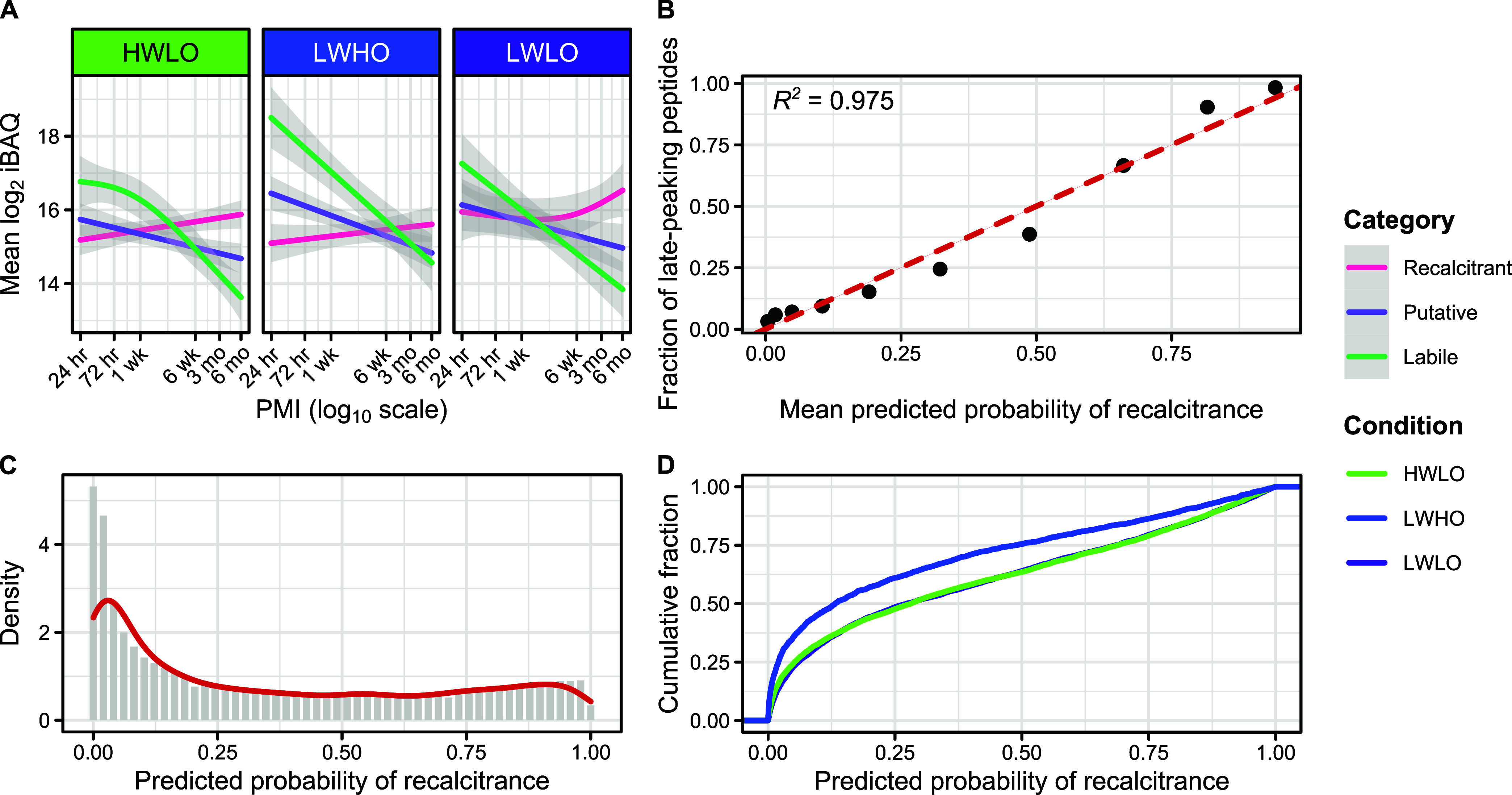
Peptide-specific decay profiles in different environments. No peptides detected in the wet–oxic condition met quality criteria, and were excluded from analyses. (A) Generalized additive model fits of mean log_2_ iBAQ *vs* PMI (log_10_ scale) for recalcitrant, putative, and labile peptides in each condition (*n* = 277,469 peptide–condition–time observations; 16,128 unique peptides; Supporting Methods). Solid lines represent the estimated smooth functions, and shaded ribbons denote 95% confidence intervals (CIs). (B) Scatter plot of the observed fraction of late-peaking peptides *vs* the mean predicted probability of recalcitrance, binned by prediction quantiles. Each point represents one bin of peptides sorted by their predicted recalcitrant score; the red dashed line is the ideal 1:1 calibration. The close alignment of points and high coefficient of determination (*R* ^2^ = 0.975) indicates that the model’s probability estimates accurately reflect the true likelihood of late-peaking behavior (Supporting Methods). (C) Distribution of predicted probabilities of recalcitrance. Gray bars show the density-scaled histogram of model-predicted scores (*n* = 50 bins), while the red curve is a smoothed kernel density estimate (Supporting Methods). (D) Empirical cumulative distribution of predicted probabilities of recalcitrance, stratified by burial condition. Each curve shows the fraction of peptides with a predicted probability ≤ *x* (Supporting Methods).

To quantify recalcitrance probabilistically, we fitted logistic regression models to peptide decay behavior and assessed classification performance with bootstrap resampling and precision–recall analysis (Figure S12). Calibration and discrimination metrics—including calibration intercept and slope, Brier scores, Spiegelhalter’s *Z*-statistics, and ROC analysis (Supporting Methods)—indicated excellent model performance (Figure S13), with predicted probabilities closely matching observed recalcitrance frequencies (Figure 3b). The predicted probability distribution was strongly right-skewed (Figure 3c): half of all peptides (50.1%) had low recalcitrance probability (*P* < 0.25), whereas a high-probability tail (*P* > 0.75) contained nearly one-fifth (19.6%) of peptides with strong preservation potential. Peptides from hypoxic conditions were enriched in this high-probability subset relative to oxic conditions (Figure 3d). Together, these models suggest that low-oxygen environments are associated with a larger fraction of peptides exhibiting decay profiles consistent with persistence and relative enrichment as more labile material is lost.

### Environmental Controls on Recalcitrant Peptide Classes

We then asked how recalcitrant peptides are distributed across environments, and from which protein classes they derive. Wet–hypoxic conditions yielded both the largest pool (*n* = 2647) and the highest proportion (37.4%) of recalcitrant peptides (Figure 4a), and hypoxic samples showed greater overlap in recalcitrant peptides with one another than was observed for peptides identified exclusively under oxic conditions. Over-representation analysis indicated that the protein classes corresponding to recalcitrant peptides differed across water- and oxygen--availability regimes (Figure 4b): hypoxic conditions yielded four times as many enriched classes as oxic conditions, with particularly strong enrichment of peroxidases, calcium-binding proteins, and metalloproteases (Supporting Information, Tables S4–S6). Despite most proteins being detected under all conditions (Figure S2b), only a small subset of peptides (*n* = 414) was recalcitrant in every environment; this shared set was enriched in oxidoreductases, proteases, and other metabolite interconversion enzymes. These enrichments suggest that recalcitrance is associated with preferential retention of peptides from proteins linked to redox chemistry, proteolysis, and metal or calcium binding.

**Figure 4:**
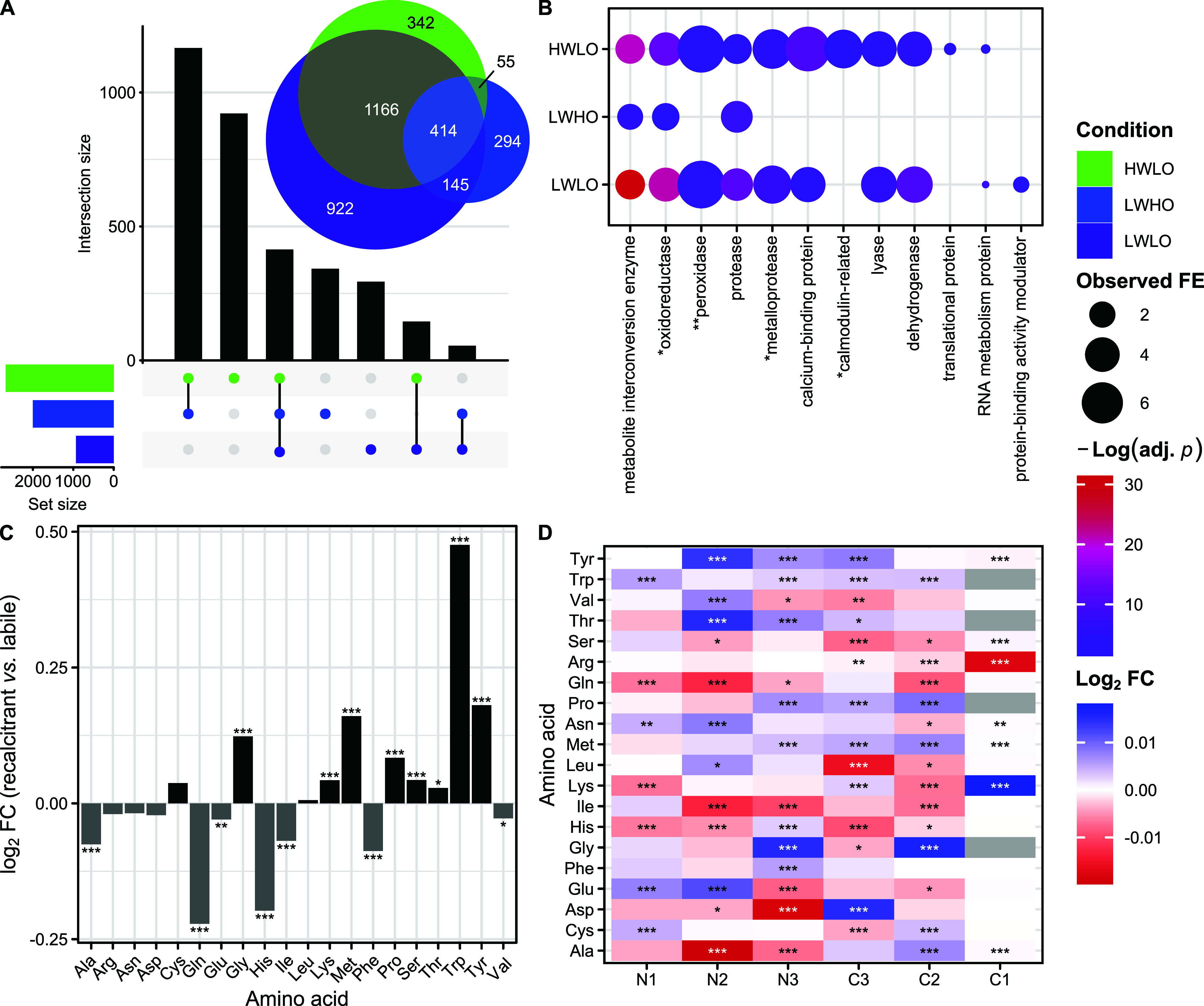
Protein features associated with recalcitrance. No peptides detected in the wet–oxic condition met quality criteria, and were excluded from analyses. (A) Upset plot of the number of unique recalcitrant peptides in each experimental condition (*n* = 3338). Horizontal bars indicate the total peptides per individual condition, connected dots denote the condition(s) contributing to each intersection, and vertical bars indicate intersection sizes. The inset is a scaled Euler diagram illustrating overlap in recalcitrant peptides across conditions: circle areas are proportional to the total peptides per individual condition, and numbers in overlapping regions indicate peptides shared between conditions. (B) Over-representation of protein classes among recalcitrant peptides: each point represents a protein class corresponding to recalcitrant peptides across experimental conditions (*n* = 3338; Supporting Methods). Point size represents the degree of fold enrichment (FE; i.e., observed *vs* expected numbers of proteins), where values <1 indicate under-representation. Point color denotes the −log_10_ of the adjusted *p*-value. Protein subclasses are indicated with an asterisk (*) to the right of parent classes, reflecting hierarchical relationships. Only statistically significant results are shown. (C) Bar chart of mean log_2_-fold change (log_2_ FC; recalcitrant *vs* labile) in amino acid frequency across peptides (*n* = 77,508; Supporting Methods). Values >0 indicate enrichment of a residue in recalcitrant peptides (*dark gray*), values <0 indicate depletion of a residue (*light gray*), and values = 0 indicate no difference in representation between recalcitrant and labile classes. (D) Heatmap showing positional amino acid enrichment at peptide termini (N1–N3, C1–C3) between recalcitrant and labile peptides (*n* = 77,508). Log_2_-FC (recalcitrant *vs* labile) was estimated by linear modeling of one-hot encoded terminal residues (Supporting Methods), and adjusted significance indicated by the following codes: *** = *p*-value < 0.0001; ** = *p*-value ≤ 0.001; and * = *p*-value ≤ 0.05.

### Physicochemical, Sequence, and Structural Predictors of Peptide Recalcitrance

We first investigated whether simple physicochemical properties could explain peptide recalcitrance. Hydropathy evidenced small, context-dependent differences under hypoxia, but overall effect sizes were negligible, and net charge and isoelectric point did not distinguish preservation categories (Supporting Information, Figure S14 and Tables S7–S9). Peptide-recovery metrics were similarly weak discriminators: mean peptide length and protein sequence coverage differed only modestly between preservation types and, although contrasts were strongest in wet–oxic conditions when analyses were stratified by environment, effect sizes remained small (Figure S15 and Tables S10–S12). Overall, averaged physicochemical properties and recovery metrics explain little of the variance in peptide persistence across burial environments.

Sequence and predicted structural metrics, however, showed a strong separation between preservation categories. Recalcitrant peptides were enriched in redox-active aromatic and sulfur-containing residues (Trp, Tyr, and Met) as well as Gly and Pro, and were depleted in residues intrinsically susceptible to hydrolysis, destructive oxidation, or fragmentation, particularly Gln and His (Figure 4c). Positional analyses further revealed small but reproducible biases near peptide termini (Figure 4d), indicating that decay resistance depends not only on overall amino acid composition but also on the positional distribution of residues within peptides. These compositional and positional patterns are compatible with the selective survival of structurally constrained peptides enriched in residues capable of stabilizing or terminating local radicals.

Recalcitrant peptides were more likely to be β-sheet-dominant (50.2% *vs* 40.8%) and less likely to be α-helix-dominant (48.3% *vs* 57.8%) than labile peptides, with coil-dominant peptides rare in both categories (<1.5%). Logistic regression confirmed a strong negative association between α-helical content and recalcitrance, alongside positive associations with β-sheet content and higher predicted structural confidence (Tables S13–S16). Recalcitrant peptides also exhibited lower predicted intrinsic disorder than labile and putative peptides, particularly under hypoxic conditions (Tables S17–S19). These results suggest that decay resistance is better predicted by local structure and residue chemistry than by averaged physicochemical properties, and show that recalcitrant peptides are biased toward β-sheet-rich, structurally ordered sequences enriched in redox-active residues. Several of these intrinsic sequence and structural features are also characteristic of peptides that become selectively stabilized in neurodegenerative proteinopathies,^41,42^ pointing to a potential parallel at the level of molecular architecture between peptide recalcitrance and pathological protein stabilization.

### Protein Features and Modifications Associated with Recalcitrance

We next investigated how peptide recalcitrance relates to protein features by annotating recovered peptides with UniProt-derived information on protein topology, domains, regions, and PTMs. Because UniProt annotations are hierarchical, we first assessed feature bias across higher annotation levels, which revealed widespread but weak associations with preservation category (Supporting Information, Tables S20 and S21); these effects largely reflect aggregation across many heterogeneous features, and are not individually informative. We therefore focused on descriptor-level annotations, where a restricted subset of features exhibited large and robust associations with recalcitrance (*n* = 18; Data S3). At this level, extracellular domains were three-fold enriched in recalcitrant peptides, whereas cytoplasmic and mitochondrial domains were three-fold enriched in labile peptides. Calcium-dependent phospholipid-binding domains evidenced the strongest domain-level biases, being approximately four- to 16-fold more common in recalcitrant than labile peptides.

Finally, we examined how PTMs differed between early and late PMI among different burial environments (Figure S16). In wet–oxic conditions, which demonstrated the most extensive molecular loss, late-PMI peptides exhibited up to five-fold increased odds of oxidative and alkylative modifications, alongside reduced sodium and calcium adduction. In hypoxic conditions, late-PMI peptides evidenced approximately two-fold increased odds of dioxidation events—particularly on Trp and Met—and of Trp-to-kynurenine conversion specifically under wet–hypoxic burial. Taken together, the enrichment of calcium-dependent phospholipid-binding domains and distinct oxidative modification profiles under hypoxia indicate that peptide recalcitrance is associated with specific protein domains and redox-related PTMs, rather than reflecting nonspecific resistance to decay. Given redox- and metal-mediated chemical processes involving aromatic residues are also implicated in molecular change during neurodegeneration,^43^ these results raise the possibility that *post-mortem* peptide persistence and *in vivo* neurodegenerative change may be shaped by overlapping reaction chemistries operating in very different biological contexts.

## Discussion

Brains are among the most decay-prone organs after death, yet are disproportionately preserved as the sole surviving soft tissue in waterlogged, oxygen-poor burial environments.^1^ Previous work on a single archeological brain has struggled to identify a unifying preservation mechanism, being based principally on protein-level abundance^7^ or on a small number of abundant proteins.^44^ By fitting more than 1.26 million peptide-level decay profiles across multiple experimental conditions and PMIs, our results show that this paradox reflects a distinct taphonomic trajectory—that is, a discrete *post-mortem* pathway of molecular transformation—to which nervous tissue is uniquely predisposed in wet–hypoxic conditions. This trajectory emerges consistently across the experiment and is defined by convergent sequence, structural, domain, and modification signatures that are most parsimoniously explained by radical-mediated oxidative cross-linking within membrane-associated microenvironments.

A key implication of our time-resolved, peptide-specific decay profiling approach is that chemical regimes associated with molecular preservation are established early in decomposition, on the order of weeks *post-mortem*. The brain proteome initially follows a broadly similar trajectory across burial conditions, characterized by early autolytic and hydrolytic fragmentation; with advancing PMI, however, oxygen availability becomes the dominant determinant of molecular fate. Oxic conditions are associated with accelerating protein loss, whereas hypoxic conditions evidence greater proteomic retention, tighter clustering in ordination space, and the selective persistence of a subset of recalcitrant peptides. This divergence is accompanied by condition-dependent patterns of oxidative modification, consistent with an early established, oxidative cross-linking pathway that subsequently constrains longer-term molecular preservation. Studies of deep-time fossilization have similarly proposed that oxidative cross-linking in the immediate PMI may stabilize soft tissues before complete degradation occurs;^9,10^ our results suggest that such stabilization need not outpace decay as a separate, antithetical process but rather that preservation can emerge from decay itself when oxidative chemistry proceeds under diffusion-limited, oxygen-restricted conditions.

Crucially, water and oxygen availability together appear to influence whether oxidation proceeds as a chain-propagating process that progressively accumulates damage, or as a self-limiting sequence of local initiation–termination events (Supporting Discussion and Table S22). When oxygen is readily available in an aqueous environment, oxygen-dependent radical propagation is more readily maintained,^45^ increasing the likelihood of chain-amplified oxidative fragmentation and widespread molecular loss. When oxygen is restricted, however, radical propagation is less likely to be sustained and oxidation more likely to be self-limiting,^45^ with termination occurring at the site of radical generation by quenching (e.g., with Met) or covalent coupling with nearby reactive groups (e.g., Trp, Tyr).^46^ The selective preservation of brain tissue in waterlogged, oxygen-poor burials may therefore reflect a chemically constrained regime in which hydration facilitates radical chemistry, while hypoxia prevents its escalation into wholesale destruction.

Within this regime, preservation is not predicted by physicochemical properties (such as hydropathy and charge) but rather by compositional, structural, and domain biases (Supporting Information). Recalcitrant peptides are enriched in large aromatic amino acids (Trp, Tyr) and sulfur-containing Met, and depleted in polar amides (Gln), imidazole-containing His, and small aliphatic residues (Ala, Val, Ile, Leu). These biases are consistent across environments but are most pronounced under hypoxia. The chemical significance of this pattern is that Trp and Tyr readily form stabilized radical intermediates and oxidation products capable of participating in covalent cross-linking,^47^ while Met can buffer oxidative flux and modulate local radical availability^48^ (Table S23). In contrast, Gln and His are disposed toward deamidation^49^ and metal-catalyzed self-oxidation,^50^ respectively, which promote fragmentation and solubilization. Importantly, modification data independently support this interpretation: oxic conditions are dominated at late PMI by more extensive oxidative and alkylative damage, whereas hypoxic conditions feature increased Trp-to-kynurenine conversion and dioxidation events, particularly localized to aromatic residues.

In wet–hypoxic conditions, recalcitrant peptides are enriched in β-sheet structure, depleted in α-helical structure, and exhibit higher predicted structural confidence and lower intrinsic disorder. Whereas α-helices are generally more flexible and solvent-exposed,^51^ β-sheets are densely hydrogen-bonded, closely packed, and relatively resistant to enzymatic and hydrolytic attack;^52^ and their enrichment among recalcitrant peptides is consistent with the durability of fibrillar and amyloid-like protein architectures in both biological^53,54^ and archeological^55,56^ contexts. Notably, accumulation of β-sheet-rich, structurally ordered protein assemblies is also a defining feature of aggregates that accrue during normal aging and in neurodegenerative disease,^57^ reflecting the intrinsic stability and degradation resistance of these conformations.

Finally, recalcitrant peptides are depleted in cytoplasmic and mitochondrial domains and strongly enriched—by up to 16-fold—in calcium-dependent phospholipid-binding C2 domains. C2 domains mediate Ca^2+^-regulated binding of proteins to anionic phospholipids on membrane surfaces,^58^ and their enrichment indicates that peptide persistence is disproportionately associated with proteins stabilized at membranes, rather than within freely soluble intracellular compartments. Loss of ionic homeostasis and rising intracellular Ca^2+^ during *post-mortem* decay^6^ would be expected to further promote sustained membrane association of such proteins. Membrane-adjacent microenvironments are structurally constrained, concentrating redox-active substrates and catalysts (such as lipids and metals) while restricting diffusion and oxygen availability relative to the cytosol. In this context, radical oxidation is less likely to proceed *via* chain propagation, which requires continuous access to molecular oxygen,^59^ and more likely to locally terminate *via* cross-linking. Such cross-linking would be expected to reduce molecular mobility and solubility, sterically hinder enzymatic and hydrolytic attack, and promote the formation of insoluble aggregates resistant to degradation.^60^

A further factor likely reinforcing this taphonomic trajectory is the brain’s unusually large and heterogeneous reservoir of redox-active iron.^61^ Nervous tissue contains high iron concentrations relative to most other soft tissues, distributed across multiple pools such as heme proteins, ferritin-bound stores, and iron-rich compartments associated with mitochondria, myelin, and oligodendrocytes.^62^ In life, these pools are tightly regulated to support oxidative metabolism while limiting collateral damage.^63^ After death, however, progressive membrane failure and loss of regulatory control are expected to alter iron speciation and availability, increasing the likelihood of local metal-catalyzed radical generation. Importantly, such chemistry need not produce uniform oxidative destruction: when redox reactions involving iron occur within membrane-adjacent or diffusion-limited microenvironments, they may favor the formation of short-lived aromatic radicals that terminate by covalent cross-linking rather than by chain-propagating oxidation (Table S22). The enrichment of peroxidases—which often involve heme iron or metal cofactors^64^—among recalcitrant peptides is compatible with localized, metal-associated redox chemistry, in which iron-containing cofactors may contribute to peroxide-driven radical formation without sustaining chain-propagating oxidation.

The brain is particularly predisposed to follow this trajectory. In life, it is among the most oxidatively stressed organs: it consumes a disproportionate share of oxygen, is rich in redox-active metals, and relies heavily on antioxidant and repair systems to maintain protein integrity.^65^ Additionally, the brain combines extreme membrane density,^66^ an abundance of structurally stable, long-lived proteins that accumulate heterogeneous oxidative modifications during life,^67^ and anatomical sequestration within the cranial vault. Together, these features establish a *post-mortem* environment characterized by pre-existing chemical and structural heterogeneity, limited molecular mobility, and restricted oxygen exchange: conditions that favor local, diffusion-limited radical reactions and termination by cross-linking rather than runaway, chain-propagating oxidation. Notably, the molecular features that define this *post-mortem* pathway closely parallel those that stabilize aggregation-prone protein assemblies in neurodegenerative disease: enrichment of β-sheet and structurally ordered fragments,^68^ redox-active residue modifications,^69^ and oxidative cross-links^70^ are hallmarks of pathological protein aggregation *in vivo*. While the biological contexts differ fundamentally, these parallels indicate that common chemical processes govern protein persistence across clinical and geological time scales.

## Conclusions

This study provides a molecular explanation for the long-standing paradox of selective brain preservation by demonstrating that waterlogged, oxygen-poor conditions divert the brain proteome onto a distinct taphonomic trajectory, characterized by radical-mediated oxidative cross-linking in membrane-associated microenvironments. We show that water and oxygen availability together determine whether *post-mortem* oxidation proceeds as a chain-propagating process that drives widespread proteomic loss, or as a diffusion-limited sequence of local radical reactions that favors persistence of a defined subset of peptides. The brain’s unique confluence of biochemical, structural, and anatomical features restricts molecular diffusion and oxygen availability, concentrating radical chemistry at membrane-adjacent compartments where termination by cross-linking is favored. As a result, peptide recalcitrance is not idiosyncratic but probabilistic, and can be predicted from environmental conditions. The resulting molecular signatures—ordered β-sheet structure and aromatic residue modifications consistent with local radical termination—mirror those observed in pathological protein stabilization during brain aging and neurodegeneration, underscoring how constrained oxidative chemistry can generate stability across disparate biological and temporal contexts. More broadly, this study shows that decay is not the opposite of preservation but, under specific chemical constraints, one of its mechanisms: the same oxidative reactions that drive molecular destruction can also generate molecular stability.

## Limitations

Two aspects of our experimental design warrant explicit consideration. First, carcasses were flash-frozen in liquid N_2_ and stored at −80 °C prior to burial, a step without a natural precedent. Although such handling is routine in proteomics and likely exerts limited influence on the proteomic profile relative to larger environmental effects,^71–74^ freezing inevitably alters cellular architecture and protein conformation to some degree. However, given all carcasses underwent identical handling and storage procedures, any freeze–thaw effects were systematic across conditions, preserving the validity of cross-condition comparisons. The principal implication is thus one of ecological realism, rather than experimental comparability.

Second, oxygen availability was operationally manipulated through atmospheric access, but dissolved oxygen and redox conditions were not directly monitored during decay. Recent experimental taphonomy studies^75,76^ demonstrate that decaying carcasses can rapidly consume dissolved oxygen from their immediate surroundings, including in systems open to atmospheric diffusion, reaching hypoxic or anoxic conditions within hours to days. It is therefore possible that all conditions became effectively oxygen-depleted at the carcass–matrix interface within the first two time points (24 and 72 h), regardless of bulk oxygen availability. Accordingly, our oxic treatments should be understood as operational contrasts in atmospheric exchange and oxygen replenishment, rather than as continuously oxygenated systems throughout the PMI.

Importantly, this does not undermine—and may in fact reinforce—the wet–hypoxic taphonomic trajectory we describe. Our mechanistic model holds that preservation depends not on a simple binary distinction between oxic and anoxic conditions, but on whether oxidative radical chemistry is permitted but constrained. If rapid, carcass-driven oxygen drawdown early in the PMI was common to all wet conditions, as these recent studies suggest,^75,76^ the divergence observed between wet–oxic and wet–hypoxic burial would then reflect differences in the rate and extent of oxygen depletion, with slower or less complete drawdown in open systems permitting a longer interval of sustained chain-propagating oxidation sufficient to drive greater molecular fragmentation and loss. Further work integrating real-time redox monitoring with proteomic profiling at the carcass–matrix interface will refine the temporal and spatial dynamics of this transition.

## Supplementary Material









## Data Availability

The LC–MS/MS data have been deposited to the ProteomeXchange Consortium *via* the PRIDE^77^ partner repository with the data set identifier, PXD074190.^78^

## Notes

Supporting Results; Discussion; Methods; Proteins recovered by experimental condition and time point (Table S1); Protein concentration during decomposition (Figure S1); Proteomic profiles by burial environment (Figure S2); Proteomic profiles by time point (Figure S3); Protein abundance at different time points (Table S2); Protein abundance in different burial environments (Table S3); Complex heatmap of scaled protein iBAQ data with integrated linear modeling, *k*-means and hierarchical clustering of proteins globally (Figure S4); Complex heatmap of scaled protein iBAQ data with integrated linear modeling, *k*-means and hierarchical clustering of proteins in wet–oxic conditions (Figure S5); Complex heatmap of scaled protein iBAQ data with integrated linear modeling, *k*-means and hierarchical clustering of proteins in wet–hypoxic conditions (Figure S6); Complex heatmap of scaled protein iBAQ data with integrated linear modeling, *k*-means and hierarchical clustering of proteins in dry–oxic conditions (Figure S7); Complex heatmap of scaled protein iBAQ data with integrated linear modeling, *k*-means and hierarchical clustering of proteins in dry–hypoxic conditions (Figure S8); Biological process enrichment of proteins at early and late PMI (Figure S9); Molecular function enrichment of proteins at early and late PMI (Figure S10); Cellular component enrichment of proteins at early and late PMI (Figure S11); Precision–recall for early- and late-peaking peptide classifiers across conditions (Figure S12); Calibration and discrimination of the peptide-level recalcitrance classifier (Figure S13); Over-representation test results for PANTHER protein class corresponding to recalcitrant peptides identified in wet–hypoxic conditions (Table S4); Over-representation test results for PANTHER protein class corresponding to recalcitrant peptides identified in dry–oxic conditions (Table S5); Over-representation test results for PANTHER protein class corresponding to recalcitrant peptides identified in dry–hypoxic conditions (Table S6); Hydropathy by burial environment (Figure S14); Hydropathy by preservation category (Table S7); Net charge by preservation category (Table S8); Isoelectric point by preservation category (Table S9); Sequence coverage distributions by preservation category within burial environments (Figure S15); Mean peptide length and sequence coverage by preservation category (Table S10); Mean peptide length by preservation category (Table S11); Sequence coverage by preservation category (Table S12); Secondary structure by preservation category (Table S13); Weighted α-helix fraction by preservation category and burial environment (Table S14); Weighted β-sheet fraction by preservation category and burial environment (Table S15); Predicted secondary structure model confidence by preservation category (Table S16); Intrinsic disorder by preservation category (Table S17); Mean IUPred score by preservation category and burial environment (Table S18); Fraction disordered by preservation category and burial environment (Table S19); UniProt feature section by preservation category (Table S20); UniProt feature type by preservation category (Table S21); Odds of modification at late *vs* early PMI by burial environment (Figure S16); Dominant radical oxidation pathways under contrasting hydration and oxygen regimes (Table S22); Reactivity and preservation potential of key amino acid residues (Table S23); Sample mass and protein concentration for the DDA data set (Table S24); Sample mass and protein concentration for the DIA data set (Table S25); Determination of optimal number of clusters for hierarchical clustering analysis of iBAQ data (Figure S17) (PDF)
Log_2_-transformed peptide intensities derived from library-free DIA; Log_2_-transformed intensity-based absolute quantification (iBAQ) values at the protein-group level (XLSX)
Peptide-level decay modeling and classification data derived from log_2_-transformed peptide intensities (XLSX)
Descriptor-level UniProtKB feature enrichment data (XLSX)
