# Supplementary material for: Molecular Solution to the Paradox of Ancient Brain Preservation
Source: J Proteome Res. 2026 Jul 3;25(8):4189–201. doi: 10.1021/acs.jproteome.6c00200 (PMC13459548; doi:10.1021/acs.jproteome.6c00200)
Supplement: Supplementary file 1 [file pr6c00200_si_001.pdf]

Supplementary to: Molecular solution to the paradox of ancient brain preservation

Alexandra Morton-Hayward[1,2,3*], Sarah Flannery[2], Peter Berry[4], Iolanda Vendrell[2], Anders Johansen[3], Martin Hansen[5], & Roman Fischer[2]

[1] *Department of Earth Sciences, University of Oxford; 3 South Parks Road, Oxford, OX1 3AN UK*
[2] *Centre for Medicines Discovery, Nuffield Department of Medicine, University of Oxford; Roosevelt Drive, Oxford, OX3 7FZ UK*
[3] *Department of Environmental Science, Aarhus University; Frederiksborgvej 399, DK-4000 Roskilde, Denmark*
[4] *Centre for Proteome Research, Institute of Systems, Molecular and Integrative Biology, University of Liverpool; Crown Street, Liverpool, L69 7ZB UK*
[5] *Department of Environmental Science and Resource Engineering, DTU Sustain, Technical University of Denmark; Bygningstorvet 115, DK-2800 Kongens Lyngby, Denmark*

**** Corresponding author.** Email: mert4520@earth.ox.ac.uk | Phone: +441865 272000*

Results **3**
Divergent taphonomic trajectories under oxic and hypoxic burial 3
Post-mortem shifts in brain proteome composition 3
Environment-specific peptide decay trajectories and recalcitrance 4
Environmental controls on recalcitrant peptide classes 4
Physicochemical, sequence, and structural predictors of peptide recalcitrance 5
Protein topology, domains and modifications associated with recalcitrance 6
Discussion **7**
Role of water in enabling radical-mediated oxidative crosslinking under hypoxia 7
Role of reactive residues in radical-mediated oxidative crosslinking 7
Methods **8**
Experimental taphonomy 8
Proteomics 10
Data-dependent acquisition 10
Data-independent acquisition 10
Data analysis 11
Protein identification 11
Ordination and differential expression analysis 12
Peptide preservation modelling and classification 12
Biochemical correlates of peptide preservation 12
Compositional and structural correlates of peptide preservation 13
Functional feature analyses 13
Post-translational modification analysis 14
Figures & Tables **15**
Table S1. Proteins recovered by experimental condition and time point 15
Fig S1. Protein concentration during decomposition 16
Fig S2. Proteomic profiles by burial environment 17
Fig S3. Proteomic profiles by time point 18
Table S2. Protein abundance at different time points 18
Table S3. Protein abundance in different burial environments 19
Fig S4. Complex heatmap of scaled protein iBAQ data with integrated linear modelling, *k*-means and hierarchical clustering of proteins globally 20
Fig S5. Complex heatmap of scaled protein iBAQ data with integrated linear modelling, *k*-means and hierarchical clustering of proteins in wet–oxic conditions 21
Fig S6. Complex heatmap of scaled protein iBAQ data with integrated linear modelling, *k*-means and

hierarchical clustering of proteins in wet–hypoxic conditions 22
Fig S7. Complex heatmap of scaled protein iBAQ data with integrated linear modelling, k-means and hierarchical clustering of proteins in dry–oxic conditions 23
Fig S8. Complex heatmap of scaled protein iBAQ data with integrated linear modelling, k-means and hierarchical clustering of proteins in dry–hypoxic conditions 24
Fig S9. Biological process enrichment of proteins at early and late PMI 25
Fig S10. Molecular function enrichment of proteins at early and late PMI 26
Fig S11. Cellular component enrichment of proteins at early and late PMI 27
Fig S12. Precision–recall for early- and late-peaking peptide classifiers across conditions 28
Fig S13. Calibration and discrimination of the peptide-level recalcitrance classifier 29
Table S4. Over-representation test results for PANTHER protein class corresponding to recalcitrant peptides identified in wet–hypoxic conditions 29
Table S5. Over-representation test results for PANTHER protein class corresponding to recalcitrant peptides identified in dry–oxic conditions 30
Table S6. Over-representation test results for PANTHER protein class corresponding to recalcitrant peptides identified in dry–hypoxic conditions 30
Fig S14. Hydropathy by burial environment 31
Table S7. Hydropathy by preservation category 31
Table S8. Net charge by preservation category 32
Table S9. Isoelectric point by preservation category 32
Fig S15. Sequence coverage distributions by preservation category within burial environments 34
Table S10. Mean peptide length and sequence coverage by preservation category 34
Table S11. Mean peptide length by preservation category 35
Table S12. Sequence coverage by preservation category 35
Table S13. Secondary structure by preservation category 36
Table S14. Weighted α-helix fraction by preservation category and burial environment 36
Table S15. Weighted β-sheet fraction by preservation category and burial environment 37
Table S16. Predicted secondary structure model confidence by preservation category 38
Table S17. Intrinsic disorder by preservation category 38
Table S18. Mean IUPred score by preservation category and burial environment 38
Table S19. Fraction disordered by preservation category and burial environment 39
Table S20. UniProt feature section by preservation category 40
Table S21. UniProt feature type by preservation category 40
Fig S16. Odds of modification at late *vs*. early PMI by burial environment 41
Table S22. Dominant radical oxidation pathways under contrasting hydration and oxygen regimes 41
Table S23. Reactivity and preservation potential of key amino acid residues 42
Table S24. Sample mass and protein concentration for the DDA dataset 43
Table S25. Sample mass and protein concentration for the DIA dataset 46
Fig S17. Determination of optimal number of clusters for hierarchical clustering analysis of iBAQ data 49
References 50

Results

Divergent taphonomic trajectories under oxic and hypoxic burial

The mean number of identified proteins decreased as decomposition progressed (Data S1; Table S1). The mean protein concentration at 72 hours (mean [SD] = 8.74 [0.79] µg/µL) was slightly higher than at 24 hours (8.64 [1.26] µg/µL; Fig S1), which may reflect accumulation of degraded cellular and molecular components during early autolysis [1]; however, the difference was non-significant ($p = .817$). The number of proteins recovered declined over time (Fig S3a), with the highest mean at 24 hours (3,607 [78]) and the lowest at six months (1,409 [1,007]). The > 10-fold increase in SD between these two time points suggested increasing variation with advancing decay. Most unique proteins (69.6%, 2,722/3,910) were identified at every time point (Fig S3b), but statistically significant differences were observed between "early" (< six weeks) and "late" (≥ six weeks) PMIs (Table S2). Intersections spanning non-adjacent time points were rare (8.5%, 7/82; e.g., two proteins identified only at 72 hours and six months), indicating that sampling intervals were sufficient to resolve the majority of proteomic changes. Significant pairwise differences in protein recovery between burial environments were confined to comparisons involving the wet–oxic condition (Table S3): this condition yielded the lowest mean number of proteins (2,231 [1,411]) and the dry–hypoxic condition the highest (2,939 [693]; Fig S2a), yet almost all unique proteins were identified in every condition (93.8%, 3,667/3,910; Fig S2b). Temporal profiles of each condition suggested an apparent "pseudo-enrichment" of proteins at the latest PMI under hypoxia (Main Text, Fig 2a), with mean counts at six months approximately one-tenth higher than at three months (wet–hypoxic: 2,024 [105] *vs.* 1,813 [80]; dry–hypoxic: 2,312 [374] *vs.* 2,048 [69]). Under oxia, the opposite pattern was observed: in dry–oxic conditions the mean at six months was approximately half that at three months (1,220 [967] *vs.* 2,346 [393]), whereas in wet–oxic samples it was an order of magnitude lower (81 [70] *vs.* 1,016 [503]).

Complementary ordination approaches further elucidated both the temporal shifts in proteomic profiles during decomposition, and the environmental factors shaping them. Principal component analysis (PCA) demonstrated a clear separation between early and late PMIs (Main Text, Fig 2b), with decomposition progression (PC1) explaining almost three-quarters of the variation in the dataset (73.7%). Early time points (24 hours, 72 hours, and one week) clustered tightly, pointing to a relatively stable proteomic landscape, whereas late time points (six weeks, three months, and six months) were more dispersed, suggesting increased variability with advancing decay. Uniform manifold approximation and projection (UMAP) recapitulated this temporal trend, and additionally highlighted the influence of oxygen availability (Main Text, Fig 2c): at late PMI, oxic samples followed the continuous trajectory of early PMI samples, indicating a gradual proteomic shift, whereas hypoxic samples diverged into a distinct cluster, consistent with an alternate taphonomic trajectory. Correlation analysis with unsupervised hierarchical clustering corroborated these observations, splitting later PMIs into two major groupings by burial oxygenation (Main Text, Fig 2d): hypoxic samples evidenced strong within-group correlations (e.g., wet–hypoxic *vs.* dry–hypoxic at 6 months: $\rho_{mean} = 0.77$), whereas oxic samples were more variable (e.g., wet–oxic *vs.* dry–oxic at 6 months: $\rho_{mean} = 0.13$).

Post-mortem shifts in brain proteome composition

Linear modelling with k-means and hierarchical clustering identified two major groups (Fig S4). The first ($n = 1{,}886$) comprised proteins generally more abundant at early PMI in all conditions; the second ($n = 1{,}112$) evidenced a more complex pattern, with proteins in hypoxic conditions relatively more abundant at late PMI. These trends were confirmed by per-condition analyses (Figs S5–8). GO enrichment of global k-means clusters revealed significant decay-associated shifts in biological process (Fig S9), molecular function (Fig S10), and cellular component (Fig S11) terms. Proteins peaking at early PMI were characterised by mRNA- and translation-related processes (e.g., peptide biosynthesis, ribonucleoprotein complex biogenesis), with molecular functions reflecting (m)RNA binding, GTPase regulator activity, and structural constituents of the ribosome. Cellular component annotations were dominated by ribonucleoprotein assemblies, nuclear bodies, and chromosomal regions. By contrast, proteins peaking at late PMI were associated with energy and nucleotide metabolism (e.g., aerobic respiration, ATP metabolic process) and redox chemistry. Molecular functions centered on oxidoreductase (including peroxidase) activities, cytoskeletal protein binding, chaperone/heat-shock protein binding, and receptor binding. Cellular component terms localised to membrane protein complexes – particularly mitochondrial and organelle inner membranes – as well as neuronal structures (e.g., glutamatergic synapse, axon, myelin sheath) and supramolecular fibres. Together, these results

indicate a clear *post-mortem* progression from a relatively life-like, regulatory- and translation-focused intracellular proteome, to an enzyme-rich, membrane- and extracellular-associated proteome with advancing PMI.

Environment-specific peptide decay trajectories and recalcitrance

The recalcitrance (i.e., decay resistance) of proteomic profiles was investigated by determining peptide-specific decay rates (Data S2), which varied by experimental condition. No wet–oxic peptides met the quality criteria and were excluded from investigation; however, their low late-PMI abundance (detection frequency < 90th percentile) and high variability (SE > 90th percentile) were inconsistent with stability. Across other conditions, labile peptides decayed rapidly (mean slope [SD] = -1.73×10^{-2} [7.08×10^{-3}]), losing approximately five $\log_2$ units of intensity (18.1–13.4) across the PMI (Main Text, Fig 3a). Peptides of putative preservation type exhibited a shallower decline, losing two $\log_2$ units during the same period (16.4–14.4). Recalcitrant peptides showed little net loss overall (4.28×10^{-3} [5.36×10^{-3}]): in wet–hypoxic and dry–oxic conditions, their relative abundance increased with PMI (3.47×10^{-3} [5.43×10^{-3}]), and in dry–hypoxic conditions, an initial modest decrease in intensity from early- to mid-PMI was followed by a rebound to levels exceeding those at 24 hours (15.0–17.1 $\log_2$ units). In wet–hypoxic samples, labile peptides exhibited an early phase of relative stability not evident in other conditions: segmented regression identified a breakpoint at approximately one week *post-mortem* (ψ [SE] = 0.84 [0.44] $\log_{10}$ PMI, corresponding to 6.9 days), although uncertainty around the timing was considerable (95% CI = 0.95–49.5 days). Regardless of the precise moment of inflection, the slope before this breakpoint did not differ from zero (estimated slope ± SE = –0.05 ± 1.25, p = .97), whereas after the breakpoint it became strongly negative (–2.09 ± 0.40, $p = 1.28 \times 10^{-7}$), suggesting accelerated peptide loss. Thus, although the overall stability hierarchy (labile ≪ putative ≪ recalcitrant) was conserved, peptide decay trajectories were modulated by burial environment.

Logistic regression was employed to estimate peptide-specific probabilities of recalcitrance, with bootstrap resampling (n = 1000) to assess calibration and overall fit (Figs S12–13). The calibration intercept was essentially zero (median [95% CI] = -2.85×10^{-16} [-7.75×10^{-15}, 7.76×10^{-15}]) and the slope was unity (1.000 [1.000, 1.000]), indicating no systematic mis-, over-, or under-prediction. The median Brier score – measuring mean squared error of the predicted probabilities, and thus ideally zero – was 0.113 [0.110, 0.117], consistent with strong overall accuracy. Spiegelhalter's Z-statistic for perfect calibration was also essentially zero (median [95% CI] = -3.32×10^{-12} [-1.67×10^{-11}, -6.29×10^{-13}], p = 1.00), and the calibration plot (Main Text, Fig 3b) yielded a high coefficient of determination ($R^2 = 0.975$). The predicted probability distribution was strongly right-skewed (Main Text, Fig 3c): whereas half of all peptides (50.1%) had low recalcitrance probability ($P < 0.25$), a distinct peak at high probabilities ($P > 0.75$) pinpointed a subpopulation (19.6%) with strong preservation potential. Hypoxic peptides were enriched in this high-probability tail (mean ≥ 0.75 = 20.7%) compared to oxic peptides (13.6%; Main Text, Fig 3d).

Environmental controls on recalcitrant peptide classes

Wet–hypoxic conditions yielded the largest pool (n = 2,647) and highest proportion (37.4%) of recalcitrant peptides, as well as the largest number identified in a single context (n = 922; Main Text, Fig 4a). Hypoxic samples evidenced the most extensive pairwise overlap (n = 1,166), almost four times the number identified exclusively in oxic samples (n = 294). Over-representation analysis indicated that the classes of proteins corresponding to recalcitrant peptides differed across water- and oxygen-availability regimes (Main Text, Fig 4b; Tables S4–6). Although > 90% of proteins were identified in all conditions (Fig S2b), only 414 peptides were recalcitrant in every burial environment: this shared set was enriched for oxidoreductases (mean fold enrichment [FE] = 2.88, $p = 5.57 \times 10^{-3}$), proteases (2.80, $p = 5.87 \times 10^{-5}$), and metabolite interconversion enzymes (2.15, $p = 5.73 \times 10^{-6}$). Hypoxic conditions evidenced four times as many enriched classes as oxic conditions (12 *vs.* 3), with the strongest enrichment for peroxidases (7.74, $p = 1.61 \times 10^{-2}$), followed by calcium-binding proteins (5.24, $p = 1.18 \times 10^{-4}$), and metalloproteases (4.79, $p = 2.37 \times 10^{-6}$).

Physicochemical, sequence, and structural predictors of peptide recalcitrance

A range of physicochemical properties were investigated as potential correlates of peptide decay resistance. In terms of hydropathy, all preservation types exhibited negative mean GRAVY scores, indicative of hydrophilicity (Fig S14); under hypoxia, however, labile peptides were slightly more hydrophobic than recalcitrant peptides in wet conditions (mean [SD] = –0.26 [0.70], $p = 2.16 \times 10^{-2}$), but more hydrophilic in dry conditions (–0.34 [0.72], $p = 4.36 \times 10^{-4}$).

Despite these pairwise differences reaching statistical significance, effect sizes were negligible (mean $\eta^2_H = 0.003$; Table S7), indicating that decay resistance accounted for little variation in hydropathy. No significant differences between preservation types were detected in terms of net charge (Table S8) or isoelectric point (Table S9).

Peptide-recovery metrics also exhibited limited discriminatory power. Mean peptide length and protein sequence coverage differed only weakly between preservation types ($p \leq 10^{-2}$; Table S10). Putative peptides had marginally shorter mean chain lengths (mean [SD] = 13.9 [4.7] amino acids) than labile (14.3 [4.2], $p = 1.86 \times 10^{-2}$) and recalcitrant peptides (14.4 [4.8], $p = .394$), but labile and recalcitrant peptides did not differ ($p = .208$), and all pairwise effect sizes were negligible ($\delta < 0.002$). Putative peptides also exhibited lower mean sequence coverage (9.2%) than labile (11.4%, $p = 2.38 \times 10^{-4}$) and recalcitrant peptides (12.3%, $p = 7.94 \times 10^{-4}$), which again did not differ from one another other ($p = 1.00$), with an overall negligible effect ($\eta^2_H = 0.006$). When analyses were stratified by burial environment (Tables S11–12), wet–oxic conditions yielded the strongest contrast between labile and recalcitrant peptides (mean peptide length: $p = 1.60 \times 10^{-4}$; sequence coverage: $p = 6.26 \times 10^{-10}$), with recalcitrant peptides evidencing the highest coverage (Fig S15a) with a long right-tailed distribution (Fig S15b). However, effect sizes remained small ($\delta < 0.039$), and cumulative distribution curves converged rapidly across preservation types and conditions (Fig S15c), with only minor divergence at low coverage, consistent with small effect sizes despite statistically significant contrasts.

Primary structure, however, differed markedly between preservation types (Main Text, Fig 4c). Recalcitrant peptides were strongly enriched for Trp ($\log_2$FC = 0.476, $p = 2.12 \times 10^{-55}$), Tyr (0.181, 4.55×10^{-29}), and Met (0.161, 1.17×10^{-13}); relatively enriched in Gly (0.123, 2.02×10^{-26}) and Pro (0.084, 4.16×10^{-9}); and modestly enriched for Ser (0.043, 2.74×10^{-4}), Thr (0.028, 2.42×10^{-2}), and Lys (0.043, 5.75×10^{-5}). Recalcitrant peptides were strongly depleted in Gln (–0.222, 4.27×10^{-53}) and His (–0.198, 1.67×10^{-17}), and modestly depleted in Phe (–0.088, 3.31×10^{-9}), Ala (–0.075, 8.78×10^{-11}), Ile (−0.069, 1.84×10^{-8}), Val (–0.028, 1.40×10^{-2}), and Glu (−0.030, 5.57×10^{-3}). To assess whether these differences reflected site-specific biases near peptide ends, the analysis was repeated by position at peptide termini (Main Text, Fig 4d). At the N-terminus (N1), recalcitrant peptides were enriched in Glu ($\log_2$FC = 0.008, $p = 4.77 \times 10^{-4}$) and Trp (0.005, 3.87×10^{-10}), and depleted in Gln (–0.006, 7.78×10^{-6}) and Lys (–0.007, 1.04×10^{-7}). N2 evidenced enrichment of Thr (0.015, 1.26×10^{-17}), Tyr (0.014, 3.62×10^{-23}), and Glu (0.116, 1.47×10^{-10}), with depletion of Ala (–0.020, 1.75×10^{-23}). N3 was enriched for Gly (0.015, 6.07×10^{-14}), Thr (0.007, 5.11×10^{-5}), Pro (0.007, 5.69×10^{-5}) and Tyr (0.007, 4.19×10^{-6}), and evidenced depletion of Asp (–0.019, 3.79×10^{-19}) and Ile (–0.010, 2.94×10^{-7}). The C-terminus (C1) of recalcitrant peptides was dominated by Lys (0.018, 6.07×10^{-6}) and depleted in Arg (–0.018, 6.07×10^{-6}), consistent with tryptic termini. C2 evidenced enrichment of Gly (0.016, 9.69×10^{-16}), Pro (0.009, 9.04×10^{-10}), Met (0.007, 5.53×10^{-10}), and Ala (0.007, 6.98×10^{-4}), and depletion of Gln (–0.008, 1.42×10^{-6}). C3 evidenced enrichment of Asp (0.015, 1.89×10^{-17}), Tyr (0.008, 1.82×10^{-8}), Pro (0.005, 5.94×10^{-4}), and Met (0.005, 1.86×10^{-5}), and depletion of Leu (–0.016, 3.34×10^{-10}). Positional $\log_2$-fold changes were small but reproducible (n = 77,508 peptides), indicating subtle, site-specific primary-structure biases associated with decay resistance.

Secondary structure differed substantially between preservation categories (chi-squared association: $\chi^2[4] = 741.83$, $p = 3.06 \times 10^{-159}$). 57.8% of labile peptides were α-helix-dominant (standardised residuals, $|r| = +26.80$) and 40.8% β-sheet-dominant (–26.22). Conversely, recalcitrant peptides were enriched in β-sheets (50.2%, +20.58) and depleted in α-helices (48.3%, –20.44). Coil-dominant peptides were rare in both groups (recalcitrant: 1.5%, –0.38; labile: 1.4%, –2.58). Logistic regression of peptide recalcitrance as a function of secondary-structure composition, mean residue-level confidence scores (pLDDT), and peptide length (β-sheet as reference; n_{obs} = 96,768) confirmed a strong negative association of α-helical content with recalcitrance (coefficient ± SE = –0.539 ± 0.025, $p = 1.91 \times 10^{-106}$), a weaker negative effect of peptide length (–0.027 ± 0.002, 1.05×10^{-38}), and a strong positive effect of mean pLDDT (0.027 ± 0.001, 1.96×10^{-190}). Coil fraction exhibited a weak positive effect (0.186 ± 0.070, 7.86×10^{-3}) that became non-significant when restricting to high-confidence predictions (mean pLDDT > 70). All other effects persisted after controlling for model confidence and sampling noise (Table S13). Kruskal–Wallis tests likewise indicated that the α-helix and β-sheet weighted fractions and mean pLDDT differed significantly between labile and recalcitrant peptides in every burial condition ($p \leq 10^{-6}$; Tables S14–16). Together, these analyses indicated that peptides with higher α-helical content (and, to a lesser extent, greater length) are less likely to persist, whereas those

with higher β-sheet content and greater predicted structural confidence are most strongly associated with *post-mortem* stability.

Although 97% of all peptides exhibited high predicted model confidence (mean pLDDT ≥ 70; n = 87,588), recalcitrant peptides evidenced significantly higher scores (mean pLDDT [SD]: 93.4 [7.5]) than either putative (92.0 [9.1], $p = 2.44 \times 10^{-62}$) or labile (91.4 [9.5], $p = 5.30 \times 10^{-284}$) peptides, suggestive of greater predicted structural order. To assess whether this difference in pLDDT corresponded to differences in intrinsic disorder, peptide-level mean IUPred scores were compared among preservation categories. Median scores were low (< 0.170) across all categories, consistent with an overall predominance of ordered structure, but recalcitrant peptides evidenced significantly lower mean disorder (mean [SD] = 0.222 [0.170]) than putative (0.240 [0.186], $p = 7.27 \times 10^{-17}$) and labile peptides (0.244 [0.184], $p = 2.64 \times 10^{-71}$; Table S17). When stratified by environment, the labile–recalcitrant separation in disorder was present under hypoxia ($p \leq 10^{-13}$) but not in the oxic treatment (p = .742; Table S18), suggesting that peptide recalcitrance was associated with lower predicted disorder only under low-oxygen conditions. Analyses of the fraction of residues exceeding the disorder threshold (≥ 0.5) yielded congruent results (Table S19), indicating that these findings were robust to how disorder propensity was summarised.

Protein topology, domains, and modifications associated with recalcitrance

Peptide features also differed between preservation categories. At broad annotation levels, nearly all UniProt features evidenced significant associations ($p \leq 10^{-3}$; Tables S20–21), but effect sizes were small overall (Cramér's $V < 0.10$), indicating widespread but modest biases across protein topology, domains, regions, and PTMs. Descriptor-level analyses were performed on 72 UniProt features meeting a minimum observation threshold (Data S3): although many associations were nominally significant (a consequence of the large sample size; n_{obs} = 215,088), a restricted subset (n = 18) exhibited large and statistically robust effects ($p \leq 10^{-6}$, $V \geq 0.10$, OR ≥ 2). Extracellular domains were three-fold enriched in recalcitrant peptides (n = 8,724, $p = 5.47 \times 10^{-260}$, V = 0.28, OR = 3.42), whereas cytoplasmic and mitochondrial domains were three-fold enriched in labile peptides (cytoplasmic: n = 3,642, $p = 2.82 \times 10^{-193}$, V = 0.24, OR = 3.08; mitochondrial matrix: n = 708, $p = 9.19 \times 10^{-41}$, V = 0.11, OR = 2.77). Calcium-dependent phospholipid-binding domains evidenced the largest domain-level differences, being approximately four- to 16-fold more common in recalcitrant than labile peptides (C2: n = 354, $p = 2.11 \times 10^{-50}$, V = 0.10, OR = 6.08; C2 1: n = 258, $p = 1.35 \times 10^{-20}$, V = 0.06, OR = 3.77; C2 2: n = 342, $p = 3.31 \times 10^{-87}$, V = 0.13, OR = 15.68).

Finally, protein modification was investigated by estimating ORs for a given PTM at late PMI relative to early PMI (Fig S16). In wet–oxic conditions – those least conducive to preservation – the odds of oxidative replacement of a methylene group (O–CH_2, +1.98 Da) were nearly six times higher at late PMI (mean OR [95% CI] = 5.67 [1.56, 20.64]), and those of C_2H_3NO alkylation (+57.02 Da) were more than four times higher (4.09 [1.44, 11.6]). The odds of methylation (+14.02 Da) were also elevated (2.42 [1.60, 3.65]), whereas those of sodium (Na^+, +21.98 Da) and calcium adduction (Ca^+, +37.95 Da) were decreased (Na^+: 0.62 [0.51, 0.76]; Ca^+: 0.60 [0.41, 0.90]). Sodium adduction also exhibited reduced odds at late PMI in wet–hypoxic conditions, whereas the odds of Trp-to-kynurenine conversion (+4.00 Da) were almost three-fold higher (2.82 [1.45, 5.50]). In dry–hypoxic conditions, the odds of di-oxidation (+31.99 Da) were approximately twice as high at late PMI (2.04 [1.44, 2.89]), with two-thirds of all di-oxidation events (66.1%, 37/56) localised to Trp.

Discussion

Role of water in enabling radical-mediated oxidative crosslinking under hypoxia

Water availability appears to be a necessary co-determinant of the taphonomic trajectory identified here. In biological tissues, oxidative radical chemistry and subsequent crosslinking typically occur in aqueous media, such as the cellular environment [2]. More generally, the initiation, propagation, and termination of radical processes depend on proton-coupled electron transfer, metal coordination, and short-range molecular mobility [3]: these requirements are expected to be attenuated as water availability decreases, given dehydration suppresses the local transport and solvent-mediated interactions that facilitate the formation, persistence, and encounter with suitable reaction partners of reactive species on relevant timescales [4,5].

Crucially, water and oxygen availability together can influence whether oxidation proceeds as a chain-propagating process that progressively accumulates damage, or as a self-limiting sequence of local initiation–

termination events (Table S22). Aqueous environments can sustain limited oxidative chemistry even when oxygen is restricted [2]: residual dissolved oxygen, endogenous peroxides, and metal-mediated redox cycling (e.g., Fe^{2+}/Fe^{3+} interconversion; [6]) can persist in water and generate short-lived radical intermediates capable of oxidising redox-active residues such as Trp, Tyr, and Met [7]. When oxygen availability is low, however, radical oxidation is less likely to proceed efficiently *via* oxygen-dependent chain propagation [8]. Under such conditions, oxidation is more likely to take the form of spatially localised radical events whose dominant fate is termination, either by electron transfer to nearby sinks [7] or by covalent coupling with nearby reactive groups [9].

Water also modulates this balance between stabilising and destructive oxidation by controlling oxygen transport. In waterlogged substrates, oxygen diffusion is orders of magnitude slower than in air, promoting steep redox gradients and localised depletion [10]; such conditions would be expected to further suppress sustained chain propagation, which requires continuous access to molecular oxygen, and to favour isolated initiation–termination events [8]. By contrast, in wet–oxic environments, continued radical regeneration and peroxyl-radical formation are more readily sustained [2], increasing the likelihood of secondary reactions (such as backbone cleavage) that promote more widespread molecular loss [7]. Together, these considerations are consistent with wet–hypoxic environments occupying an intermediate chemical regime in which oxidation is neither fully absent, nor catastrophically destructive.

In addition to enabling redox chemistry, water supports limited conformational flexibility and local molecular rearrangement [4]. Partial hydration can permit side-chain reorientation and transient exposure of reactive groups [11], increasing the likelihood of encounter between short-lived radical intermediates and suitable coupling partners; in fully desiccated systems, however, such mobility is strongly suppressed [5]. In fully hydrated but oxygenated systems, radical lifetimes and effective chain length can be extended by continuous regeneration, favouring diffusion, interception by quenchers, and chain-propagating oxidation [2]. In fully hydrated but hypoxic systems, molecular mobility would be expected to facilitate radical termination by coupling [9], and low oxygen to limit propagation and promote local stabilisation [8].

Taken together, these considerations suggest that water is not merely permissive, but likely mechanistically important for the taphonomic trajectory described here. Radical-mediated oxidative crosslinking is most plausibly supported by an aqueous phase that enables redox cycling, molecular encounter, and termination by coupling, while low oxygen availability restricts escalation into chain-propagating fragmentation. The selective preservation of brain tissue in waterlogged, oxygen-poor environments may therefore reflect a chemically constrained regime in which hydration permits radical chemistry, while hypoxia prevents its escalation into wholesale molecular loss.

Role of reactive residues in radical-mediated oxidative crosslinking

The enrichment of Trp, Tyr, and Met in recalcitrant peptides is chemically consistent with their redox activity, and their propensity to form relatively stable oxidation products and radicals capable of secondary reactions, including radical-mediated oxidative crosslinking (Table S23). These residues are readily oxidised because their side chains contain high-electron-density functional groups – the indole ring of Trp, the phenolic ring of Tyr, and the thioether group of Met – with low ionisation potentials that facilitate electron donation to reactive oxygen species [12,13]. The ensuing oxidation products, however, tend to promote stabilisation rather than fragmentation [9,14]. Trp, with its conjugated indole system, undergoes stepwise one-electron oxidations yielding indoxyl, kynurenine, and quinonoid intermediates [15] that act as radical sinks, and can couple to neighbouring aromatic or carbonyl groups to form crosslinks and other stable adducts [16]. The almost three-fold increased odds of Trp-to-kynurenine conversion at late PMI under waterlogged, hypoxic burial conditions is consistent with preservation *via* Trp-centred oxidation pathways that generate comparatively stable products and long-lived radical intermediates.

Tyr's phenolic oxygen stabilises tyrosyl radicals by delocalisation over the aromatic ring, forming dityrosine and higher-order cross-links [17,18] that are chemically robust, hindering enzymatic cleavage [19] and accumulating as markers of oxidative ageing [20,21]. Met is easily oxidised because the sulfur atom's lone pairs participate in soft electrophilic interactions, forming sulfoxides or sulfones [22]; *post-mortem*, these reactions allow Met to act as a sacrificial redox buffer [23,24] that can modulate local radical availability. Transient sulfoxides can also generate thiyl radicals that might participate in secondary oxidative crosslinking with aromatic or carbonyl moieties [25].

Collectively, these reactions provide a plausible route by which oxidative energy is dissipated into radical quenching and oxidative crosslinking, increasing resistance to proteolysis and thereby favouring preservation.

Structural evidence supports this interpretation. Met–aromatic motifs are widespread in oxidoreductases, where Met, Trp, and Tyr cluster in defined geometries around redox-active metal centres [26,27]. *In vivo*, these motifs mediate electron transfer and regulate controlled oxidation [12]; *ex vivo*, analogous geometries may instead promote intra- and intermolecular radical chemistry, increasing the probability of oxidative crosslinking when reactive side chains are exposed by partial degradation, and reaction products have accumulated locally (e.g., within partially unfolded and diffusion-limited microenvironments). The prevalence of oxidoreductases and calcium-binding proteins among recalcitrant peptides is consistent with this model, as these classes are both metal-associated and structurally enriched in redox-active residues.

By contrast, residues depleted in recalcitrant peptides are chemically disposed either to destructive oxidation, or to inertness that disfavours stabilising reactions. The imidazole ring of His co-ordinates transition metals such as Fe^{2+} and Cu^{2+}, catalysing Fenton-like reactions that generate hydroxyl radicals [28]; these oxidise His to 2-oxo-histidine or ring-cleaved imidazolone derivatives [29], promoting peptide scission. Gln deamidates by direct hydrolysis in acidic conditions, and *via* cyclic imide intermediates in neutral or alkaline conditions [30]; the process converts a neutral amide into a negatively-charged carboxyl group, increasing solubility and susceptibility to hydrolytic cleavage. Phe, though aromatic, lacks heteroatoms capable of stabilising radicals and therefore does not readily support crosslink formation analogous to Trp and Tyr [31]. Small aliphatic residues (Ala, Val, Ile) undergo slow peroxidation to unstable hydroperoxides [32], which decompose by β-scission to reactive carbonyl species and other fragments [33]. Their under-representation in recalcitrant peptides likely reflects both this vulnerability to fragmentation, and their limited capacity for the oxidative crosslinking that stabilises surviving peptides.

Methods

Experimental taphonomy

All experimental procedures complied with the Danish Animal Experimentation Inspectorate (2020-15-0201-00432), and ethical approval was obtained from the Central University Research Ethics Committee at the University of Oxford (R80617/RE001). Male, specific pathogen-free mice (n = 72; C57BL/6JRj, Janvier Labs) were reared and sacrificed under identical conditions for the purposes of other scientific research [34], and were donated to our study. Mice were weaned 25 days after birth, then fed on a low-fat defined control diet (D12328, Research Diets, Inc.), with body weight measured weekly. Mice were maintained in individually ventilated cages at 22 ± 1 °C on a standard 12 hour light–dark cycle with *ad libitum* access to water and food. Mice were euthanised five months after birth by sedation with inhalation of isoflurane followed by cervical dislocation. Immediately after death, whole carcasses were flash-frozen in liquid N_2 and stored at –80 °C. Immediately prior to beginning the decay experiment, carcasses were allowed to thaw to 20 °C over the course of approximately two hours.

All equipment underwent dry heat sterilisation at 160 °C for 12 hours prior to use. For each carcass, 500 g of inert quartz sand (210–297 µg granularity; Sigma–Aldrich), hereafter referred to as “matrix”, was measured into a glass beaker using a mass balance. A 500 mL glass Kilner jar was filled with approximately 200 g of matrix, and a strip of nylon mesh (50–100 µm pore size; Streno), cut to approximately 15 cm × 30 cm with sterile scissors, was laid over the 200 g of matrix within the jar. The carcass was placed in the centre of the nylon strip perpendicular to its length, with the rostral aspect of the body oriented toward the lock of the jar to facilitate discriminating the head region during advanced decay. The remaining 300 g of matrix was poured into the jar to completely cover the carcass. The ends of the nylon strip were exposed above the surface to facilitate removing the carcass during sampling.

Four experimental conditions were created to mimic degrees of waterlogging and oxygenation: wet–oxic (HWHO), wet–hypoxic (HWLO), dry–oxic (LWHO), and dry–hypoxic (LWLO). Three mice (designated replicates A, B and C) were decayed in each of the four conditions at each of six time points – 24 hours (T_1), 72 hours (T_2), one week (T_3), six weeks (T_4), three months (T_5) and six months (T_6) – for a total of 12 mice at each time point. It should be noted that, in an ideal longitudinal experiment, one would sample the brain of a single individual at each time point (i.e., one carcass six times); however, this would necessitate repetitive exhumation and reburial, disturbing both the decaying tissues and the microenvironment, and introduce additional sources of variation. Given every effort was

made to minimise interindividual variation in animal samples, coupled with our triplication of each condition, it was determined that exhuming an individual at every time point (i.e., sampling six carcasses once) was likely a more robust experimental design.

To determine the volume of water appropriate for wet and dry conditions, a test Kilner jar with 500 g of matrix was filled slowly with ultrapure Milli-Q® H_2O (Sigma–Aldrich) until a layer of clear water (> 5 mm) was established above the surface, simulating waterlogged burial contexts. The amount of water used was measured and determined to be 180 mL. Thus, 180 mL of Milli-Q® H_2O was used for wet conditions, and half this volume (90 mL) for dry conditions. Oxic conditions (i.e., with atmospheric exchange and oxygen replenishment) were simulated by leaving the lid of the jar open, allowing air to circulate; hypoxic conditions (i.e., without atmospheric exchange and oxygen replenishment) were simulated by fastening the lid of the jar, and an airtight seal ensured by the use of a silicon gasket. All jars (n = 72) were placed together in a fume hood with controlled temperature (20 °C), ambient laboratory lighting, and air circulation. Water levels were monitored at each time point by comparing the mass of the dry jars with the equivalent wet jars, and the mass difference (ΔM, accounted for by the loss of water) determined. No difference was observed at T_1, T_2, or T_3. At T_4, the ΔM was determined to be 140 g between wet–oxic and wet–hypoxic conditions, and 100 g between dry–oxic and dry–hypoxic conditions; thus, 140 mL of Milli-Q® H_2O was added to each of the remaining replicates in the wet–oxic condition, and 100 mL Milli-Q® H_2O to each of the replicates in the dry–oxic condition. At T_5, the ΔM was determined to be 200 g between wet–oxic and wet–hypoxic conditions, and 100 g between dry–oxic and dry–hypoxic conditions; thus, 200 mL of Milli-Q® H_2O was added to each of the remaining replicates in the wet–oxic condition, and 100 mL Milli-Q® H_2O to replicates in the dry–oxic condition.

Sterile, single-use, disposable equipment (e.g., scalpel blades, petri dishes) were used for sampling wherever possible. All non-disposable surgical equipment (e.g., scissors, spatulae) underwent dry heat sterilisation at 160 °C for 12 hours prior to use, and were treated with 70% ethanol prior to and immediately after each use. At each time point, 12 jars (replicates A, B and C for each of the four experimental conditions) were removed from the fume hood. The carcass was removed from the jar by carefully lifting out the nylon strip. Matrix was loosened to expose the carcass with minimal disturbance. Using large scissors, the carcass was decapitated caudal to the skull (at the level of the cervical vertebrae) and the head placed on a petri dish. Using fine scissors, the skin of the scalp was incised toward the snout and peeled apart and forward to expose the skull, which was cut along the midline. Holding the head steady by the snout, the two halves of the skull were pulled apart with fine tweezers to expose the brain. The brain was removed from the endocranial cavity using a spatula, briefly homogenised by mechanical action, and divided evenly into five samples of approximately 80 mg each. The five sample sets (n = 360) were collected in 0.5 mL Eppendorf Safe-Lock® tubes (Thermo Fisher Scientific), immediately flash-frozen in liquid N_2, and stored at –80 °C.

Proteomics

Data-dependent acquisition

Samples for data-dependent acquisition (DDA; n = 72) were lysed in 100 mM ammonium bicarbonate (ambic; Sigma–Aldrich) with 2% sodium dodecyl sulfate (SDS; Sigma–Aldrich) and 1X Halt™ protease inhibitor cocktail (Thermo Fisher Scientific), using a Precellys® 24 Touch homogeniser (Bertin Technologies) for 12 cycles of 15 sec at 5,500 rpm, followed by one cycle of 30 sec at 5,500 rpm. Samples were placed on ice between cycles to reduce heating. Protein concentration was estimated using bicinchoninic acid (BCA) assay with bovine serum albumin (BSA) standards (Table S24). 2.5 µg of protein from each sample was diluted to a total volume of 40 µL using 50 mM ambic, and added to a 96-well plate. Cysteine reduction was performed at 60 °C and 1,000 rpm for 10 min with 4 mM dithiothreitol (DTT) solution. Alkylation was performed in the dark at 20 °C for 30 min with 10 mM iodoacetamide (IAA). Each sample was combined with 40 ng SP3 beads (1:1 hydrophobic:hydrophilic) [35] and acetonitrile (ACN) added to a final concentration of 80%. The solution was gently mixed by pipetting and incubated at 20 °C and 1,000 rpm for 30 min. The plate was placed on a magnetic rack and the beads allowed to settle for 5 min, before three washes with 100% ACN. Beads were resuspended in 100 mM ambic, and the plate sonicated for 2 min. Digestion was performed with 0.2 µg/µL trypsin (MS-grade, Promega) at 37 °C and 1,000 rpm overnight. Each sample was acidified with trifluoroacetic acid (TFA) to 1%, and incubated at 37 °C without shaking for 30 min,

followed by 30 min on ice. Eluted peptides were dried by vacuum centrifugation for storage at –20 °C prior to analysis.

Liquid chromatography–tandem mass spectrometry (LC–MS/MS) was performed on a Dionex UltiMate™ 3000 Rapid Separation LC system coupled to a Q Exactive™ Plus Hybrid Quadrupole-Orbitrap™ mass spectrometer (both Thermo Fisher Scientific). To determine injection volumes (target base peak intensity [BPI]: 1–2e8), survey runs of 15 min gradient (30 min program) were performed on each sample; based on the BPI obtained, the final injection volume of samples varied between 4–15 µL. The sample was loaded onto an Acclaim™ PepMap™ 100 C18 HPLC cartridge (5mm × 300 µm) using partial loop injection for 7 min at a flow rate of 12 µL/min with 0.1% (v/v) TFA, and resolved on an EASY-Spray™ PepMap™ C18 HPLC column (500 mm × 75 µm, both Thermo Fisher Scientific) using a 90 min linear gradient of 96.2% buffer A (0.1% formic acid [FA]) and 3.8% buffer B (80% ACN, 19.9% H_2O, 0.1% FA), to 50% A to 50% B at 300 nL/min flow rate. In DDA mode, MS1 spectra were acquired in the Orbitrap™ at 70K resolution between 300–2000 *m/z*, with an automatic gain control target of 1e6 ions, and a maximum injection time of 250 ms. Precursor ions associated with the five most abundant peaks were fragmented using high energy collisional dissociation with 30% normalised collision energy. MS2 spectra were acquired at 35K resolution with a quad isolation window of 2.0 Da and a maximum injection time of 300 ms, and a 20 sec dynamic exclusion window to avoid repeated selection of precursors for MS2.

Data-independent acquisition

Samples for data-independent acquisition (DIA; *n* = 72) were lysed in Pierce® RIPA buffer (Thermo Fisher Scientific), using a Precellys® 24 Touch homogeniser (Bertin Technologies) for three cycles of 45 sec at 5,000 rpm. Samples were placed on ice between cycles to reduce heating. Protein concentration was estimated using BCA assay with BSA standards (Table S25). 10 µg of protein from each sample was diluted to a total volume of 15 µL using RIPA buffer, and added to a 96-well plate. Cysteine reduction was performed at 60 °C and 1,000 rpm for 10 min with 5 mM DTT solution. Alkylation was performed in the dark at 20 °C for 30 min with 20 mM IAA. Each sample was combined with 2 µL SP3 beads (1:1 hydrophobic:hydrophilic) and ACN added to a final concentration of 70%. The solution was gently mixed by pipetting and incubated at 20 °C and 1,000 rpm for 18 min. The plate was placed on a magnetic rack and the beads allowed to settle for 5 min, before two washes with 70% ethanol and two washes with 100% ACN. Beads were resuspended in 50 mM ambic, and digestion performed with 8 ng/µL trypsin (MS-grade, Promega) at 37 °C and 1,000 rpm overnight. Each sample was acidified with TFA to 1%. Eluted peptides were dried by vacuum centrifugation for storage at –20 °C prior to analysis.

LC–MS/MS was performed on a TimsTOF Pro mass spectrometer (Bruker). Peptides were reconstituted in 0.1% FA and an estimated 200 ng loaded onto Evotips (Evosep Biosystems) according to the manufacturer's instructions, then chromatographically separated using an Evosep One (Evosep Biosystems) using the 60 spd standard method. Peptides were eluted onto the TimsTOF Pro, which was operated in diaPASEF mode using eight diaPASEF scans per TIMS–MS scan. The ion mobility range ($1/K_0$) was set to 0.64–1.41 V·s/cm^2. Each mass window isolated was 25 *m/z* wide, ranging from 475–1000 *m/z* with an ion mobility-dependent collision energy that increased linearly from 20 eV to 59 eV between 0.6–1.6 V·s/cm^2. All LC–MS/MS data have been deposited to the ProteomeXchange Consortium *via* the PRIDE [36] partner repository with the dataset identifier, PXD074190.

Data analysis

Protein identification

Both DDA and DIA raw data files (*n* = 144) were searched against the *Mus musculus* brain proteome retrieved from Korovesi *et al.* [37]. Mapping one-protein-per-gene identified in normal mouse brain regions and having removed duplicates (i.e., those expressed in more than one region), 6,252 proteins remained corresponding to the mouse brain proteome, which were downloaded as a FASTA file. In all cases, data were searched against this FASTA with semi-specific tryptic digestion and the fixed modification of carbamidomethylation of Cys, and the following variable post-translational modifications (PTMs): oxidation of Met and Pro; deamidation of Arg, Gln, and Asn; and protein N-terminal acetylation.

A search of DDA raw data files (*n* = 72) based on *in silico* FASTA digest with label-free quantification (LFQ) and match-between-runs (MBR) in MaxQuant (v.2.4.3.0) [38] yielded the fewest unique proteins identified at 1%

false discovery rate (FDR), but the most information in terms of protein modification. Search criteria included a first, fully tryptic search with up to three missed cleavages allowed [39] and a peptide tolerance of 20 ppm, followed by a main, semi-specific tryptic search and a peptide tolerance of 4.5 ppm, followed by a final dependent peptide search for covalently-modified peptides. All subsequent analyses were performed on the DDA dataset using the R programming language (v.2026.01.0): global peptide and protein lists were removed of potential contaminants, reversed identifications, and identifications with < 2 unique peptides, then filtered for q-value and posterior error probability (PEP) ≤ 0.01. Modified specific and dependent peptide lists were removed of unannotated modifications and those corresponding to < 2 unique peptides, then filtered for q-value and PEP ≤ 0.05.

A library-free search of all DIA raw data files (n = 72) was performed with DIA-NN (v.2.0) [40]. Search criteria included a semi-specific tryptic search with one missed cleavage and up to three variable PTMs allowed, and both a mass and MS1 accuracy of 15 ppm. All subsequent analyses were performed on the DIA dataset using R. Precursors were filtered using the following parameters: precursor q-value ≤ 0.01, protein q-value ≤ 0.01, and protein- and gene-group q-values ≤ 1. Peptides and protein groups were filtered with the same parameters, and removed of potential contaminants, reversed identifications, and identifications with < 2 unique peptides. Data were preprocessed using *DIAgui* (v.1.4.2) [41], which integrates the *iq* package (v.1.9.12) [42] to perform proteome-wide LFQ *via* delayed normalisation and maximal peptide ratio extraction (*aka*. MaxLFQ) [43], and the *imputeLCMD* package (v.2.1.0) for missing value imputation *via* quantile regression of left-censored data (QRILC) [44,45]. Precursors were filtered using the following parameters: precursor q-value ≤ 0.01, protein q-value ≤ 0.01, and protein- and gene-group q-values ≤ 1. Peptides and protein groups were filtered with the same parameters, and removed of potential contaminants, reversed identifications, and identifications with < 2 unique peptides. Protein intensities were summarised as intensity-based absolute quantification (iBAQ) values at the protein-group level; calculated as the sum of peptide intensities divided by the number of theoretically-observable tryptic peptides per protein [46]. This length-normalised metric provides an approximate proxy for molar protein abundance and reduces the dependence of raw LFQ intensities on protein size. Given the aim of the DIA analysis was to characterise shifts in the composition of the brain proteome and to compare the relative representation of protein classes across PMIs and burial environments, $\log_2$-transformed iBAQ values were used for ordination, differential expression analysis (DEA), and downstream clustering, while peptide-level analyses of decay trajectories were performed separately on peptide intensities. Log_2 transformation was used to stabilise variance and facilitate interpretation of abundance differences and decay slopes in terms of fold change. Batch effects associated with MS run were corrected using the *sva* package (v.3.2.1) [47] *via* the ComBat algorithm. All subsequent analyses were performed on the DIA dataset, unless otherwise stated.

Ordination and differential expression analysis

PCA and UMAP were conducted on the $\log_2$-transformed iBAQ intensity matrix with the *factoextra* (v.3.1.2) [48] and *uwot* packages (v.0.2.3) [49], respectively. Prior to analysis, protein abundances were mean-centred and scaled to unit variance (i.e., z-scored per protein across samples). PCA employed a covariance-based approach, while UMAP was implemented with ten nearest neighbours, a minimum distance of 0.05, and a cosine similarity metric, using a fixed random seed to ensure reproducibility. Based on the results of dimension reduction, PMI was divided into two periods corresponding to "early" (< 6 weeks) and "late" (≥ 6 weeks) decay. DEA was performed using the *limma* package (v.3.2.1) [50]: iBAQ data were modelled using a design matrix with additive main effects for experimental condition and PMI. Empirical-Bayes moderated statistics were computed, and proteins considered differentially expressed for PMI if FDR-adjusted p < 0.05 and $\log_2$-fold change ($\log_2$FC) > 1 (coefficient: late PMI). Significant proteins were standardised by per-protein z-scoring across samples, and clustered by k-means: the elbow and average silhouette width were inspected for k = 1–10, and k = 2 chosen for downstream analysis (Fig S17). Gene Ontology (GO) enrichment of resulting clusters was performed using *clusterProfiler* (v.3.10.1) [51] and visualised using *enrichplot* (v.1.10.1) [52]. A composite heatmap of z-scored modelled expression was generated with *ComplexHeatmap* (v.3.21) [53], using k-means cluster assignment and hierarchical clustering to order rows and columns.

Peptide preservation modelling and classification

Peptide preservation was assessed on the basis of per-peptide decay trajectories, computed from log_2-transformed, batch effect-corrected peptide intensities. Each peptide's time of maximum intensity (t_{max}) was identified and used to define early-peaking ($t_{max} \leq 1$ week) and late-peaking ($t_{max} \geq 6$ weeks) groups as proxies for "labile" (relatively decay-prone) and "recalcitrant" (relatively decay-resistant) behaviour. Peptide-specific decay rates were then estimated by fitting ordinary least squares regressions of log_2-intensity on PMI, from which slope estimates, standard errors (SE), *p*-values, and residual degrees of freedom were extracted. To ensure robust trend estimation, peptides with SEs exceeding the 90th percentile and/or detection frequencies below it were excluded; given no peptides detected in the wet–oxic condition met these criteria, these samples were omitted from the analysis. To determine a target positive predictive value (PPV) for early- and late-peaking calls, candidate slope thresholds (τ; $n = 200$) spanning the observed slope range were evaluated by computing precision and recall at each threshold, and precision–recall (Pareto) frontiers generated. Two per-condition classification thresholds were derived based on (i) weighted-mean and (ii) median slopes: the sensitivity and specificity of each were compared *via* balanced accuracy, and an additive margin δ was grid-searched ($\delta \in [0,0.020]$) with bootstrapping resamples ($n = 200$) to achieve a target PPV for recalcitrant calls ≥ 0.80. Final classification employed an optimised weighted mean-based threshold to designate peptides as recalcitrant (slope $\geq \tau_{mean} + \delta$), labile (slope $< \tau_{mean} - \delta$), or putative (otherwise), with bootstrap resamples ($n = 1{,}000$) yielding 95% confidence intervals (CIs) for PPV and negative predictive value (NPV). Calibration was assessed by decile binning of observed slopes and predicted probabilities, and by fitting a logistic regression with Spiegelhalter's *Z*-statistic, Brier score, and ROC AUC goodness-of-fit tests quantifying performance. Smoothed-mean decay trajectories were fit using a generalised additive model with the package *mgcv* (v.1.9.3) [54].

Biochemical correlates of peptide preservation

For each experimental condition, over- and under-representation of protein classes associated with peptides of each preservation category (i.e., the difference between the number of observed *vs.* expected) was investigated using a Fisher's exact test and Bonferroni correction for multiple comparisons with the Protein ANalysis THrough Evolutionary Relationships (PANTHER) classification system (v.19.0) [55]. GRand AVerage of hydropathY (GRAVY) scores were calculated in R for all peptides using the Kyte–Doolittle hydrophobicity scale [56]. For each experimental condition the distribution of GRAVY scores across preservation categories was assessed non-parametrically: a Kruskal–Wallis test with eta-squared effect sizes (η^2H) was performed to detect any overall differences, and η^2H values interpreted using conventional thresholds ($\eta^2H < 0.01$ negligible; 0.01–0.06 small; 0.06–0.14 moderate; ≥ 0.14 strong) [57]. Where the omnibus test indicated differences, pairwise *post-hoc* comparisons were conducted using Dunn's test with Holm (family-wise) *p*-value adjustment, and a rank biserial correlation (*r*) for each pairwise contrast reported ($|Z|/\sqrt{n}$). Isoelectric points (pI) were estimated for all peptides using the *Peptides* package (v.2.4.6) [58] in R. For each sequence, theoretical pI values were first computed under nine standard pK scales (Bjellqvist, EMBOSS, Murray, Sillero, Solomon, Stryer, Lehninger, Dawson, and Rodwell) and averaged to obtain a consensus experimental estimate. These preliminary estimates were refined by incorporating machine-learning predictions from the Isoelectric Point Calculator (IPC) 2.0 [59], to produce a final predicted pI for each peptide. For each experimental condition, Kruskal–Wallis with pairwise Dunn tests (Holm-adjusted) were used to assess category-wise differences in pI.

Compositional and structural correlates of peptide preservation

Sequence coverage and amino acid composition were computed in R. Groupwise differences in coverage and fragmentation were tested non-parametrically with Kruskal–Wallis and pairwise Dunn tests (Holm-adjusted). Effect magnitudes were reported as Cliff's delta and interpreted using conventional thresholds ($\delta < 0.147$ negligible; 0.147–0.330 small; 0.330–0.474 moderate; ≥ 0.474 strong) [60]. Empirical cumulative distribution functions (ECDFs) were plotted with 95% bootstrap CIs ($n = 1{,}000$ resamples, grid 0–100% in 0.5% steps) and median CIs ($n = 2{,}000$ resamples); bootstrap seeds were fixed for reproducibility. Amino acid composition was compared at two scales: (i) global within-peptide relative frequencies were contrasted between preservation categories using empirical-Bayes-moderated linear models (primary contrast: recalcitrant *vs.* labile) with Benjamini–Hochberg adjustment, and (ii) terminal positional composition (three N-terminal and three C-terminal residues) assessed by one-hot encoding of terminal residues (excluding amino acids with < 5 observations at a given position) followed by moderated contrasts.

Secondary structure was assessed using a model-based approach. Prediction files for the *Mus musculus* proteome (n = 21,452) were downloaded from AlphaFold (accessed 07.10.2025) [61], and processed in R using the *bio3d* package [62]. Per-atom B-factors were averaged to yield per-residue pLDDT estimates, and backbone torsions computed. Per-residue φ/ψ were converted to conservative secondary-structure calls (α-helix, β-sheet, or coil) by angular windows on φ/ψ. For each peptide the subset of per-residue rows spanning its mapped coordinates was extracted and used to compute fractional α-helix, β-sheet, and coil composition; dominant structural class (i.e., largest fractional component); peptide length, and mean pLDDT. Association between structure class and preservation type was tested by Pearson's χ^2 with standardised residuals to identify over- and under-represented combinations. Binomial logistic regression modelled peptide recalcitrance (binary outcome) as a function of helix and coil fractions, mean pLDDT, and peptide length, with the β-sheet fraction as the implicit reference (based on the results of dominant structure frequencies). The model was repeated after excluding peptides with low (< 70) and very low (< 50) mean pLDDT scores to confirm stability against low-confidence regions. Finally, helix, coil and sheet fractions were all recalculated after weighting by per-peptide structural confidence (fraction × [pLDDT/100]): group means were summarised by preservation category, and global differences assessed using Kruskal–Wallis tests with pairwise Dunn tests (Holm-adjusted). Both (i) excluding low confidence peptides and (ii) reweighting by confidence yielded distributions and between-category differences that were indistinguishable from the unweighted analysis. Intrinsic disorder was investigated using AIUPred [63]: disorder scores were parsed and joined to peptide coordinates in R, and tested non-parametrically with Kruskal–Wallis and pairwise Dunn tests (Holm-adjusted) for (i) mean IUPred score (reflecting the overall strength of disorder tendencies), and (ii) the fraction of residues exceeding the disorder threshold (≥ 0.5; reflecting the extent of confidently disordered regions).

Functional feature analyses

Peptide features were annotated in R by retrieving the UniProtKB JSON record for each unique protein accession (accessed 14.10.2025) [64], parsing these into numeric feature ranges, and overlapping them with peptide intervals to identify intersecting annotated regions. Peptide–feature association testing followed a three-tier hierarchical workflow (UniProt section → feature type → descriptor). Secondary-structure annotations were removed before testing (n = 288 annotated peptides), given these were analysed previously. For each section, and then for each type within sections that evidenced significant overall associations, contingency testing was performed using Pearson's χ^2 tests (n = 215,088 peptide–UniProt feature observations corresponding to 94,296 unique peptides) and overall effect size quantified with Cramér's *V*. To reduce underpowered descriptor tests, a conservative minimum-occurrence threshold was computed from the observed category proportions using Cohen's w (0.3) with high target power (0.95), inflated for multiple testing and converted into expected positive counts under several presence rate scenarios; descriptors were tested only if they met peptide- and protein-count minima (≥ 2 unique peptides/proteins). Benjamini–Hochberg adjustment was applied globally and, for significant descriptors, pairwise comparisons with odds ratios (ORs) and 95% CIs reported.

Post-translational modification analysis

PTM analysis was performed in R on the results of MaxQuant's dependent peptide search of the DDA data. Identifications were removed where (i) the PEP > 0.05, (ii) the score < 50, or where the modification type was (iii) unclassified or (iv) unmodified. Ratios of modified to base intensities were $\log_2$-transformed. To screen for between-condition differences in modification frequency, a 4 × 2 contingency table (four burial conditions × modification present/absent) was constructed per modification for each PMI group, and tested with Pearson's χ^2 when expected counts ≥ 5 or with Fisher's exact test otherwise; p-values were adjusted within each PMI group by the Benjamini–Hochberg method. Modifications with (i) ≥ 10 observations total, (ii) ≥ 5 observations per PMI group, and (iii) an adjusted p-value ≤ .05 were designated "target modifications". For each target, within-condition early *vs.* late PMI comparisons were performed using binomial generalised linear models, with problematic fits (e.g., non-finite SEs) refit using Firth's penalised logistic regression in the *logistf* package (v.1.26.1) [65]. Model coefficients, SEs, ORs, and 95% CIs were reported, and p-values corrected within each condition by Benjamini–Hochberg. PTMs were validated by inspecting per-peptide site-localisation probabilities to ensure that each reported modification was chemically consistent with the pool of potentially affected sites; the most frequently affected residues were computed as the percentage of that pool with localisation probabilities (P_{loc}) ≥ 0.75.

Figures & Tables

Table S1. Proteins recovered by experimental condition and time point | Summary statistics for numbers of proteins at 1% FDR and *q*-value ≤ .01 recovered in the DIA dataset (*n* = 192,244), grouped by time point and experimental condition.

Time point	Total	Mean	Standard dev.	Median	Min. value	Max. value
**24 hours**	43,286	3,607	78	3,632	3,435	3,695
**72 hours**	42,280	3,523	132	3,549	3,233	3,681
**1 week**	41,044	3,420	132	3,459	3,033	3,522
**6 weeks**	27,056	2,255	552	2,318	1,157	3,000
**3 months**	21,667	1,806	585	1,945	441	2,800
**6 months**	16,911	1,409	1,007	1,936	0	2,712
**Condition**	**Total**	**Mean**	**Standard dev.**	**Median**	**Min. value**	**Max. value**
**HWHO**	40,150	2,231	1,411	2,973	0	3,634
**HWLO**	50,479	2,804	761	3,010	1,762	3,620
**LWHO**	48,707	2,706	1,026	3,082	508	3,678
**LWLO**	52,908	2,939	693	3,117	1,970	3,695

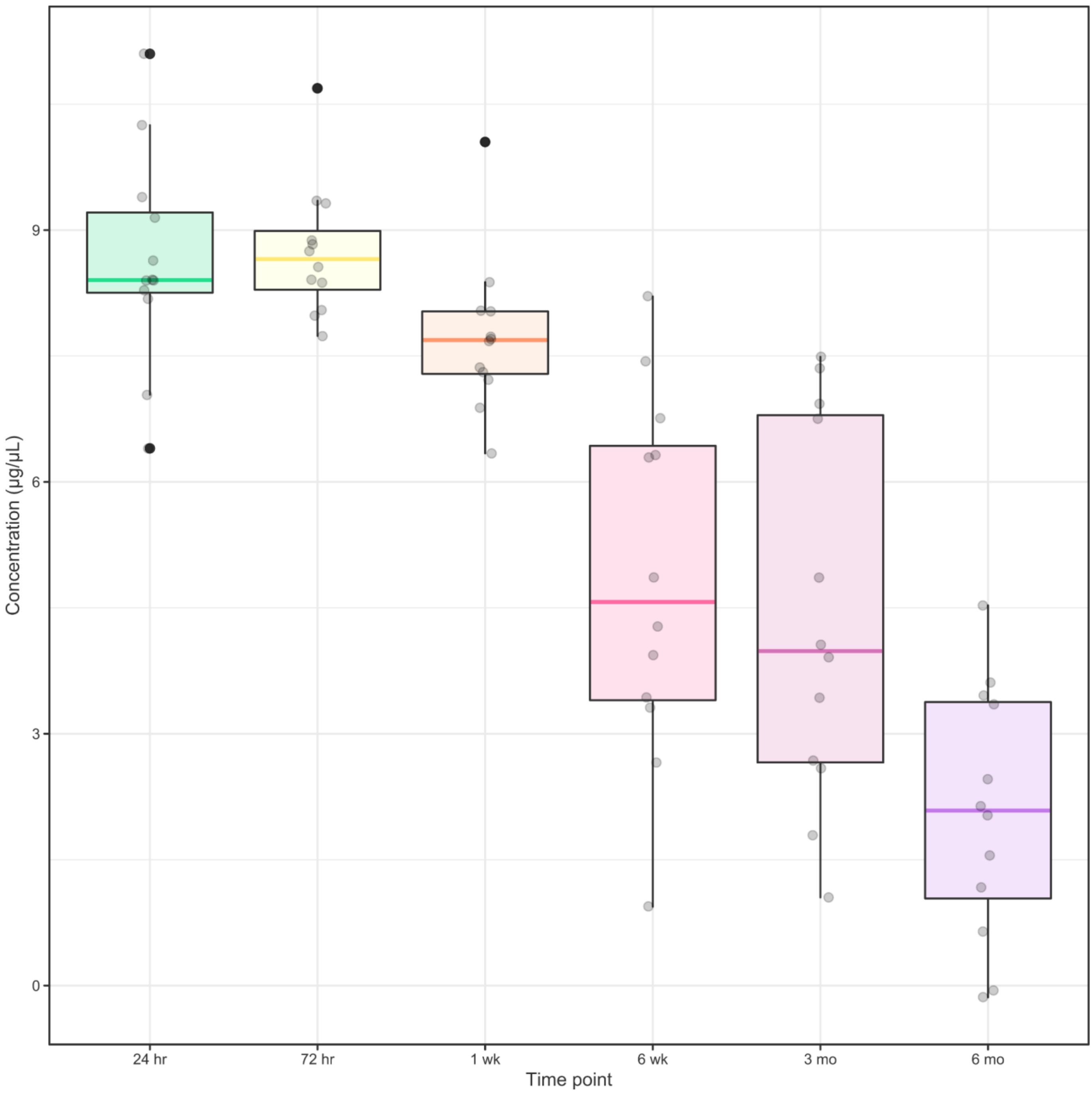

Fig S1. Protein concentration during decomposition | Jittered box plots of mean protein concentrations (estimated by BCA assay) recovered from samples (n = 72) at six time points. Solid black dots represent statistical outliers.

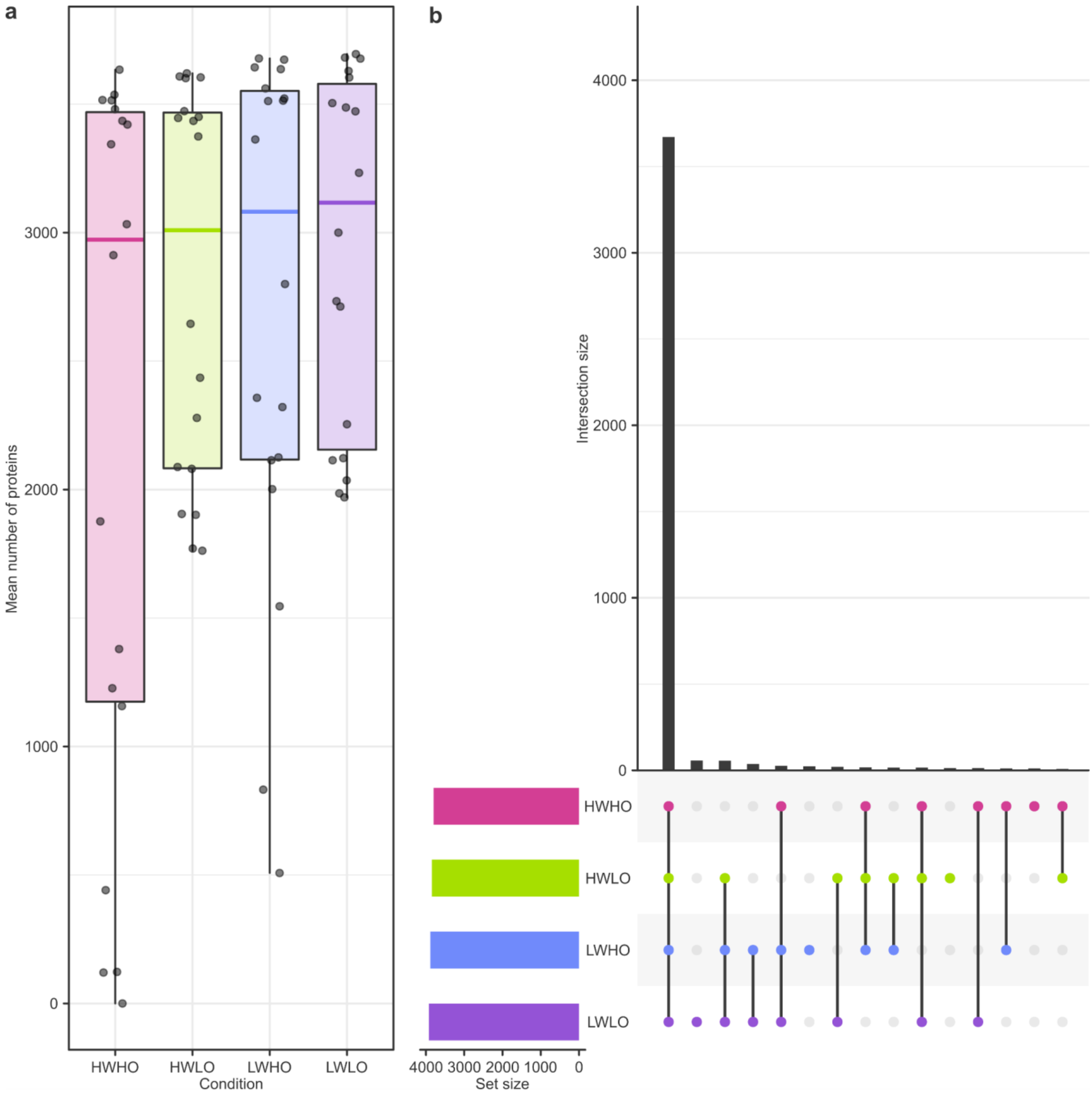

Fig S2. Proteomic profiles by burial environment | (a) Jittered box plot of mean number of proteins at 1% FDR and *q*-value ≤ .01 recovered in each experimental condition, and **(b)** upset plot of the number of unique proteins at 1% FDR and *q*-value ≤ .01 recovered in each experimental condition. Horizontal bars indicate the total peptides per individual condition; connected dots denote the condition(s) contributing to each intersection, and vertical bars indicate intersection sizes.

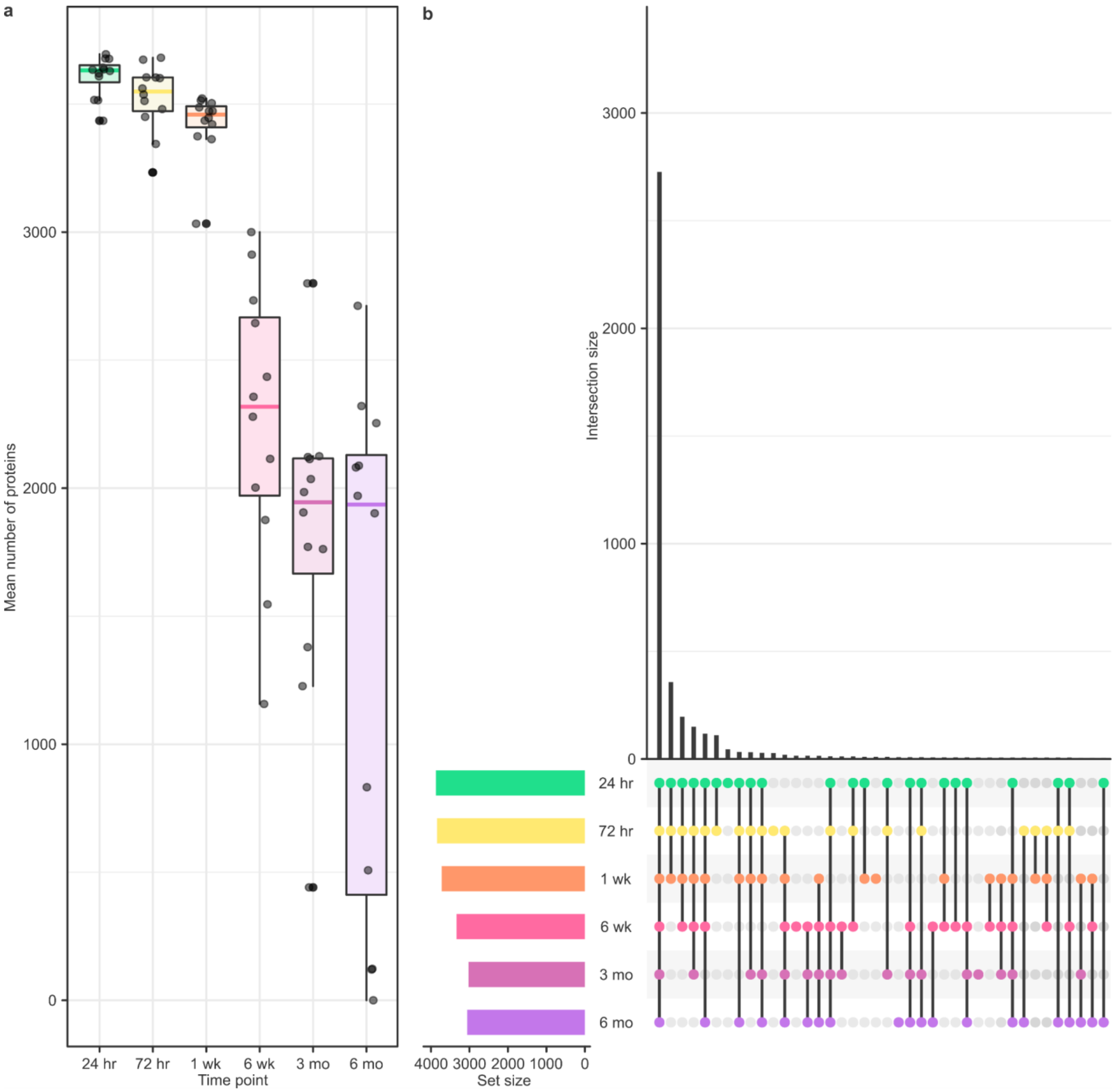

Fig S3. Proteomic profiles by time point | (a) Jittered box plot of mean number of proteins at 1% FDR and *q*-value ≤ .01 recovered at each time point (solid black points represent statistical outliers), and **(b)** upset plot of the number of unique proteins at 1% FDR and *q*-value ≤ .01 recovered at each time point. Horizontal bars indicate the total peptides per individual time point; connected dots denote the time point(s) contributing to each intersection, and vertical bars indicate intersection sizes.

Table S2. Protein abundance at different time points | Results of a linear mixed effects model (random intercept by condition) with type-III ANOVA (Satterthwaite method) and Bonferroni-adjusted pairwise contrasts (n = 72) for the number of proteins identified at 1% FDR and *q*-value ≤ .01 across time points. Cells containing *p*-values are coloured based on the following significance codes: | < .0001 | ≤ .001 | ≤ .01 | ≤ .05 | non-significant |

Comparison	Estimate (E)	Standard error (SE)	t-Ratio	p-Value
24 hours – 72 hours	83.83333	189.6147	0.4421248	1.00×10^{0}
24 hours – 1 week	186.83333	189.6147	0.9853317	1.00×10^{0}
24 hours – 6 weeks	1352.50000	189.6147	7.1328876	1.76×10^{-8}
24 hours – 3 months	1801.58333	189.6147	9.5012876	1.33×10^{-12}
24 hours – 6 months	2197.91667	189.6147	11.5914917	4.32×10^{-16}

72 hours – 1 week	103.00000	189.6147	0.5432070	1.00×10^{0}
72 hours – 6 weeks	1268.66667	189.6147	6.6907628	1.03×10^{-7}
72 hours – 3 months	1717.75000	189.6147	9.0591628	7.67×10^{-12}
72 hours – 6 months	2114.08333	189.6147	11.1493670	2.27×10^{-15}
1 week – 6 weeks	1165.66667	189.6147	6.1475559	8.92×10^{-7}
1 week – 3 months	1614.75000	189.6147	8.5159558	6.73×10^{-11}
1 week – 6 months	2011.08333	189.6147	10.6061600	1.80×10^{-14}
6 weeks – 3 months	449.08333	189.6147	2.3684000	3.14×10^{-1}
6 weeks – 6 months	845.41667	189.6147	4.4586041	5.20×10^{-4}
3 months – 6 months	396.33333	189.6147	2.0902042	6.10×10^{-1}

Table S3. Protein abundance in different burial environments | Results of a linear mixed effects model (random intercept by time point) with type-III ANOVA (Satterthwaite method) and Bonferroni-adjusted pairwise contrasts (n = 72) for the number of proteins identified at 1% FDR and q-value ≤ .01 across experimental conditions. Cells containing p-values are coloured based on the following significance codes: | < .0001 | ≤ .001 | ≤ .01 | ≤ .05 | non-significant |

**Comparison**	**Estimate (*E*)**	**Standard error (SE)**	***t*-Ratio**	***p*-Value**
HWHO – HWLO	–573.83333	154.8197	–3.7064616	2.67×10^{-3}
HWHO – LWHO	–475.38889	154.8197	–3.0705966	1.89×10^{-2}
HWHO – LWLO	–708.77778	154.8197	–4.5780848	1.36×10^{-4}
HWLO – LWHO	98.44444	154.8197	0.6358650	1.00×10^{0}
HWLO – LWLO	–134.94444	154.8197	–0.8716231	1.00×10^{0}
LWHO – LWLO	–233.38889	154.8197	–1.5074882	8.20×10^{-1}

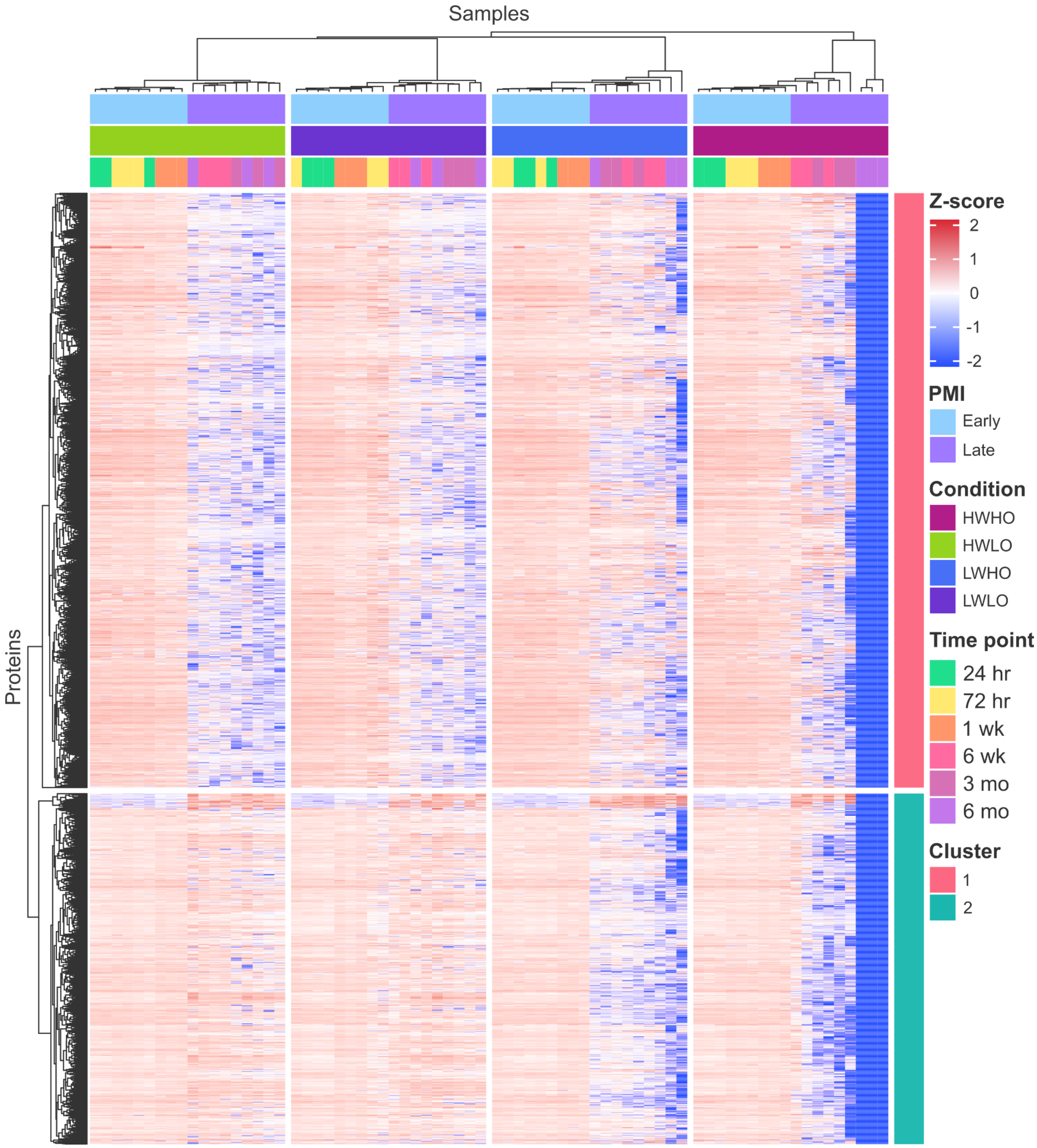

Fig S4. Complex heatmap of scaled protein iBAQ data with integrated linear modelling, *k*-means and hierarchical clustering of proteins globally | Complex heatmap of scaled protein iBAQ data with integrated linear modelling, *k*-means and hierarchical clustering. iBAQ data were log_2-transformed and missing values imputed using QRILC, and fit to a linear model including PMI (main effect) and burial condition (covariate only) to identify proteins with a consistent late-*vs*.-early change in protein abundance across all conditions. Proteins significantly differentially abundant at late PMI relative to early PMI (n = 2,998) were *z*-score normalised and partitioned into two groups by *k*-means clustering. Within each protein cluster, hierarchical clustering using Manhattan distance with complete linkage was applied to resolve differential abundance trends. Columns represent all samples (n = 72), split by the four burial conditions and clustered using Manhattan distance with complete linkage.

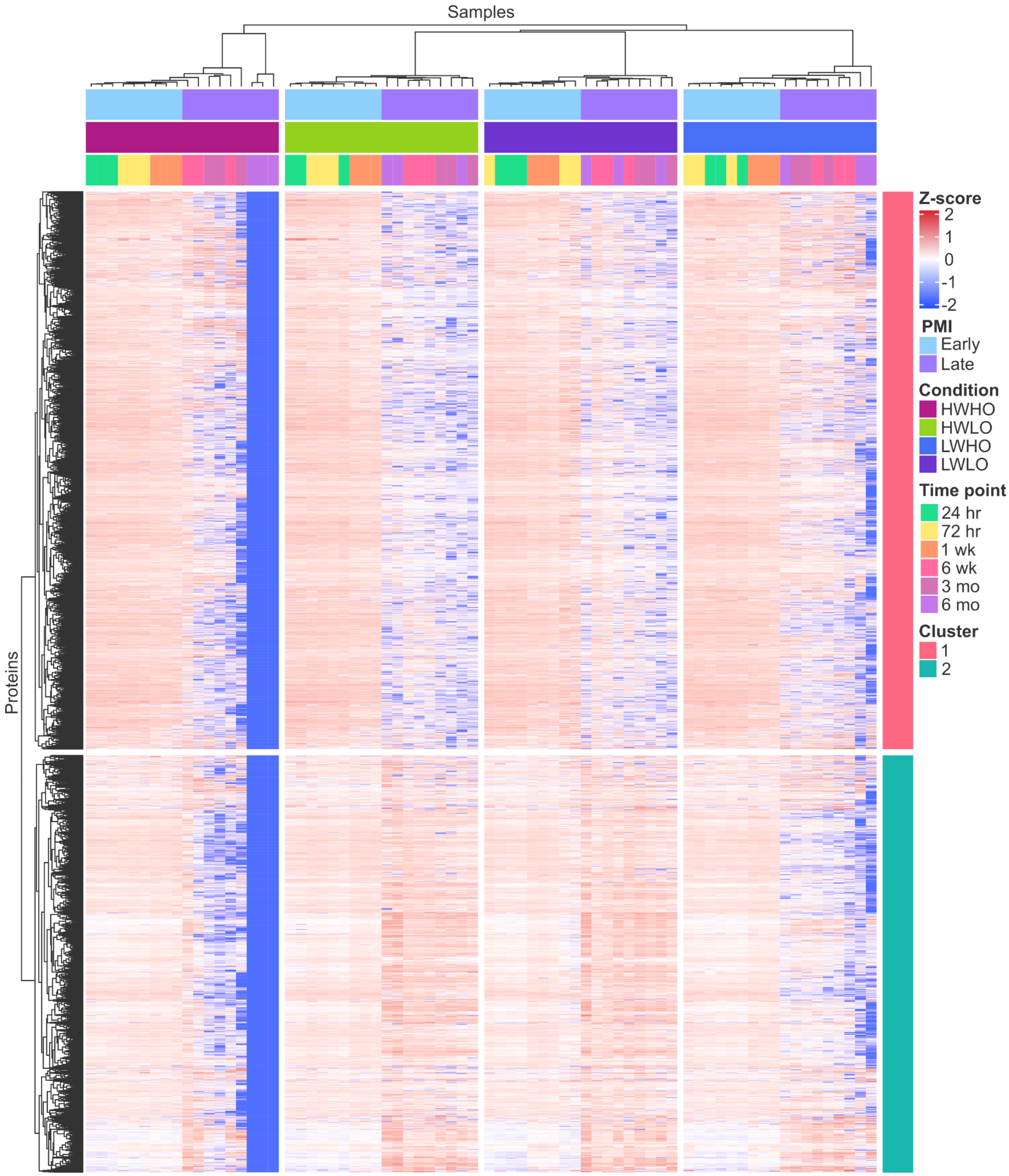

Fig S5. Complex heatmap of scaled protein iBAQ data with integrated linear modelling, *k*-means and hierarchical clustering of proteins in wet–oxic conditions | iBAQ data were log_2-transformed and missing values imputed using QRILC, and fit to a linear model with a condition–PMI interaction to identify proteins with a consistent late-*vs*.-early change in protein abundance in the wet–oxic condition. Proteins significantly differentially abundant at late PMI relative to early PMI (n = 3,791) were z-score normalised, and partitioned into two groups by *k*-means clustering. Within each protein cluster, hierarchical clustering using Manhattan distance with complete linkage was applied to better resolve differential abundance trends. Columns represent samples (n = 72), split by the four experimental conditions and clustered using Manhattan distance with complete linkage.

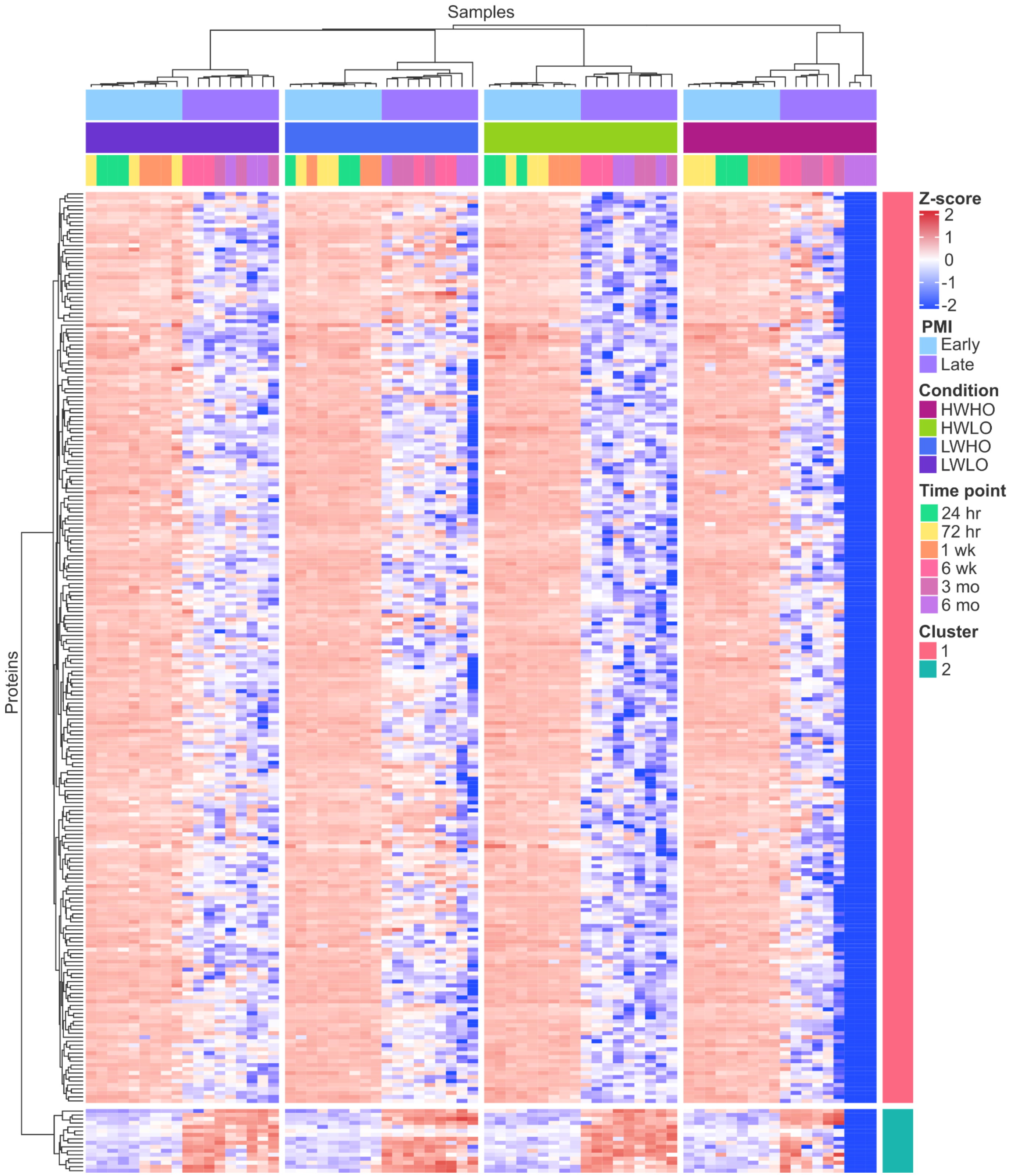

Fig S6. Complex heatmap of scaled protein iBAQ data with integrated linear modelling, *k*-means and hierarchical clustering of proteins in wet–hypoxic conditions | iBAQ data were $\log_2$-transformed and missing values imputed using QRILC, and fit to a linear model with a condition–PMI interaction to identify proteins with a consistent late-*vs.*-early change in protein abundance in the wet–hypoxic condition. Proteins significantly differentially expressed at late PMI relative to early PMI (n = 245) were z-score normalised, and partitioned into two groups by k-means clustering. Within each protein cluster, hierarchical clustering using Manhattan distance with complete linkage was applied to better resolve differential abundance trends. Columns represent all samples (n = 72), split by the four experimental conditions and clustered using Manhattan distance with complete linkage.

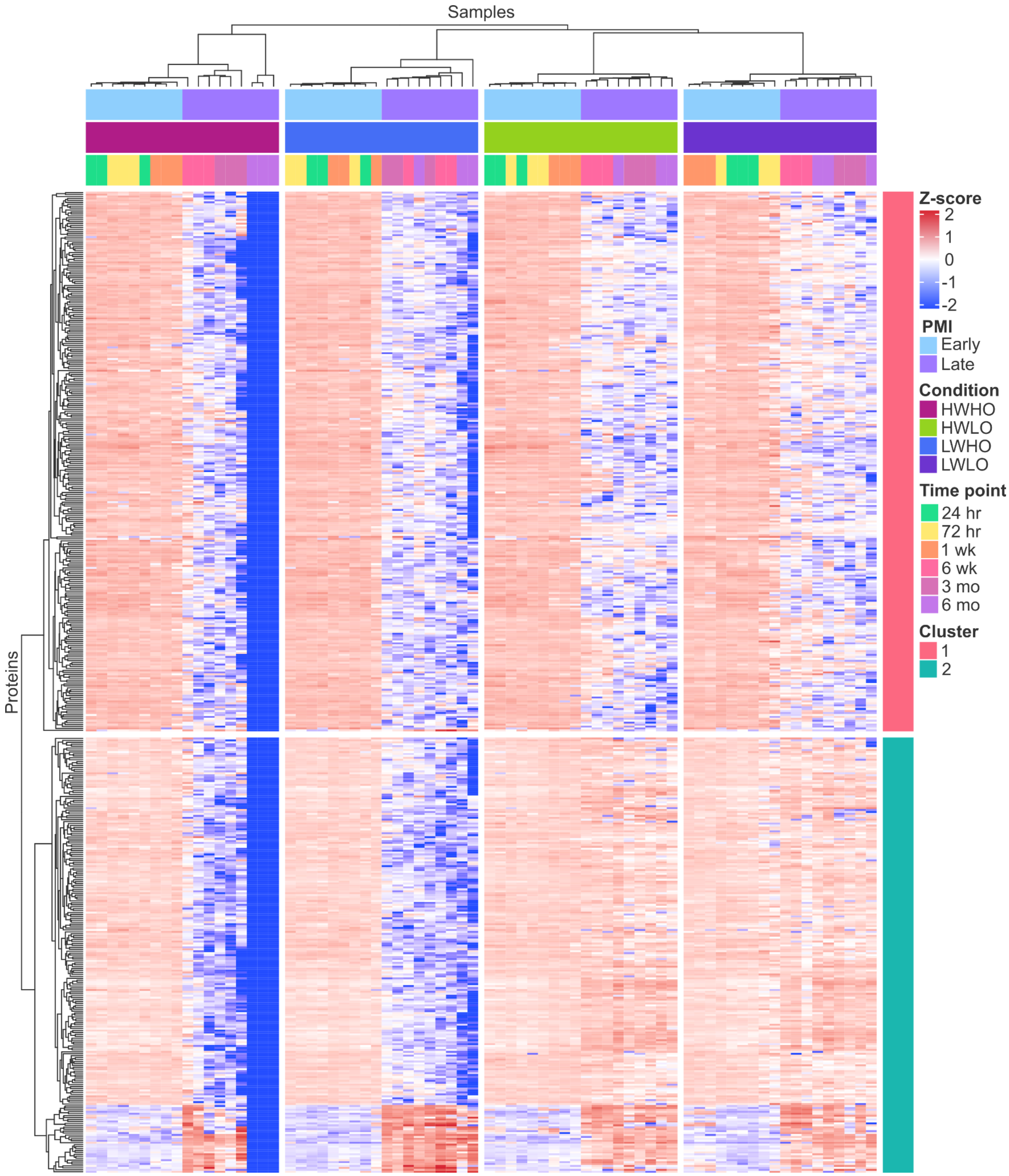

Fig S7. Complex heatmap of scaled protein iBAQ data with integrated linear modelling, *k*-means and hierarchical clustering of proteins in dry–oxic conditions | iBAQ data were log_2-transformed and missing values imputed using QRILC, and fit to a linear model with a condition–PMI interaction to identify proteins with a consistent late-*vs.*-early change in protein abundance in the dry–oxic condition. Proteins significantly differentially abundant at late PMI relative to early PMI (n = 245) were z-score normalised, and partitioned into two groups by k-means clustering. Within each protein cluster, hierarchical clustering using Manhattan distance with complete linkage was applied to better resolve differential abundance trends. Columns represent all samples (n = 72), split by the four experimental conditions and clustered using Manhattan distance with complete linkage.

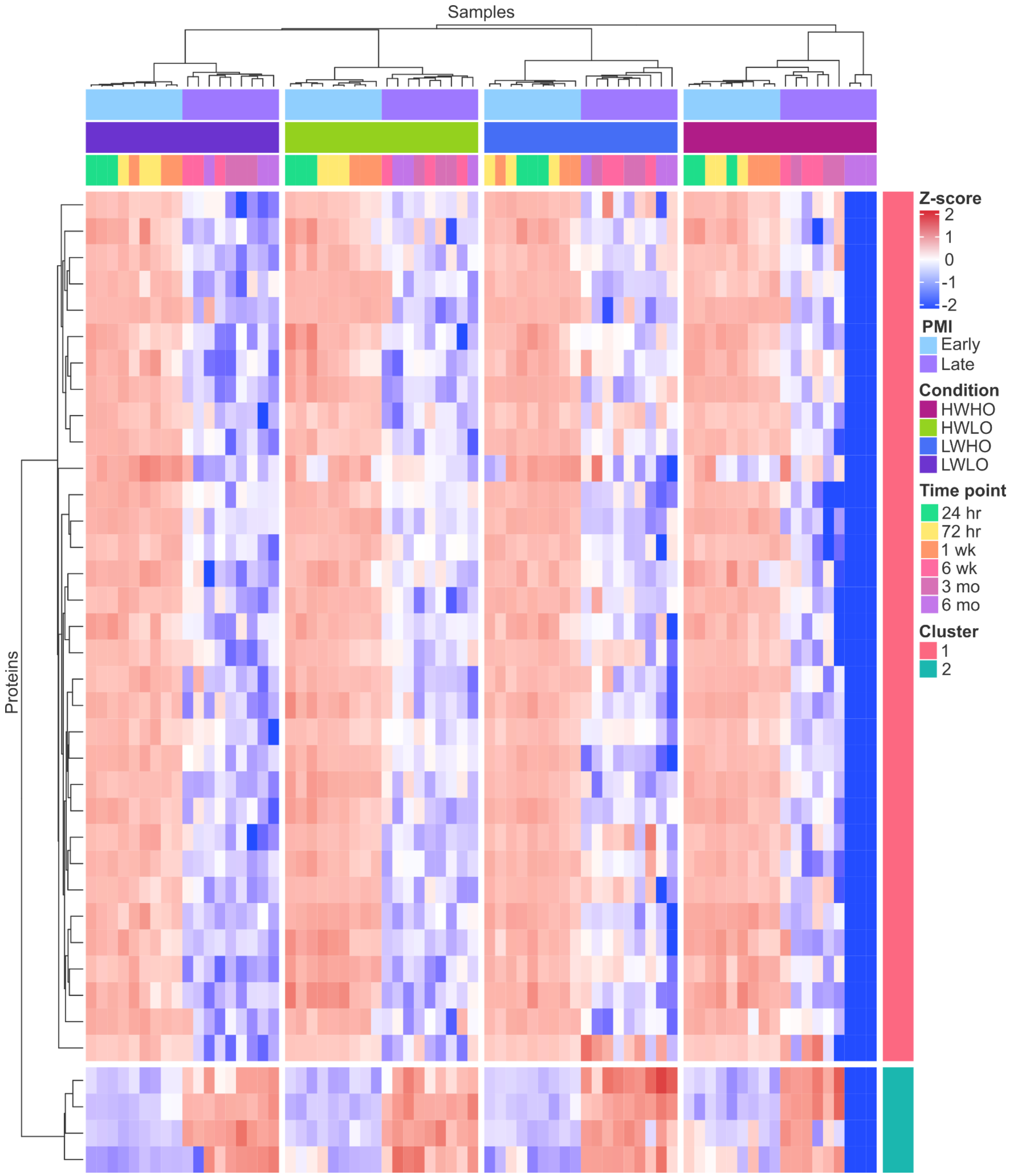

Fig S8. Complex heatmap of scaled protein iBAQ data with integrated linear modelling, *k*-means and hierarchical clustering of proteins in dry–hypoxic conditions | iBAQ data were log_2-transformed and missing values imputed using QRILC, and fit to a linear model with a condition–PMI interaction to identify proteins with a consistent late-*vs*.-early change in protein abundance in the dry–hypoxic condition. Proteins significantly differentially abundant at late PMI relative to early PMI (n = 245) were z-score normalised, and partitioned into two groups by k-means clustering. Within each protein cluster, hierarchical clustering using Manhattan distance with complete linkage was applied to better resolve differential abundance trends. Columns represent all samples (n = 72), split by the four experimental conditions and clustered using Manhattan distance with complete linkage.

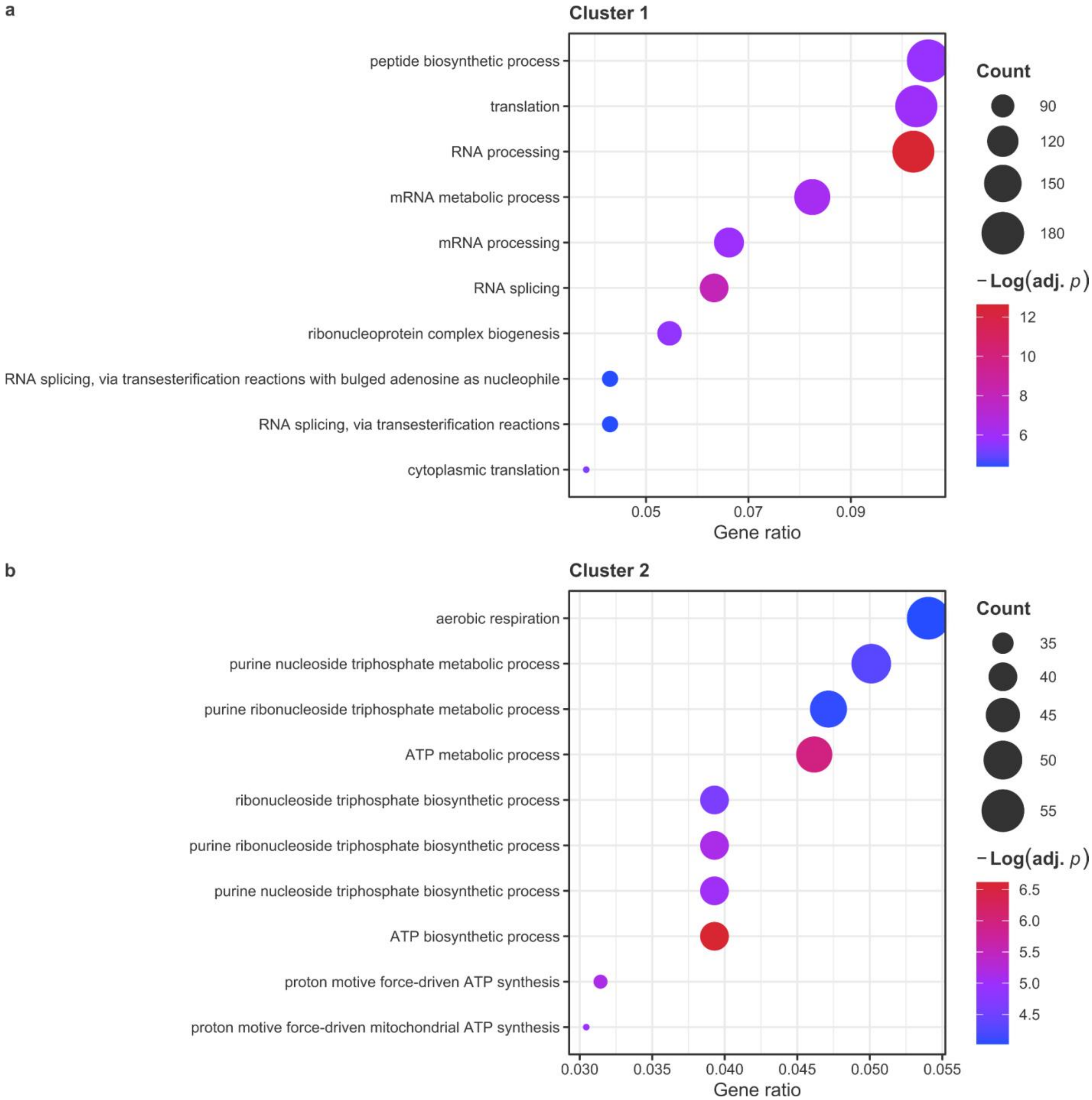

Fig S9. Biological process enrichment of proteins at early and late PMI | Enrichment analysis (GO Biological Process) of significantly differentially abundant proteins clustered by *k*-means ($n = 2{,}998$). Top 10 most significant GO terms with > 3 genes per term for **(a)** cluster 1 ($n = 1{,}886$), representing proteins more abundant in the early PMI, and for **(b)** cluster 2 ($n = 1{,}112$), proteins more abundant in the late PMI. Point size represents the number of unique proteins at 1% FDR and q-value $\leq .01$ associated with a GO term, and point colour represents the $-\log_{10}$ of adjusted p-value.

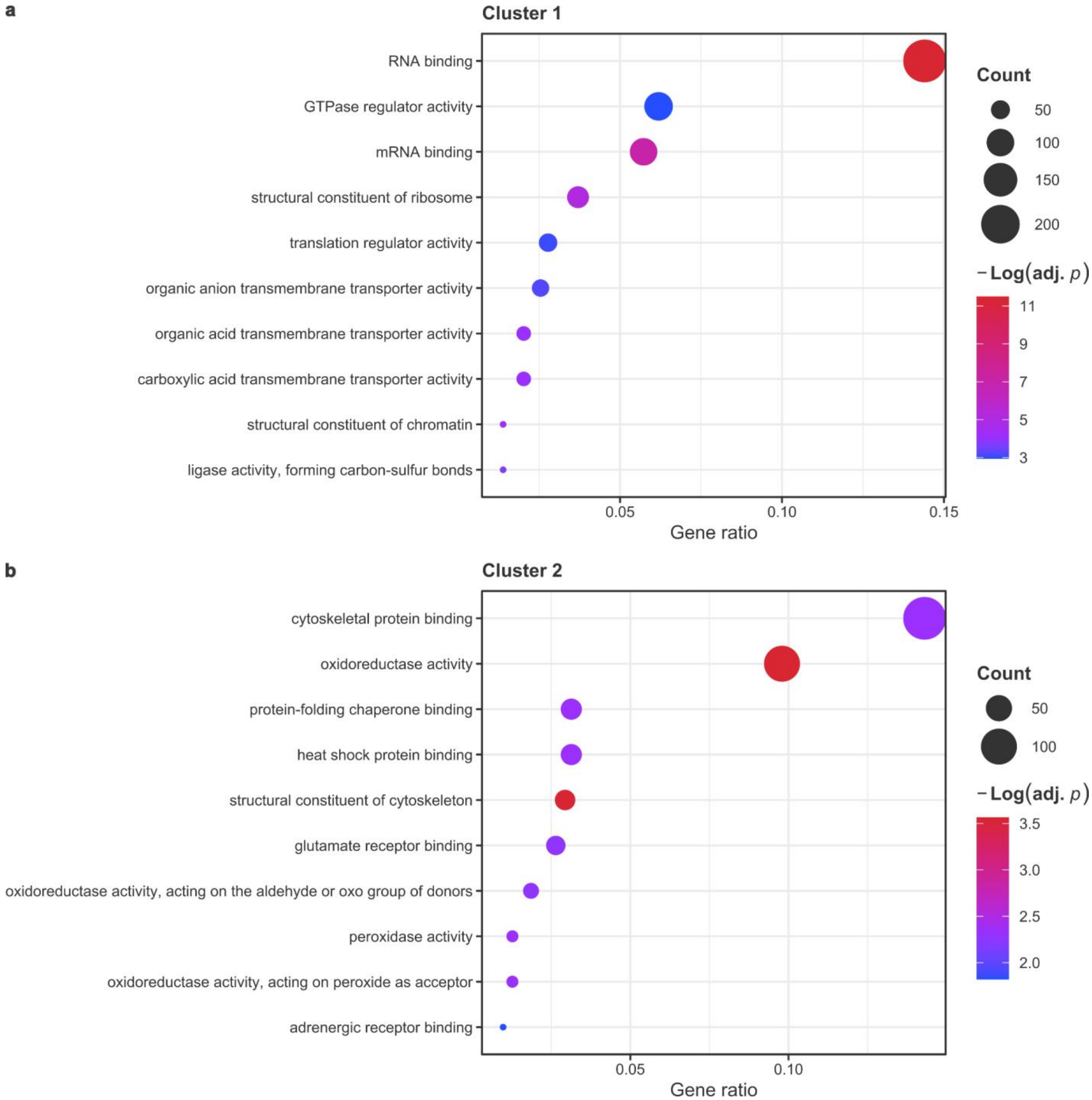

Fig S10. Molecular function enrichment of proteins at early and late PMI | Enrichment analysis (GO Molecular Function) of significantly differentially abundant proteins clustered by *k*-means ($n = 2{,}998$). Top 10 most significant GO terms with > 3 genes per term for **(a)** cluster 1 ($n = 1{,}886$), representing proteins more abundant in the early PMI, and for **(b)** cluster 2 ($n = 1{,}112$), proteins more abundant in the late PMI. Point size represents the number of unique proteins at 1% FDR and q-value ≤ .01 associated with a GO term, and point colour represents the $-\log_{10}$ of adjusted p-value.

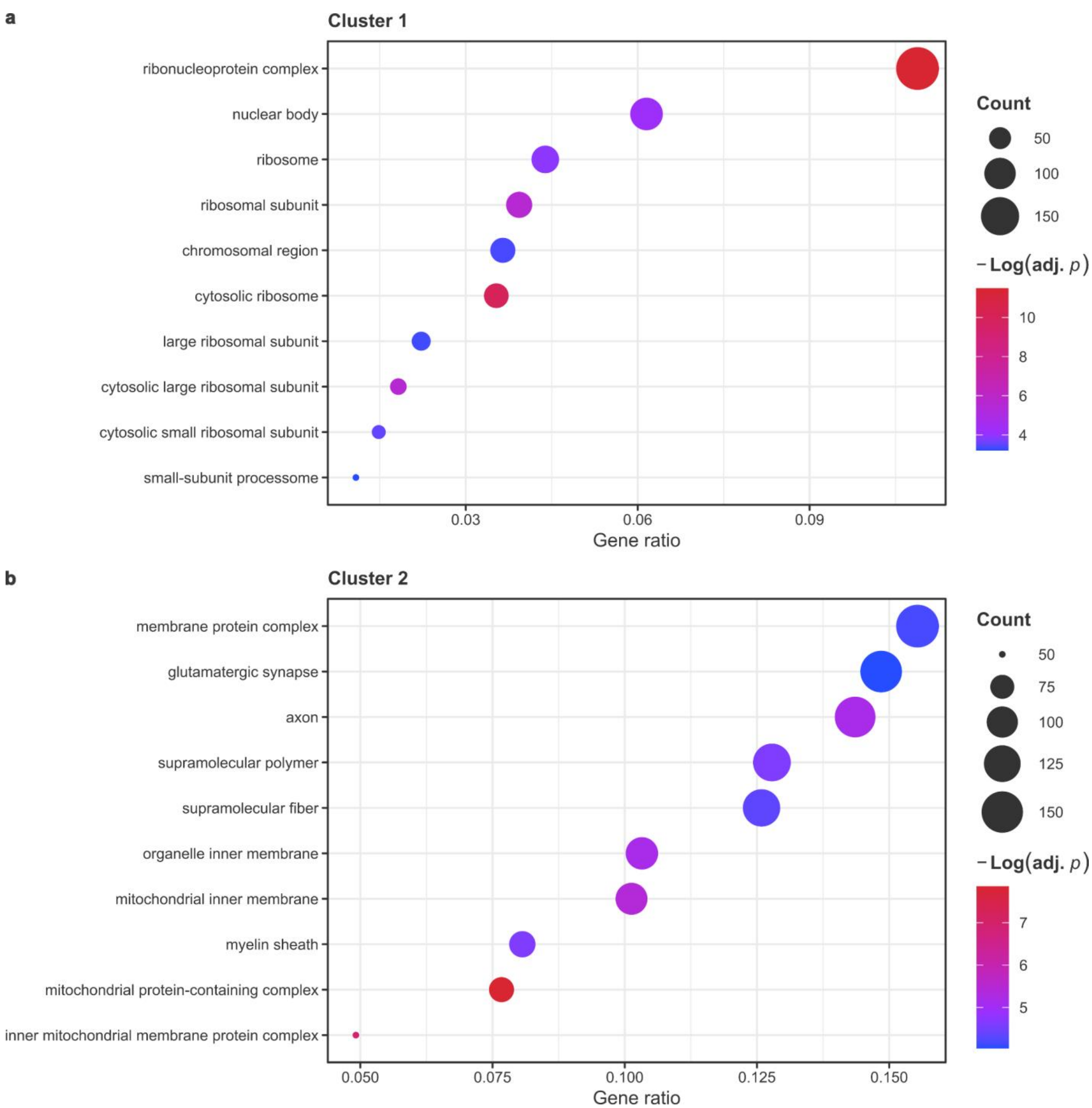

Fig S11. Cellular component enrichment of proteins at early and late PMI | Enrichment analysis (GO Cellular Component) of significantly differentially abundant proteins clustered by *k*-means ($n = 2{,}998$). Top 10 most significant GO terms with > 3 genes per term for **(a)** cluster 1 ($n = 1{,}886$), representing proteins more abundant in the early PMI, and for **(b)** cluster 2 ($n = 1{,}112$), proteins more abundant in the late PMI. Point size represents the number of unique proteins at 1% FDR and q-value ≤ .01 associated with a GO term, and point colour represents the $-\log_{10}$ of adjusted p-value.

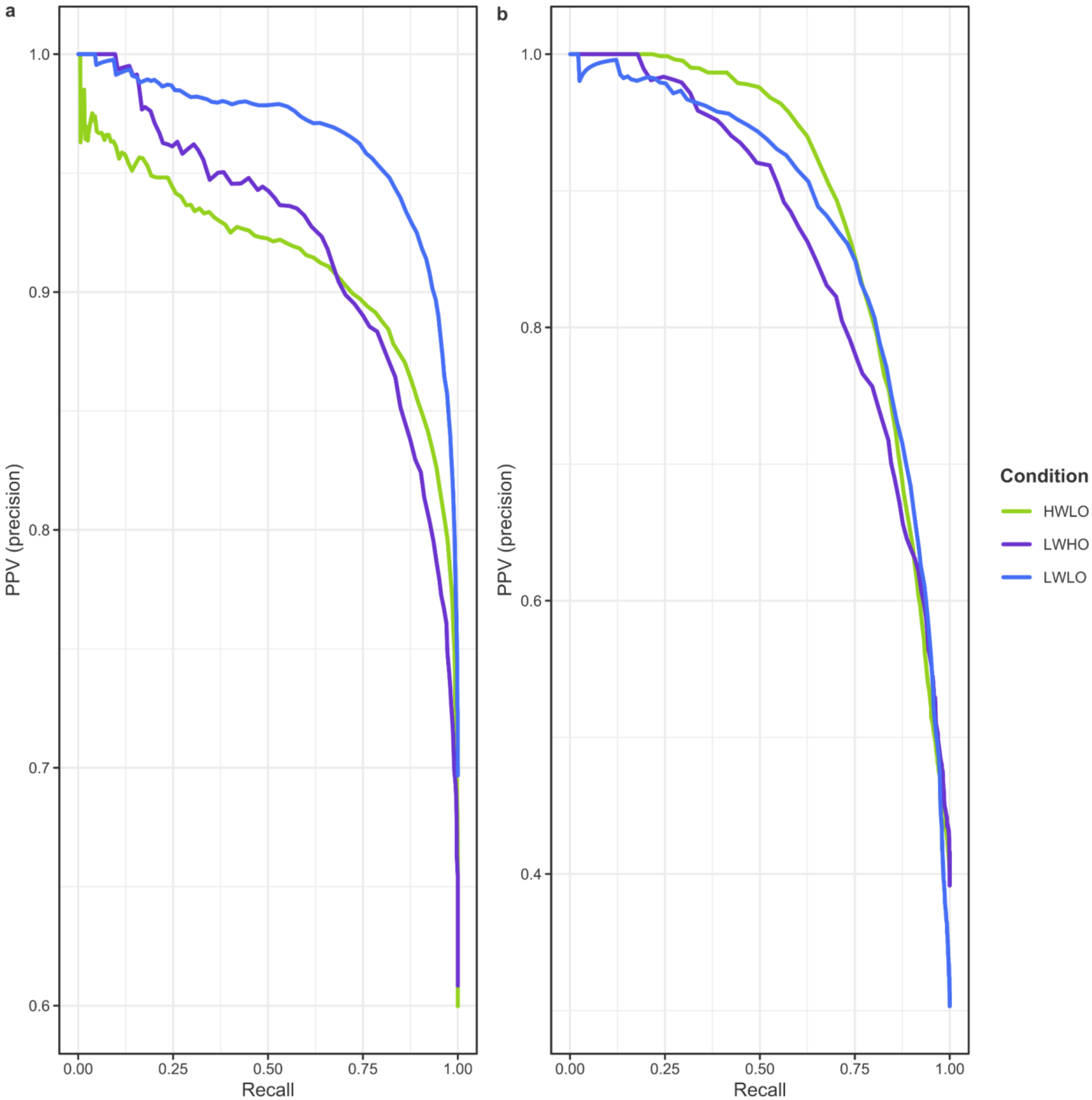

Fig S12. Precision–recall for early- and late-peaking peptide classifiers across conditions | Performance when predicting **(a)** early-peaking peptides, and **(b)** late-peaking peptides. Lines show the convex hull ('Pareto frontier') of positive predictive value (PPV; precision) *vs*. recall for peptide classification in each condition. No peptides detected in the wet–oxic condition met quality criteria, and were excluded from analysis.

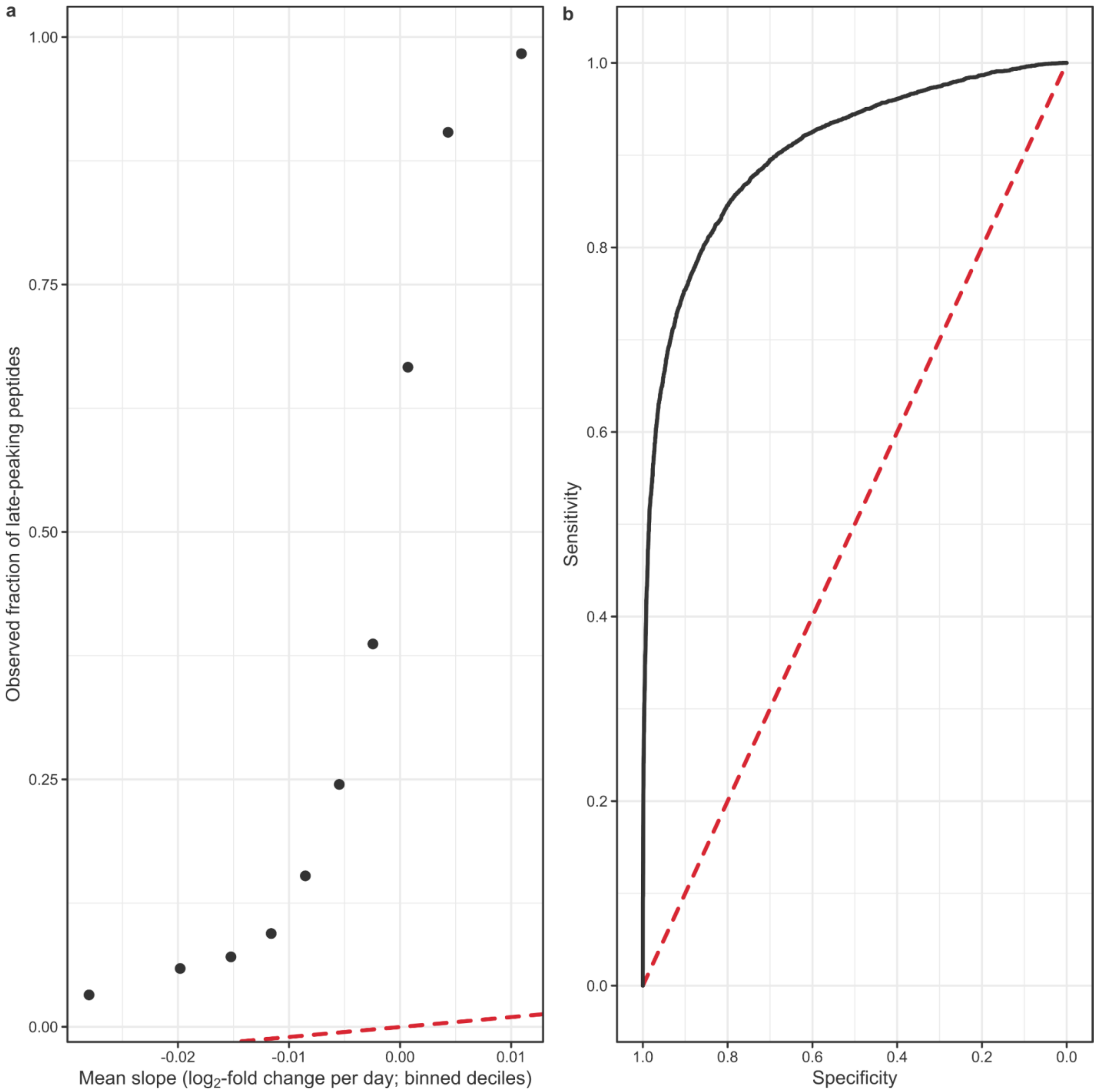

Fig S13. Calibration and discrimination of the peptide-level recalcitrance classifier | (a) Calibration plot showing the observed fraction of late-peaking peptides *vs.* the mean predicted $\log_2$-fold change slope per day, binned into deciles. Points indicate the empirical late-peaking peptide rate in each bin; the red dashed line marks the ideal (no-bias) relationship. The upward trend demonstrates that peptides with higher predicted stability slopes are increasingly likely to exhibit late-peaking behaviour. **(b)** Receiver operating characteristic (ROC) curve for distinguishing early- *vs.* late-peaking peptides. The solid black line is the classifier performance across all thresholds, and the red dashed diagonal represents random chance. The pronounced convexity of the curve indicates the strong discriminative ability of the model.

Table S4. Over-representation test results for PANTHER protein class corresponding to recalcitrant peptides (n = 2,647) identified in wet–hypoxic conditions | Only statistically significant results (i.e., Bonferroni-corrected for $p < .05$) for classified proteins are shown. Subclasses (*italics*) are indented below parent classes to illustrate hierarchical relationships. Cells containing p-values are coloured based on the following significance codes: | < .0001 | ≤ .001 | ≤ .01 | ≤ .05 | non-significant |

**Protein class**	**No. referenced**	**No. queried**	**No. expected**	**Fold enrichment**	***p*-Value**
Metabolite interconversion enzyme	737	127	53.58	2.37	1.83×10^{-21}
↳ *Oxidoreductase*	228	54	16.58	3.26	1.63×10^{-13}
↳ *Peroxidase*	9	5	0.65	7.64	3.18×10^{-2}

Protease	178	32	12.94	2.47	1.76×10^{-4}
↳ *Metalloprotease*	44	16	3.20	5.00	4.56×10^{-6}
Calcium-binding protein	43	21	3.13	6.72	2.78×10^{-11}
↳ *Calmodulin-related*	29	10	2.11	4.74	3.41×10^{-3}
Lyase	54	15	3.93	3.82	6.47×10^{-4}
Dehydrogenase	82	22	5.96	3.69	6.97×10^{-6}
Translational protein	184	2	13.38	0.15	3.49×10^{-2}
RNA metabolism protein	242	1	17.59	0.06	5.23×10^{-5}

Table S5. Over-representation test results for PANTHER protein class corresponding to recalcitrant peptides (n = 908) identified in dry–oxic conditions | Only statistically significant results (i.e., Bonferroni-corrected for $p < .05$) for classified proteins are shown. Subclasses (*italics*) are indented below parent classes to illustrate hierarchical relationships. Cells containing *p*-values are coloured based on the following significance codes: | < .0001 | ≤ .001 | ≤ .01 | ≤ .05 | non-significant |

Protein class	No. referenced	No. queried	No. expected	Fold enrichment	*p*-Value
Protease	178	39	13.53	2.88	1.09×10^{-7}
Metabolite interconversion enzyme	737	94	56.04	1.68	1.72×10^{-5}
↳ *Oxidoreductase*	228	34	17.34	1.96	1.67×10^{-2}

Table S6. Over-representation test results for PANTHER protein class corresponding to recalcitrant peptides (n = 1,977) identified in dry–hypoxic conditions | Only statistically significant results (i.e., Bonferroni-corrected for $p < .05$) for classified proteins are shown. Subclasses (*italics*) are indented below parent classes to illustrate hierarchical relationships. Cells containing *p*-values are coloured based on the following significance codes: | < .0001 | ≤ .001 | ≤ .01 | ≤ .05 | non-significant |

Protein class	No. referenced	No. queried	No. expected	Fold enrichment	*p*-Value
Metabolite interconversion enzyme	737	176	73.08	2.41	3.70×10^{-32}
↳ *Oxidoreductase*	228	77	22.61	3.41	5.58×10^{-22}
↳ *Peroxidase*	9	7	0.89	7.84	4.45×10^{-4}
Protease	178	54	17.65	3.06	1.29×10^{-12}
↳ *Metalloprotease*	44	20	4.36	4.58	1.74×10^{-7}
Dehydrogenase	82	33	8.13	4.06	4.00×10^{-11}
Lyase	54	21	5.35	3.92	2.08×10^{-6}
Calcium-binding protein	43	16	4.26	3.75	2.35×10^{-4}
Protein-binding activity modulator	292	12	28.95	0.41	4.08×10^{-2}
RNA metabolism protein	242	1	24.00	0.04	5.14×10^{-8}

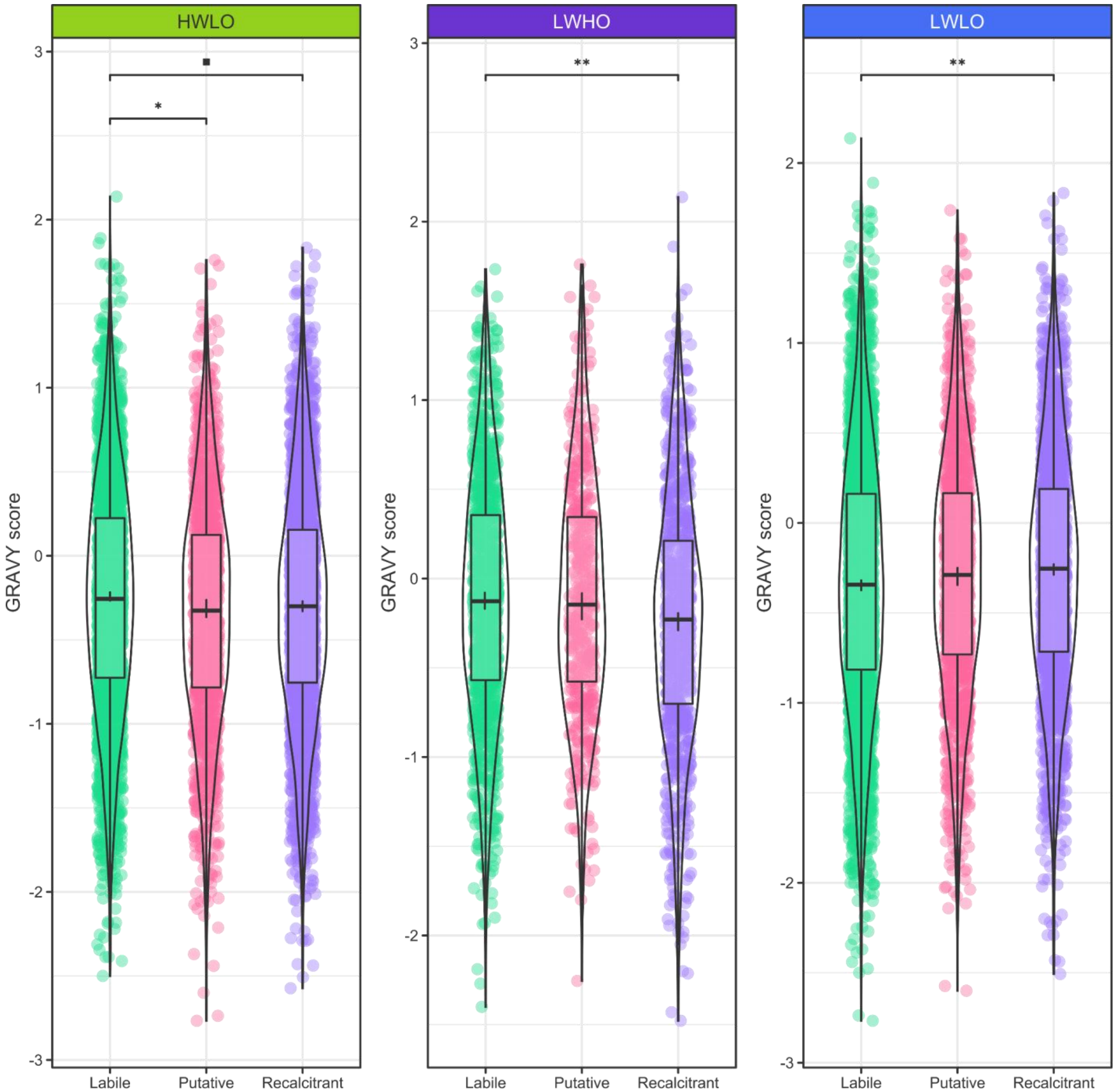

Fig S14. Hydropathy by burial environment | Results of Kruskal–Wallis tests (n_{obs} = 16,128) with eta-squared effect sizes (η^2_H) and *post-hoc* Dunn pairwise comparisons (Holm-adjusted), for peptide preservation with respect to GRAVY score. Higher GRAVY scores are more hydrophobic, and lower scores more hydrophilic. Vertical black lines intersecting the median indicate bootstrapped 95% CIs. Only statistically significant comparisons are shown (for all other comparisons, see Table S7), and adjusted significance indicated by the following codes: ∗∗∗ = *p*-value < .0001; ∗∗ = *p*-value ≤ .001; ∗ = *p*-value ≤ .01; ■ = *p*-value ≤ .05. No peptides detected in the wet–oxic condition met quality criteria, and were excluded from analysis.

Table S7. Hydropathy by preservation category | Results of Kruskal–Wallis tests with eta-squared effect sizes and *post-hoc* Dunn pairwise comparisons (Holm-adjusted), for peptide preservation with respect to GRAVY score. Cells containing *p*-values are coloured based on the following significance codes: | < .0001 | ≤ .001 | ≤ .01 | ≤ .05 | non-significant |

Condition	Comparison	*Z*-statistic	Rank biserial correlation (*r*)	Cliff's δ (95% CI)	Adjusted *p*-value
**HWLO** n_{obs} = 7,076 χ^2[2] = 12.33, *p* = 2.10 ×	Labile – Putative	3.207368558	0.03812896	0.0599 (0.0235, 0.0962)	4.01×10^{-3}
	Labile – Recalcitrant	2.548472752	0.03029606	0.0391 (0.0091, 0.0692)	2.16×10^{-2}

10^{-3}, $\eta^2_H = 0.001$	Putative – Recalcitrant	–1.087808288	0.01293178	–0.0208 (–0.0582, 0.0166)	2.77×10^{-1}
**LWHO** $n_{obs} = 2{,}467$ $\chi^2\,[2] = 15.63$, $p = 4.03 \times 10^{-4}$, $\eta^2_H = 0.006$	Labile – Putative	0.09508860388	0.001914449	0.0032 (–0.0586, 0.0649)	9.24×10^{-1}
	Labile – Recalcitrant	3.70258283307	0.074545290	0.0961 (0.0454, 0.1464)	6.40×10^{-4}
	Putative – Recalcitrant	2.85175285652	0.057415257	0.0934 (0.0298, 0.1562)	5.74×10^{-2}
**LWLO** $n_{obs} = 6{,}585$ $\chi^2\,[2] = 14.98$, $p = 5.58 \times 10^{-4}$, $\eta^2_H = 0.002$	Labile – Putative	–1.958948405	0.02414044	–0.0368 (–0.0728, –0.0007)	1.00×10^{-1}
	Labile – Recalcitrant	–3.798806373	0.04681330	–0.0624 (–0.0942, –0.0305)	4.36×10^{-4}
	Putative – Recalcitrant	–1.263007731	0.01556425	–0.0259 (–0.0659, 0.0141)	2.07×10^{-1}

Table S8. Net charge by preservation category | Results of Kruskal–Wallis tests with eta-squared effect sizes and *post-hoc* Dunn pairwise comparisons (Holm-adjusted), for peptide preservation with respect to weighted mean net charge. Cells containing *p*-values are coloured based on the following significance codes: | < .0001 | ≤ .001 | ≤ .01 | ≤ .05 | non-significant |

Condition	Comparison	*Z*-statistic	Rank biserial correlation (*r*)	Cliff's δ (95% CI)	Adjusted *p*-value
**HWLO** $n_{obs} = 7{,}076$ $\chi^2\,[2] = 2.33$, $p = 3.11 \times 10^{-1}$, $\eta^2_H = 0.000$	Labile – Putative	0.7480607522	0.008892891	0.0141 (–0.0227, 0.0509)	4.54×10^{-1}
	Labile – Recalcitrant	–0.9439390356	0.011221477	–0.0145 (–0.0447, 0.0156)	6.90×10^{-1}
	Putative – Recalcitrant	–1.4899610831	0.017712547	–0.0284 (–0.0657, 0.0091)	4.09×10^{-1}
**LWHO** $n_{obs} = 2{,}467$ $\chi^2\,[2] = 5.46$, $p = 6.52 \times 10^{-2}$, $\eta^2_H = 0.001$	Labile – Putative	–0.594087599	0.01196096	–0.0192 (–0.0809, 0.0427)	5.52×10^{-1}
	Labile – Recalcitrant	1.875285318	0.03775572	0.0489 (–0.0021, 0.0997)	1.22×10^{-1}
	Putative – Recalcitrant	2.068503184	0.04164584	0.0672 (0.0034, 0.1305)	1.16×10^{-1}
**LWLO** $n_{obs} = 6{,}585$ $\chi^2\,[2] = 9.37$, $p = 9.22 \times 10^{-3}$, $\eta^2_H = 0.001$	Labile – Putative	–1.9329071807	0.02381953	–0.0361 (–0.0726, 0.0004)	1.06×10^{-1}
	Labile – Recalcitrant	–2.8802900065	0.03549428	–0.0474 (–0.0795, –0.0153)	1.19×10^{-2}
	Putative – Recalcitrant	–0.5472783887	0.00674420	–0.0111 (–0.0511, 0.0289)	5.84×10^{-1}

Table S9. Isoelectric point by preservation category | Results of Kruskal–Wallis tests with eta-squared effect sizes and *post-hoc* Dunn pairwise comparisons (Holm-adjusted), for peptide preservation with respect to predicted isoelectric point (pI). Cells containing *p*-values are coloured based on the following significance codes: | < .0001 | ≤ .001 | ≤ .01 | ≤ .05 | non-significant |

Condition	Comparison	*Z*-statistic	Rank biserial correlation (*r*)	Cliff's δ (95% CI)	Adjusted *p*-value
**HWLO** $n_{obs} = 7{,}076$ $\chi^2\,[2] = 3.36$, $p = 1.86 \times$	Labile – Putative	0.5709277791	0.006787147	0.0107 (–0.0260, 0.0473)	5.68×10^{-1}
	Labile – Recalcitrant	–1.3913451549	0.016540208	–0.0214 (–0.0514, 0.0087)	3.28×10^{-1}

10^{-1}, η^2_H = 0.000	Putative – Recalcitrant	–1.6763287149	0.019928071	–0.0320 (–0.0694, 0.0054)	2.81 × 10^{-1}
**LWHO**<br>n_{obs} = 2,467<br>χ^2 [2] = 4.32, p = 1.15 × 10^{-1}, η^2_H = 0.001	Labile – Putative	–1.008321662	0.02030086	–0.0322 (–0.0944, 0.0304)	3.13 × 10^{-1}
	Labile – Recalcitrant	1.311120668	0.02639721	0.0341 (–0.0168, 0.0849)	3.80 × 10^{-1}
	Putative – Recalcitrant	2.022458615	0.04071881	0.0659 (0.0013, 0.1299)	1.29 × 10^{-1}
**LWLO**<br>n_{obs} = 6,585<br>χ^2 [2] = 3.97, p = 1.37 × 10^{-1}, η^2_H = 0.000	Labile – Putative	–1.6524078210	0.020362887	–0.0309 (–0.0677, 0.0061)	2.95 × 10^{-1}
	Labile – Recalcitrant	–1.5996565929	0.019712825	–0.0264 (–0.0586, 0.0059)	2.19 × 10^{-1}
	Putative – Recalcitrant	0.2267609643	0.002794412	0.0047 (–0.0354, 0.0448)	8.21 × 10^{-1}

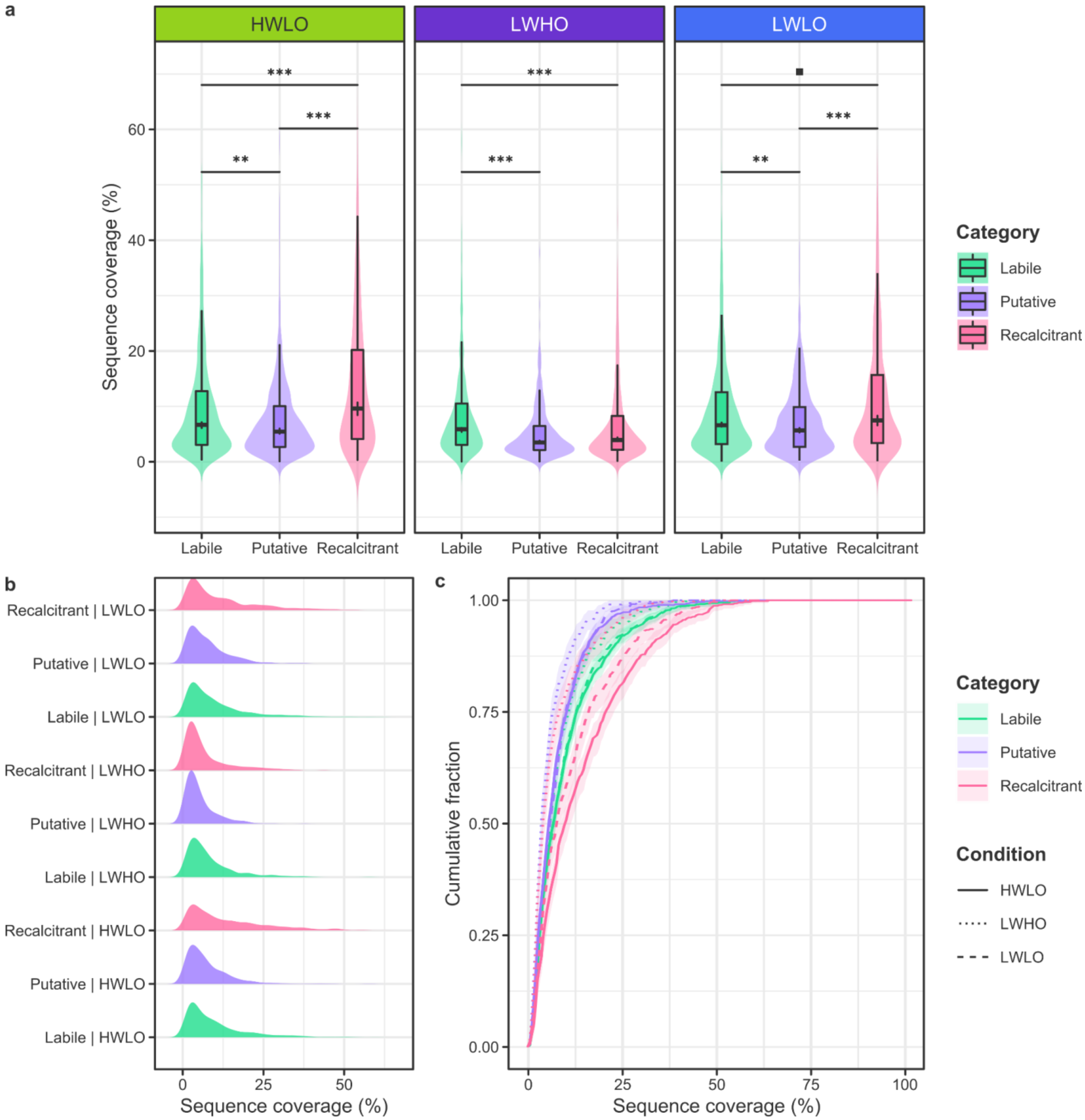

Fig S15. Sequence coverage distributions by preservation category within burial environments | (a) Violin–box plots illustrate distributions of sequence coverage (%) across proteins for labile, putative, and recalcitrant peptides in each burial condition (n = 5,445). Vertical black lines intersecting the median indicate bootstrapped 95% CIs. Only statistically significant comparisons are shown (for all other comparisons, see Table S12), and adjusted significance indicated by the following codes: ∗∗∗ = p-value < .0001; ∗∗ = p-value ≤ .001; ∗ = p-value ≤ .01; ■ = p-value ≤ .05. No peptides detected in the wet–oxic condition met quality criteria, and were excluded from analysis. **(b)** Ridgeline density plots illustrate the coverage distributions for each category–condition combination. **(c)** ECDFs depict cumulative fractions of sequence coverage with 95% CIs, coloured by preservation category and styled by condition.

Table S10. Mean peptide length and sequence coverage by preservation category | Results of Kruskal–Wallis tests with eta-squared effect sizes and *post-hoc* Dunn pairwise comparisons (Holm-adjusted), for peptide preservation with respect to mean peptide length and sequence coverage (%). Cells containing p-values are coloured based on the following significance codes: | < .0001 | ≤ .001 | ≤ .01 | | ≤ .05 | non-significant |

Metric	Comparison	*Z*-statistic	Rank biserial correlation (*r*)	Cliff's δ (95% CI)	Adjusted *p*-value
**Mean peptide**	Labile – Putative	2.73647169	0.05034840	0.0704 (0.0208, 0.1197)	1.86×10^{-2}

**length**<br>n_{obs} = 2,954<br>χ^2 [2] = 7.52, p = 2.33 × 10^{-2}, η^2_H = 0.002	Labile – Recalcitrant	1.25897150	0.02316385	0.0314 (–0.0211, 0.0836)	2.08 × 10^{-1}
	Putative – Recalcitrant	–1.29000399	0.02373481	–0.0341 (–0.0893, 0.0213)	3.94 × 10^{-1}
**Sequence coverage**<br>n_{obs} = 2,954<br>χ^2 [2] = 19.15, p = 6.95 × 10^{-5}, η^2_H = 0.006	Labile – Putative	3.94602015	0.07260291	0.1010 (0.0518, 0.1496)	2.38 × 10^{-4}
	Labile – Recalcitrant	–0.14532188	0.00267378	–0.0052 (–0.0573, 0.0469)	8.84 × 10^{-1}
	Putative – Recalcitrant	–3.68236764	0.06775197	–0.1015 (–0.1562, –0.4612)	4.62 × 10^{-4}

Table S11. Mean peptide length by preservation category | Results of Kruskal–Wallis tests with eta-squared effect sizes and *post-hoc* Dunn pairwise comparisons (Holm-adjusted), for peptide preservation with respect to mean peptide length. Cells containing *p*-values are coloured based on the following significance codes: | < .0001 | ≤ .001 | ≤ .01 | ≤ .05 | non-significant |

Condition	Comparison	*Z*-statistic	Rank biserial correlation (*r*)	Cliff's δ (95% CI)	Adjusted *p*-value
**HWLO**<br>n_{obs} = 2,113<br>χ^2 [2] = 17.25, p = 1.80 × 10^{-5}, η^2_H = 0.007	Labile – Putative	–0.59146112	0.01286089	–0.0189 (–0.0783, 0.0406)	5.54 × 10^{-1}
	Labile – Recalcitrant	–4.04100786	0.087786876	–0.1220 (–0.1812, –0.0620)	1.60 × 10^{-4}
	Putative – Recalcitrant	–3.06060522	0.06655062	–0.1064 (–0.1725, –0.0393)	4.42 × 10^{-3}
**LWHO**<br>n_{obs} = 1,186<br>χ^2 [2] = 15.22, p = 4.95 × 10^{-4}, η^2_H = 0.011	Labile – Putative	3.68976836	0.10700601	0.1566 (0.0739, 0.2372)	6.73 × 10^{-4}
	Labile – Recalcitrant	2.73263102	0.07924832	0.1055 (0.0275, 0.1821)	1.26 × 10^{-2}
	Putative – Recalcitrant	–1.08250440	0.03139343	–0.0441 (–0.1283, 0.0407)	2.79 × 10^{-1}
**LWLO**<br>n_{obs} = 2,139<br>χ^2 [2] = 3.97, p = 1.38 × 10^{-1}, η^2_H = 0.001	Labile – Putative	1.85763162	0.04014683	0.0565 (–0.0027, 0.1153)	1.90 × 10^{-1}
	Labile – Recalcitrant	–0.02495404	0.00053930	–0.0017 (–0.065, 0.0614)	9.80 × 10^{-1}
	Putative – Recalcitrant	–1.62510418	0.03512149	–0.0547 (–0.1227, 0.0137)	2.08 × 10^{-1}

Table S12. Sequence coverage by preservation category | Results of Kruskal–Wallis tests with eta-squared effect sizes and *post-hoc* Dunn pairwise comparisons (Holm-adjusted), for peptide preservation with respect to sequence coverage (%). Cells containing *p*-values are coloured based on the following significance codes: | < .0001 | ≤ .001 | ≤ .01 | ≤ .05 | non-significant |

Condition	Comparison	*Z*-statistic	Rank biserial correlation (*r*)	Cliff's δ (95% CI)	Adjusted *p*-value
**HWLO**<br>n_{obs} = 2,113<br>χ^2 [2] = 83.35, p = 7.95 × 10^{-19}, η^2_H = 0.039	Labile – Putative	3.84656026	0.08364064	0.1179 (0.0594, 0.1756)	1.20 × 10^{-4}
	Labile – Recalcitrant	–6.29214607	0.13681812	–0.1921 (–0.2508, –0.1320)	6.26 × 10^{-10}
	Putative – Recalcitrant	–9.00663721	0.19584274	–0.3080 (–0.3700, –0.2433)	6.37 × 10^{-19}

**LWHO**<br>$n_{obs} = 1{,}186$<br>$\chi^2[2] = 47.60$, $p = 4.61 \times 10^{-11}$, $\eta^2_H = 0.038$	Labile – Putative	6.51395470	0.18890950	0.2781 (0.1985, 0.3542)	2.20×10^{-10}
	Labile – Recalcitrant	4.85496954	0.14079771	0.1870 (0.1096, 0.2621)	2.41×10^{-6}
	Putative – Recalcitrant	–1.88318300	0.05461370	0.0756 (–0.0090, –0.1592)	5.97×10^{-2}
**LWLO**<br>$n_{obs} = 2{,}139$<br>$\chi^2[2] = 30.92$, $p = 1.93 \times 10^{-7}$, $\eta^2_H = 0.014$	Labile – Putative	3.93215233	0.08498103	0.1191 (0.0613, 0.1760)	1.68×10^{-4}
	Labile – Recalcitrant	–2.31336913	0.04999615	–0.0728 (–0.1344, –0.0106)	2.07×10^{-2}
	Putative – Recalcitrant	–5.44997404	0.11778395	–0.1871 (–0.2522, –0.1204)	1.51×10^{-7}

Table S13. Secondary structure by preservation category | Results of linear regression for secondary-structure composition *vs.* preservation category, with β-sheet as the reference term and sensitivity analyses across mean pLDDT confidence thresholds. Cells containing *p*-values are coloured based on the following significance codes: | $< .0001$ | $\leq .001$ | $\leq .01$ | $\leq .05$ | non-significant |

Sensitivity	Term	Coefficient (β)	SE	Z-statistic	Adjusted *p*-value
No filtering on mean pLDDT<br>$n_{obs} = 96{,}768$	β-Sheet fraction	–2.58228674	0.0934783560	–27.624435	5.66×10^{-168}
	α-Helix fraction	–0.53888843	0.0245911712	–21.913899	1.91×10^{-106}
	Coil fraction	0.18639132	0.0701186006	2.658229	7.86×10^{-3}
	Mean pLDDT	0.02737770	0.0009301053	29.435057	1.96×10^{-190}
	Peptide length	–0.02711139	0.0020836560	–13.011450	1.05×10^{-38}
Peptides with mean pLDDT ≥ 50<br>$n_{obs} = 89{,}976$	β-Sheet fraction	–2.76951304	0.102112373	–27.122208	5.39×10^{-162}
	α-Helix fraction	–0.53844709	0.024664474	–21.830877	1.81×10^{-105}
	Coil fraction	0.13267369	0.070980906	1.869146	6.16×10^{-2}
	Mean pLDDT	0.02936129	0.001031122	28.475083	2.38×10^{-178}
	Peptide length	–0.02650022	0.002087615	–12.694017	6.38×10^{-37}
Peptides with mean pLDDT ≥ 70<br>$n_{obs} = 87{,}588$	β-Sheet fraction	–3.47224920	0.127291468	–27.277941	7.75×10^{-164}
	α-Helix fraction	–0.5662120	0.025001476	–22.663510	1.03×10^{-113}
	Coil fraction	0.06676381	0.072674818	0.918665	3.58×10^{-1}
	Mean pLDDT	0.03706986	0.001321425	28.052931	3.68×10^{-173}

	Peptide length	–0.02696231	0.002109278	–12.782718	2.05×10^{-37}

Table S14. Weighted α-helix fraction by preservation category and burial environment | Results of Kruskal–Wallis tests with eta-squared effect sizes and *post-hoc* Dunn pairwise comparisons (Holm-adjusted), for peptide preservation with respect to weighted α-helix fraction. Cells containing *p*-values are coloured based on the following significance codes: | < .0001 | ≤ .001 | ≤ .01 | ≤ .05 | non-significant |

Condition	Comparison	*Z*-statistic	Rank biserial correlation (*r*)	Cliff's δ (95% CI)	Adjusted *p*-value
**HWLO** n_{obs} = 39,594 χ^2 [2] = 301.16, p = 4.01 × 10^{-66}, η^2_H = 0.007	Labile – Putative	11.168387	0.05420270	0.0881 (0.0724, 0.10380	1.16×10^{-28}
	Labile – Recalcitrant	16.550766	0.08032460	0.1075 (0.0948, 0.1200)	4.74×10^{-61}
	Putative – Recalcitrant	2.253625	0.0193735	0.0175 (0.0015, 0.0334)	2.42×10^{-2}
**LWHO** n_{obs} = 14,178 χ^2 [2] = 72.32, p = 1.98 × 10^{-16}, η^2_H = 0.005	Labile – Putative	2.483007	0.02040882	0.0311 (0.0046, 0.0576)	1.30×10^{-2}
	Labile – Recalcitrant	8.473433	0.06964648	0.0929 (0.0714, 0.1143)	7.25×10^{-17}
	Putative – Recalcitrant	4.350706	0.03576017	0.0573 (0.0309, 0.0835)	2.71×10^{-5}
**LWLO** n_{obs} = 36,882 χ^2 [2] = 313.32, p = 9.18 × 10^{-69}, η^2_H = 0.008	Labile – Putative	13.403065045	0.0674296042	0.1064 (0.0907, 0.1219)	1.16×10^{-40}
	Labile – Recalcitrant	15.389466905	0.0774230117	0.1065 (0.0930, 0.1199)	5.78×10^{-53}
	Putative – Recalcitrant	–0.009009425	0.0000453256	–0.0003 (–0.0172, 0.0166)	9.93×10^{-1}

Table S15. Weighted β-sheet fraction by preservation category and burial environment | Results of Kruskal–Wallis tests with eta-squared effect sizes and *post-hoc* Dunn pairwise comparisons (Holm-adjusted), for peptide preservation with respect to weighted β-sheet fraction. Cells containing *p*-values are coloured based on the following significance codes: | < .0001 | ≤ .001 | ≤ .01 | ≤ .05 | non-significant |

Condition	Comparison	*Z*-statistic	Rank biserial correlation (*r*)	Cliff's δ (95% CI)	Adjusted *p*-value
**HWLO** n_{obs} = 39,594 χ^2 [2] = 428.29, p = 9.96 × 10^{-94}, η^2_H = 0.010	Labile – Putative	–9.617936	0.04667801	–0.0754 (–0.0910, –0.0598)	1.34×10^{-21}
	Labile – Recalcitrant	–20.612019	0.10003477	–0.1334 (–0.1459, –0.1209)	6.42×10^{-94}
	Putative – Recalcitrant	–7.018982	0.03406470	–0.0557 (–0.0716, –0.0399)	2.23×10^{-12}
**LWHO** n_{obs} = 14,178 χ^2 [2] = 26.09, p = 2.16 × 10^{-6}, η^2_H = 0.002	Labile – Putative	–0.9481579	0.007793283	–0.0107 (–0.0368, 0.0154)	3.43×10^{-1}
	Labile – Recalcitrant	–5.0116519	0.041192741	–0.0552 (–0.0764, –0.0339)	1.62×10^{-6}
	Putative – Recalcitrant	–3.0775126	0.025295289	–0.0396 (–0.0660, –0.0132)	4.17×10^{-3}
**LWLO**	Labile – Putative	–15.772470	0.07934987	–0.1237 (–0.1391, –0.1083)	9.63×10^{-56}

n_{obs} = 36,882<br>χ^2 [2] = 558.49, p = 5.31 × 10^{-122}, η^2_H = 0.014	Labile – Recalcitrant	–21.871378	0.11003292	–0.1516 (–0.1645, –0.1383)	1.46×10^{-105}
	Putative – Recalcitrant	–3.011473	0.01515045	–0.0239 (–0.0408, –0.0070)	2.60×10^{-3}

Table S16. Predicted secondary structure model confidence by preservation category | Results of Kruskal–Wallis tests with eta-squared effect sizes and *post-hoc* Dunn pairwise comparisons (Holm-adjusted), for peptide preservation with respect to mean pLDDT. Cells containing *p*-values are coloured based on the following significance codes: | < .0001 | ≤ .001 | ≤ .01 | ≤ .05 | non-significant |

Condition	Comparison	*Z*-statistic	Rank biserial correlation (*r*)	Cliff's δ (95% CI)	Adjusted *p*-value
**HWLO**<br>n_{obs} = 39,594<br>χ^2 [2] = 732.48, p = 8.78 × 10^{-160}, η^2_H = 0.019	Labile – Putative	–2.991183	0.01503240	–0.0219 (–0.0377, –0.0062)	2.78×10^{-3}
	Labile – Recalcitrant	–25.988297	0.13060600	–0.1691 (–0.1816, –0.1567)	2.01×10^{-148}
	Putative – Recalcitrant	–17.817824	0.08954472	–0.1423 (–0.1580, –0.1265)	1.03×10^{-70}
**LWHO**<br>n_{obs} = 14,178<br>χ^2 [2] = 65.71, p = 5.39 × 10^{-15}, η^2_H = 0.005	Labile – Putative	4.001498	0.03360587	0.0528 (0.0270, 0.0786)	1.26×10^{-4}
	Labile – Recalcitrant	8.032175	0.06745679	0.0872 (0.0660, 0.1083)	2.87×10^{-15}
	Putative – Recalcitrant	2.527517	0.021226990	0.0344 (0.0079, 0.0609)	1.15×10^{-2}
**LWLO**<br>n_{obs} = 36,882<br>χ^2 [2] = 1237.90, p = 1.54 × 10^{-269}, η^2_H = 0.034	Labile – Putative	–18.71997	0.09747603	–0.1482 (–0.1635, –0.1328)	6.81×10^{-78}
	Labile – Recalcitrant	–34.34527	0.17883797	–0.2378 (–0.2508, –0.2249)	4.97×10^{-258}
	Putative – Recalcitrant	–10.31255	0.05369809	–0.0879 (–0.1047, –0.0710)	6.18×10^{-25}

Table S17. Intrinsic disorder by preservation category | Results of Kruskal–Wallis tests with eta-squared effect sizes and *post-hoc* Dunn pairwise comparisons (Holm-adjusted), for peptide preservation with respect to mean IUPred score and fraction disordered. Cells containing *p*-values are coloured based on the following significance codes: | < .0001 | ≤ .001 | ≤ .01 | ≤ .05 | non-significant |

Metric	Comparison	*Z*-statistic	Rank biserial correlation (*r*)	Cliff's δ (95% CI)	Adjusted *p*-value
**Mean IUPred score**<br>n_{obs} = 96,708<br>χ^2 [2] = 74.83, p = 5.63 × 10^{-17}, η^2_H = 0.003	Labile – Putative	6.230833	0.02003618	0.0306 (0.0207, 0.0404)	4.64×10^{-10}
	Labile – Recalcitrant	17.916210	0.05761226	0.0754 (0.0672, 0.0836)	2.64×10^{-71}
	Putative – Recalcitrant	8.424120	0.02708902	0.0434 (0.0331, 0.0537)	7.27×10^{-17}
**Fraction disordered**<br>n_{obs} = 96,708<br>χ^2 [2] = 416.09, p = 4.45 × 10^{-91}, η^2_H = 0.004	Labile – Putative	3.705416	0.01191532	0.0120 (0.0054, 0.0186)	2.11×10^{-4}
	Labile – Recalcitrant	19.246871	0.06189120	0.0529 (0.0477, 0.0582)	4.50×10^{-82}
	Putative – Recalcitrant	11.897370	0.03825778	0.0406 (0.0340, 0.0472)	2.44×10^{-32}

Table S18. Mean IUPred score by preservation category and burial environment | Results of Kruskal–Wallis tests with eta-squared effect sizes and *post-hoc* Dunn pairwise comparisons (Holm-adjusted), for peptide preservation with respect to mean IUPred score. Cells containing *p*-values are coloured based on the following significance codes: | < .0001 | ≤ .001 | ≤ .01 | ≤ .05 | non-significant |

Condition	Comparison	*Z*-statistic	Rank biserial correlation (*r*)	Cliff's δ (95% CI)	Adjusted *p*-value
**HWLO** n_{obs} = 42,438 χ^2[2] = 74.83, p = 5.63 × 10^{-17}, η^2_H = 0.002	Labile – Putative	–1.5053995	0.007307592	–0.0121 (–0.0272, 0.0030)	1.32×10^{-1}
	Labile – Recalcitrant	7.2916237	0.035395397	0.0460 (0.0338, 0.0583)	6.12×10^{-13}
	Putative – Recalcitrant	7.3332058	0.035597247	0.0565 (0.0410, 0.0719)	6.74×10^{-13}
**LWHO** n_{obs} = 14,784 χ^2[2] = 66.92, p = 2.94 × 10^{-15}, η^2_H = 0.004	Labile – Putative	7.7053877	0.063372161	0.1012 (0.0759, 0.1263)	3.91×10^{-14}
	Labile – Recalcitrant	0.3292826	0.002708151	0.0028 (–0.0180, 0.0237)	7.42×10^{-1}
	Putative – Recalcitrant	–7.2269352	0.059437179	–0.0949, –0.1204, –0.0693)	9.88×10^{-13}
**LWLO** n_{obs} = 39,486 χ^2[2] = 416.09, p = 4.45 × 10^{-91}, η^2_H = 0.011	Labile – Putative	6.6022014	0.03322517	0.0501 (0.0352, 0.0650)	4.05×10^{-1}
	Labile – Recalcitrant	20.3975198	0.10264925	0.1373 (0.1244, 0.1501)	5.28×10^{-92}
	Putative – Recalcitrant	10.3745887	0.05220947	0.0860 (0.0700, 0.1023)	6.47×10^{-25}

Table S19. Fraction disordered by preservation category and burial environment | Results of Kruskal–Wallis tests with eta-squared effect sizes and *post-hoc* Dunn pairwise comparisons (Holm-adjusted), for peptide preservation with respect to fraction disordered. Cells containing *p*-values are coloured based on the following significance codes: | < .0001 | ≤ .001 | ≤ .01 | ≤ .05 | non-significant |

Condition	Comparison	*Z*-statistic	Rank biserial correlation (*r*)	Cliff's δ (95% CI)	Adjusted *p*-value
**HWLO** n_{obs} = 42,438 χ^2[2] = 94.19, p = 3.52 × 10^{-21}, η^2_H = 0.002	Labile – Putative	–2.921652	0.01418244	–0.0155 (–0.0261, –0.0049)	3.48×10^{-3}
	Labile – Recalcitrant	7.429372	0.03606406	0.0318 (0.0236, 0.0400)	2.18×10^{-13}
	Putative – Recalcitrant	8.828818	0.04285733	0.0464 (0.0359, 0.0569)	3.17×10^{-18}
**LWHO** n_{obs} = 14,784 χ^2[2] = 42.77, p = 5.12 × 10^{-10}, η^2_H = 0.003	Labile – Putative	5.473608	0.04501712	0.0383 (0.0260, 0.0506)	8.82×10^{-8}
	Labile – Recalcitrant	–1.275147	0.01048731	–0.0074 (–0.0189, 0.0041)	2.02×10^{-1}
	Putative – Recalcitrant	–6.334320	0.05209595	–0.0448 (–0.0576, –0.0320)	7.15×10^{-10}
**LWLO** n_{obs} = 39,486 χ^2[2] = 509.05, p = 4.45 × 10^{-91}, η^2_H = 0.011	Labile – Putative	5.916831	0.02977609	0.0301 (0.0198, 0.0404)	3.28×10^{-9}
	Labile – Recalcitrant	22.503121	0.11324556	0.1007 (0.0925, 0.1088)	1.16×10^{-111}
	Putative – Recalcitrant	12.698857	0.06390621	0.0705 (0.0602, 0.0808)	1.20×10^{-36}

Table S20. UniProt feature section by preservation category | Chi-squared association tests were conducted on 215,088 peptide–UniProt feature observations corresponding to 94,296 unique peptides. Pairwise comparisons are omitted because section-level labels serve only as hierarchical groupings of feature types; reported statistics therefore describe global compositional bias rather than individual feature effects. Cells containing *p*-values are coloured based on the following significance codes: | < .0001 | ≤ .001 | ≤ .01 | ≤ .05 | non-significant |

Section	X^2 [2] statistic	Cramér's *V*	Adjusted *p*-value
Topology	768.61	0.09028322679	1.00×10^{-166}
Repeats	222.52	0.0485782804	1.91×10^{-48}
Domains and regions	220.92	0.04840298907	2.84×10^{-48}
PTM	208.65	0.04703917137	9.86×10^{-46}
Molecule processing	133.12	0.03757236843	1.99×10^{-29}
Variants/mutagenesis	60.94	0.0254216548	7.80×10^{-14}
Sites	13.71	0.01205916729	1.2×10^{-3}
Other	5.39	0.00755911831	6.8×10^{-2}

Table S21. UniProt feature type by preservation category | Chi-squared association tests were conducted on 215,088 peptide–UniProt feature observations corresponding to 94,296 unique peptides. Pairwise comparisons are omitted because type-level labels serve only as hierarchical groupings of feature descriptors; reported statistics therefore describe global compositional bias rather than individual feature effects. Cells containing *p*-values are coloured based on the following significance codes: | < .0001 | ≤ .001 | ≤ .01 | ≤ .05 | non-significant |

Section	Type	X^2 [2] statistic	Cramér's *V*	Adjusted *p*-value
Domains and regions	Region	797.35	0.1527306007	2.16×10^{-173}
	Domain	591.93	0.1315937602	4.37×10^{-129}
	Motif	16.03	0.02165634928	3.30×10^{-4}
Molecule processing	Transit peptide	32.29	0.01867474088	4.86×10^{-7}
	Chain	22.13	0.0154597819	2.61×10^{-5}
	Signal	22.26	0.01550450353	2.61×10^{-5}
	Propeptide	15.96	0.01313020300	3.51×10^{-4}
	Peptide	15.91	0.0131084283	3.51×10^{-4}
PTM	Disulfide bond	1,699.23	0.2723176685	$< 2.22 \times 10^{-354}$
	Modified residue	1,396.35	0.2468572707	1.53×10^{-303}
	Cross-link	135.55	0.07691361418	6.12×10^{-30}
	Lipidation	27.28	0.0345024968	1.49×10^{-6}
	Glycosylation	0.22	0.003068974986	8.98×10^{-1}

Sites	DNA binding	66.87	0.08375045275	1.51×10^{-14}
	Zinc finger	30.25	0.05632493097	6.76×10^{-7}
	Active site	16.80	0.04197409596	3.75×10^{-4}
	Binding site	12.69	0.03648514223	2.19×10^{-3}
	Site	5.09	0.02311053736	7.84×10^{-2}
Topology	Transmembrane	51.75	0.05602203651	1.74×10^{-11}
	Topological domain	29.83	0.04253202092	3.34×10^{-7}
	Intramembrane	30.16	0.04253202092	3.34×10^{-7}
Variants/mutagenesis	Alternative sequence	25.45	0.04932749288	1.19×10^{-5}
	Mutagenesis	21.64	0.04548693962	4.00×10^{-5}
	Natural variant	10.72	0.03201004823	6.28×10^{-3}
	Sequence conflict	6.07	0.02409030952	4.81×10^{-2}

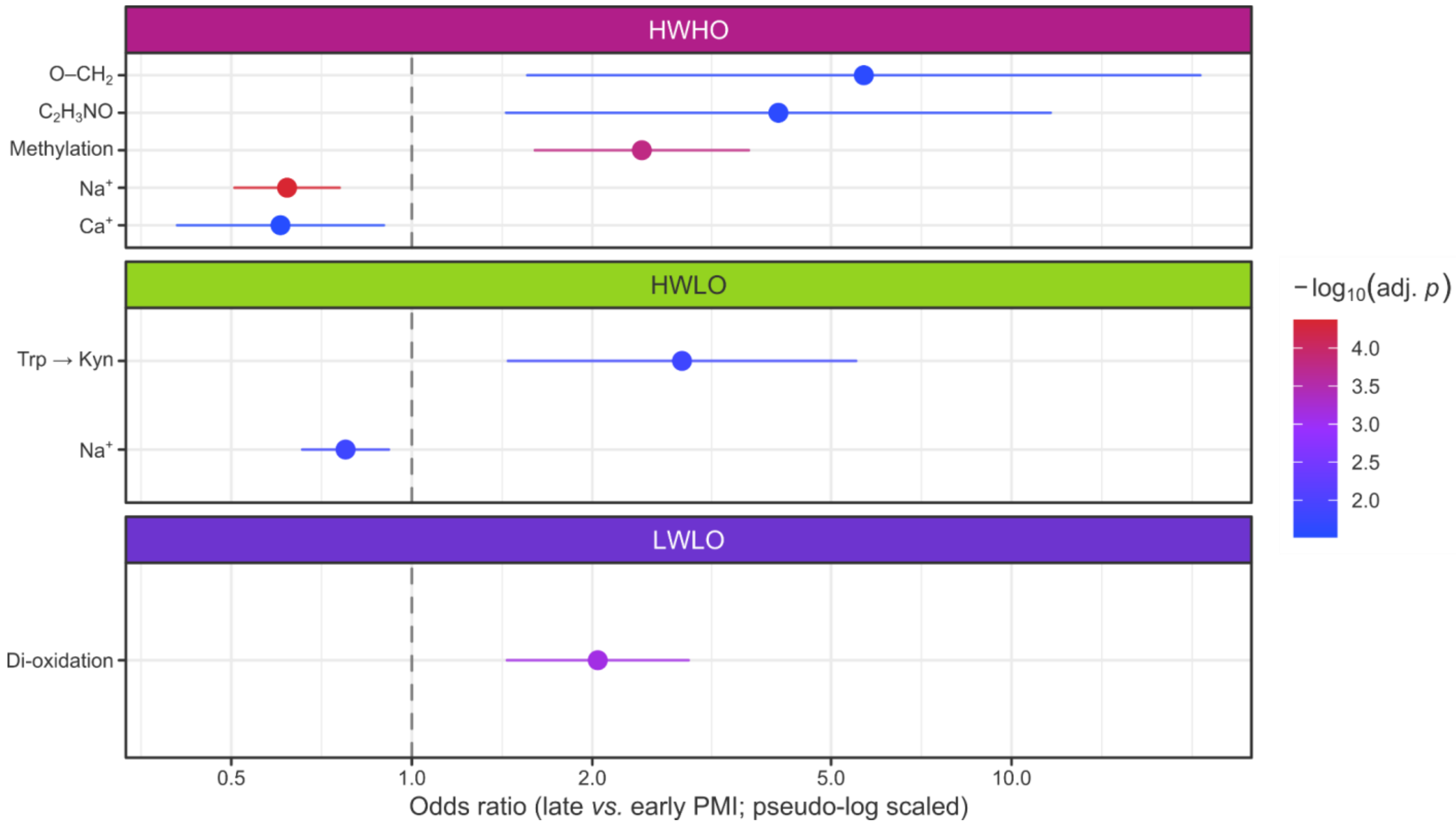

Fig S16. Odds of modification at late *vs.* early PMI by burial environment | Forest plots of ORs (pseudo-log scaled) of PTMs at late *vs.* early PMI within each experimental condition, estimated by mixed-effects logistic regression or, when convergence criteria were not met, by Firth's penalised logistic regression. Each point illustrates the direction and magnitude of the PMI effect, with horizontal lines representing 95% CIs and colour intensity corresponding to $-\log_{10}$ of adjusted *p*-value. The vertical dashed line marks OR = 1 (no effect), such that a point left of the dashed line indicates that PTM is more likely in the early PMI, and right of the dashed line in the late PMI. Only modifications with 95% CIs excluding 1 and $p < .05$ were considered statistically significant, and only significant results are shown. No PTMs in the dry–oxic condition met these criteria.

Table S22. Dominant radical oxidation pathways under contrasting hydration and oxygen regimes | The table presents mechanistic pathways inferred from the experimental results and established radical oxidation chemistry (Supplementary Discussion), summarising how water and oxygen availability jointly control radical initiation, chain propagation, and termination: water primarily governs molecular mobility and encounter probability, whereas oxygen controls chain length and the balance between propagation, regeneration, and termination. OH^- = hydroxide; •OH = hydroxyl radical; R–H = oxidisable protein site; R• = protein-centred radical; ROO• = peroxyl radical; ROOH = hydroperoxide; RO• = alkoxyl radical; R′• = new radical created after bond cleavage; R–R = crosslink; A–H = local electron or hydrogen donor (e.g., Trp, Tyr, Met); A• = corresponding donor-centred radical.

Condition	Initiation	Propagation	Termination	Net molecular consequence
**HWHO**	Primary radical generation (Fenton-type): $Fe^{2+} + H_2O_2 \rightarrow Fe^{3+} + OH^- +$ •OH<br>Secondary radical generation (H-abstraction): •OH + R–H → H_2O + R•	O_2 addition: R• + O_2 → ROO•<br>Chain propagation (peroxyl H-abstraction): ROO• + R–H → ROOH + R•<br>Radical regeneration (metal-mediated): ROOH + Fe^{2+} → RO• + OH^- + Fe^{3+}<br>Chain-amplifying fragmentation (β-scission): RO• → carbonyl-containing fragments + R′•	Late peroxyl termination after extended chain propagation: ROO• + ROO• → non-radical products (± O_2)<br>Crosslinking possible, but competes with ongoing propagation: R• + R• → R–R	Maximal chain-propagating oxidation with repeated radical regeneration and accumulation of ROOH/RO•, favouring backbone and side-chain cleavage, secondary radical formation (R′•), and extensive molecular loss.
**HWLO**	Primary radical generation (Fenton-type): $Fe^{2+} + H_2O_2 \rightarrow Fe^{3+} + OH^- +$ •OH<br>Secondary radical generation (H-abstraction): •OH + R–H → H_2O + R•	Water availability maintains molecular mobility and permits continued radical transfer and turnover, but reduced oxygen availability limits chain length and	Termination dominates at the site of radical generation, by:<br>Electron-transfer quenching: R• + A–H → R–H + A•<br>Crosslinking: R• + R• → R–	Repeated, spatially localised initiation–termination oxidation events with high probability of crosslinking per initiated radical, and preferential preservation *via*

		radical regeneration.	R	cumulative crosslinking.
**LWHO**	Initiation rate-limited by reduced molecular mobility. If initiation occurs:<br>Primary radical generation (Fenton-type): $Fe^{2+} + H_2O_2 \rightarrow Fe^{3+} + OH^- + \bullet OH$<br>Secondary radical generation (H-abstraction): $\bullet OH + R–H \rightarrow H_2O + R\bullet$	Propagation rate-limited by reduced molecular mobility.<br>O_2 addition occurs, but is encounter-limited. Chain propagation and radical regeneration are limited by restricted ion diffusion.	Termination dominates at the site of radical generation, most often by electron-transfer quenching: $R\bullet + A–H \rightarrow R–H + A\bullet$<br>Peroxyl termination possible, if generated locally: $ROO\bullet + ROO\bullet \rightarrow$ non-radical products ($\pm O_2$)<br>Crosslinking possible, but only if radicals generated in proximity: $R\bullet + R\bullet \rightarrow R–R$	Slow, heterogeneous oxidation dominated by mobility-limited kinetics, producing fewer long chains than HWHO and fewer crosslinks than HWLO. Net effect is partial oxidation rather than extensive fragmentation or stabilisation.
**LWLO**	Initiation most strongly rate-limited, with limited metal cycling, peroxide turnover, and substrate mobility. If initiation occurs:<br>Primary radical generation (H-abstraction): $\bullet OH + R–H \rightarrow H_2O + R\bullet$	Propagation rate-limited by reduced oxygen availability and reduced molecular mobility.<br>O_2 addition is suppressed. Chain propagation and radical regeneration are relatively rare and limited by restricted ion diffusion.	Termination dominates at the site of radical generation, most often by electron-transfer quenching: $R\bullet + A–H \rightarrow R–H + A\bullet$<br>Crosslinking possible, but only if radicals generated in proximity: $R\bullet + R\bullet \rightarrow R–R$	Minimal oxidation due to low radical flux. Chemical change is limited and spatially restricted; preservation arises primarily from suppression of oxidative chemistry rather than from stabilising crosslink formation.

Table S23. Reactivity and preservation potential of key amino acid residues | The table summarises how specific side-chain chemistries govern modification pathways: residues forming delocalised, stabilising radicals (e.g., Trp, Tyr, Met) promote crosslinking, whereas those yielding destructive or inert products (e.g., His, Gln, Ala, Ile, Leu) accelerate degradation.

**Amino acid**	**Key reactive group(s)**	**Typical modification pathway**	**Principical product(s)/effect(s)**	**Likely consequence for preservation**
Tryptophan (Trp)	Indole ring with weakly basic nitrogen; highly electron-rich π system.	One-electron oxidation *via* indolyl radical intermediate, followed by hydroxylation, oxidative ring cleavage, or radical coupling	Stable oxidised aromatics (e.g., Trp–Trp, Trp–Tyr, hydroxyindoles, kynurenines) capable of further radical coupling and intermolecular crosslinking.	Acts as a radical sink and nucleation site for polymerisation; promotes formation of insoluble aggregates; stabilising.
Tyrosine (Tyr)	Phenolic ring; moderately electron-rich π system.	Reversible one-electron oxidation *via* tyrosyl radical intermediate, followed by dimerisation.	Dityrosine and higher-order covalent crosslinks.	Produces covalently crosslinked networks resistant to hydrolysis and proteolysis; stabilising.
Methionine (Met)	Thioether sulfur; soft redox centre.	Two-electron oxidation to sulfoxide or sulfone; transient thiyl radicals possible.	Stable, non-fragmenting products that persist unchanged or slowly condense with reactive carbonyl species or neighbouring sulfur groups; reversible redox cycling.	Buffers oxidative flux; can mediate secondary crosslinking; stabilising.
Histidine (His)	Imidazole ring with two nitrogen donors; weakly electron-rich (amphoteric) π system.	Metal coordination leading to Fenton-type radical generation and self-oxidation.	2-oxo-His, ring-cleaved or fragmented products.	Generates local reactive oxygen species; self-oxidising and locally destabilising, but may promote system-level preservation *via* promotion of oxidative

				crosslinking.
Glutamine (Gln)	Terminal amide group.	Redox-inactive; hydrolytic deamidation or *via* cyclic imide intermediate.	Carboxylate and either Glu or iso-Glu plus ammonia; increases hydrophilicity.	Promotes peptide cleavage and solubilisation; destabilising.
Phenylalanine (Phe)	Benzyl ring lacking heteroatoms; electron-neutral π system.	Slow peroxidation at benzylic C–H *via* benzylic radical intermediates with limited radical stability.	Unstable (hydro)peroxides decompose to reactive carbonyl species, leading to side-chain or backbone cleavage.	Chemically inert toward crosslinking; prone to fragmentation; destabilising.
Aliphatic residues (Ala, Val, Ile)	Saturated C–H bonds.	Hydrogen abstraction and oxidation at tertiary carbons *via* peroxyl radical intermediates, followed by β-scission.	Reactive carbonyl species.	Promotes fragmentation; destabilising.
Small/flexible residues (Gly, Pro, Thr)	Minimal side chains; conformational freedom.	Limited redox chemistry; hydrolysis, dehydration, and condensation reactions enabling secondary crosslinking.	Confer flexibility or steric protection at or near termini; stabilise fragments and promote aggregation *via* hydrogen bonding or condensation.	Protects N-termini from cleavage; aids aggregation by bringing reactive groups into proximity; moderately stabilising.
Lysine (Lys)	Primary ε-amine.	Limited redox chemistry; Schiff-base formation and carbonyl condensation with oxidised lipids or sugars.	Crosslinks with reactive carbonyl species; Advanced glycoxidation/lipoxidation products, imines, and Michael adducts.	Participates in secondary condensation; moderately stabilising.
Acidic residues (Asp, Glu)	Carboxylate.	Redox-inactive; metal coordination, acid/base-catalysed hydrolysis.	Metal complexes or salt bridges; potential peptide cleavage.	Generally destabilising, but may confer local stability through ionic or metal-mediated interactions.

Table S24. Sample mass and protein concentration for the DDA dataset | Starting mass, volume of SDS lysis buffer, and protein concentration of samples (n = 72) *post*-lysis, estimated using BCA assay with BSA standards, followed by normalised volumes of sample and buffer for reduction, alkylation and digestion.

Time point	Sample ID	Sample mass (mg)	Buffer volume (µL)	Concentration (µg/µL)
T_1	HWHO_24hr_A	89.5	1600	4.05
	HWHO_24hr_B	89.2	1600	2.61
	HWHO_24hr_C	105.6	1600	3.53
	HWLO_24hr_A	124.5	1600	3.97
	HWLO_24hr_B	86.3	1553	0.66
	HWLO_24hr_C	102.3	1600	0.43

Time point	Sample ID	Sample mass (mg)	Buffer volume (µL)	Concentration (µg/µL)
	LWHO_24hr_A	63.9	1150	0.14
	LWHO_24hr_B	76.6	1379	3.09
	LWHO_24hr_C	87.1	1568	3.09
	LWLO_24hr_A	94.5	1600	3.43
	LWLO_24hr_B	75.1	1352	3.04
	LWLO_24hr_C	88.0	1584	3.69
T_2	HWHO_72hr_A	74.9	1348	3.63
	HWHO_72hr_B	119.3	1600	4.90
	HWHO_72hr_C	83.1	1496	2.91
	HWLO_72hr_A	96.3	1600	3.58
	HWLO_72hr_B	102.0	1600	3.16
	HWLO_72hr_C	118.9	1600	4.90
	LWHO_72hr_A	86.5	1557	2.34
	LWHO_72hr_B	95.2	1600	0.64
	LWHO_72hr_C	78.3	1409	3.97
	LWLO_72hr_A	84.5	1521	8.27
	LWLO_72hr_B	109.7	1600	3.52
	LWLO_72hr_C	92.8	1600	3.16
T_3	HWHO_1wk_A	100.5	1600	0.37
	HWHO_1wk_B	99.7	1600	7.39
	HWHO_1wk_C	104.3	1600	7.39
	HWLO_1wk_A	71.3	1283	2.58

Time point	Sample ID	Sample mass (mg)	Buffer volume (µL)	Concentration (µg/µL)
	HWLO_1wk_B	84.5	1521	2.72
	HWLO_1wk_C	101.2	1600	3.85
	LWHO_1wk_A	66.8	1202	2.61
	LWHO_1wk_B	78.6	1415	3.18
	LWHO_1wk_C	86.7	1561	3.71
	LWLO_1wk_A	77.6	1397	3.40
	LWLO_1wk_B	78.9	1420	3.30
	LWLO_1wk_C	85.8	1544	4.12
T_4	HWHO_6wk_A	80.6	1451	0.06
	HWHO_6wk_B	88.5	1593	0.14
	HWHO_6wk_C	39.9	718	0.35
	HWLO_6wk_A	98.4	1600	2.25
	HWLO_6wk_B	55.9	1006	2.14
	HWLO_6wk_C	60.1	1082	3.39
	LWHO_6wk_A	41.6	749	3.51
	LWHO_6wk_B	34.8	626	0.33
	LWHO_6wk_C	38.7	697	1.93
	LWLO_6wk_A	77.1	1388	2.44
	LWLO_6wk_B	49.8	896	1.27
	LWLO_6wk_C	95.3	1600	2.77
T_5	HWHO_3mo_A	39.6	713	1.13
	HWHO_3mo_B	51.5	927	1.49

Time point	Sample ID	Sample mass (mg)	Buffer volume (µL)	Concentration (µg/µL)
	HWHO_3mo_C	62.0	1116	0.28
	HWLO_3mo_A	62.0	1116	1.28
	HWLO_3mo_B	92.2	1600	2.31
	HWLO_3mo_C	62.3	1121	1.35
	LWHO_3mo_A	33.9	610	1.58
	LWHO_3mo_B	43.6	785	4.82
	LWHO_3mo_C	43.5	783	1.65
	LWLO_3mo_A	64.7	1165	1.43
	LWLO_3mo_B	59.9	1078	5.03
	LWLO_3mo_C	47.3	851	0.90
T_6	HWHO_6mo_A	32.2	580	3.11
	HWHO_6mo_B	43.7	787	2.17
	HWHO_6mo_C	33.6	605	4.41
	HWLO_6mo_A	85.2	1534	0.49
	HWLO_6mo_B	96.7	1600	1.23
	HWLO_6mo_C	81.4	1465	0.52
	LWHO_6mo_A	28.7	517	0.76
	LWHO_6mo_B	28.2	508	0.79
	LWHO_6mo_C	98.0	1600	0.38
	LWLO_6mo_A	40.6	731	1.69
	LWLO_6mo_B	30.0	540	0.15
	LWLO_6mo_C	30.3	545	0.33

Table S25. Sample mass and protein concentration for the DIA dataset | Starting mass, volume of RIPA lysis buffer, and protein concentration of samples (n = 72) *post*-lysis, estimated using BCA assay with BSA standards, followed by normalised volumes of sample and buffer for reduction, alkylation and digestion.

Time point	Sample ID	Sample mass (mg)	Buffer volume (µL)	Concentration (µg/µL)	Normalised vol. of sample (µL) for 10 µg protein	Normalised vol. of buffer (µL) for 15 µL total
T_1	HWHO_24hr_A	8	80	11.10	0.90	14.10
	HWHO_24hr_B	13	130	8.41	1.19	13.81
	HWHO_24hr_C	32	320	8.40	1.19	13.81
	HWLO_24hr_A	17	170	8.40	1.19	13.81
	HWLO_24hr_B	27	270	8.28	1.21	13.79
	HWLO_24hr_C	17	170	8.18	1.22	13.78
	LWHO_24hr_A	17	170	6.40	1.56	13.44
	LWHO_24hr_B	37	370	9.15	1.09	13.91
	LWHO_24hr_C	45	450	7.04	1.42	13.58
	LWLO_24hr_A	18	180	9.39	1.06	13.94
	LWLO_24hr_B	24	240	8.64	1.16	13.84
	LWLO_24hr_C	22	220	10.25	0.98	14.02
T_2	HWHO_72hr_A	20	200	10.69	0.94	14.06
	HWHO_72hr_B	22	220	8.05	1.24	13.76
	HWHO_72hr_C	37	370	8.41	1.19	13.81
	HWLO_72hr_A	13	130	8.37	1.19	13.81
	HWLO_72hr_B	47	470	7.74	1.29	13.71
	HWLO_72hr_C	39	390	8.88	1.13	13.87
	LWHO_72hr_A	56	560	8.75	1.14	13.86
	LWHO_72hr_B	34	340	8.83	1.13	13.87

Time point	Sample ID	Sample mass (mg)	Buffer volume (µL)	Concentration (µg/µL)	Normalised vol. of sample (µL) for 10 µg protein	Normalised vol. of buffer (µL) for 15 µL total
	LWHO_72hr_C	47	470	9.35	1.07	13.93
	LWLO_72hr_A	36	360	7.98	1.25	13.75
	LWLO_72hr_B	36	360	8.56	1.17	13.83
	LWLO_72hr_C	22	220	9.32	1.07	13.93
T_3	HWHO_1wk_A	15	150	7.36	1.36	13.64
	HWHO_1wk_B	48	480	8.38	1.19	13.81
	HWHO_1wk_C	9	90	7.22	1.39	13.61
	HWLO_1wk_A	40	400	8.03	1.25	13.75
	HWLO_1wk_B	50	500	7.31	1.37	13.63
	HWLO_1wk_C	33	330	7.73	1.29	13.71
	LWHO_1wk_A	18	180	8.04	1.24	13.76
	LWHO_1wk_B	26	260	6.88	1.45	13.55
	LWHO_1wk_C	42	420	7.70	1.30	13.70
	LWLO_1wk_A	59	590	7.68	1.30	13.70
	LWLO_1wk_B	26	260	6.34	1.58	13.42
	LWLO_1wk_C	46	460	10.05	1.00	14.00
T_4	HWHO_6wk_A	3	30	4.28	2.34	12.66
	HWHO_6wk_B	37	370	0.94	10.68	4.32
	HWHO_6wk_C	7	70	6.76	1.48	13.52
	HWLO_6wk_A	46	460	2.66	3.76	11.24
	HWLO_6wk_B	24	240	3.31	3.02	11.98

Time point	Sample ID	Sample mass (mg)	Buffer volume (µL)	Concentration (µg/µL)	Normalised vol. of sample (µL) for 10 µg protein	Normalised vol. of buffer (µL) for 15 µL total
	HWLO_6wk_C	29	290	3.43	2.91	12.09
	LWHO_6wk_A	16	160	6.29	1.59	13.41
	LWHO_6wk_B	9	90	8.21	1.22	13.78
	LWHO_6wk_C	6	60	6.32	1.58	13.42
	LWLO_6wk_A	20	200	4.86	2.06	12.94
	LWLO_6wk_B	20	200	7.44	1.34	13.66
	LWLO_6wk_C	15	150	3.94	2.54	12.46
T_5	HWHO_3mo_A	25	250	1.79	5.60	9.40
	HWHO_3mo_B	4	40	4.86	2.06	12.94
	HWHO_3mo_C	5	50	1.05	9.50	5.50
	HWLO_3mo_A	5	50	2.59	3.87	11.13
	HWLO_3mo_B	6	60	3.91	2.56	12.44
	HWLO_3mo_C	7	70	2.68	3.73	11.27
	LWHO_3mo_A	12	120	6.75	1.48	13.52
	LWHO_3mo_B	4	40	7.35	1.36	13.64
	LWHO_3mo_C	7	70	7.49	1.34	13.66
	LWLO_3mo_A	2	30	6.93	1.44	13.56
	LWLO_3mo_B	14	140	3.43	2.91	12.09
	LWLO_3mo_C	21	210	4.06	2.46	12.54
T_6	HWHO_6mo_A	1	30	0.64	15.0	0.00
	HWHO_6mo_B	1	30	0.06	15.0	0.00

Time point	Sample ID	Sample mass (mg)	Buffer volume (µL)	Concentration (µg/µL)	Normalised vol. of sample (µL) for 10 µg protein	Normalised vol. of buffer (µL) for 15 µL total
	HWHO_6mo_C	9	90	2.14	4.67	10.33
	HWLO_6mo_A	18	180	1.17	8.55	6.45
	HWLO_6mo_B	14	140	2.46	4.06	10.94
	HWLO_6mo_C	26	260	1.55	6.44	8.56
	LWHO_6mo_A	3	30	0.14	15.0	0.00
	LWHO_6mo_B	8	80	2.03	4.94	10.06
	LWHO_6mo_C	5	50	3.61	2.77	12.23
	LWLO_6mo_A	13	130	3.35	2.99	12.01
	LWLO_6mo_B	7	70	4.53	2.21	12.79
	LWLO_6mo_C	7	70	3.46	2.89	12.11

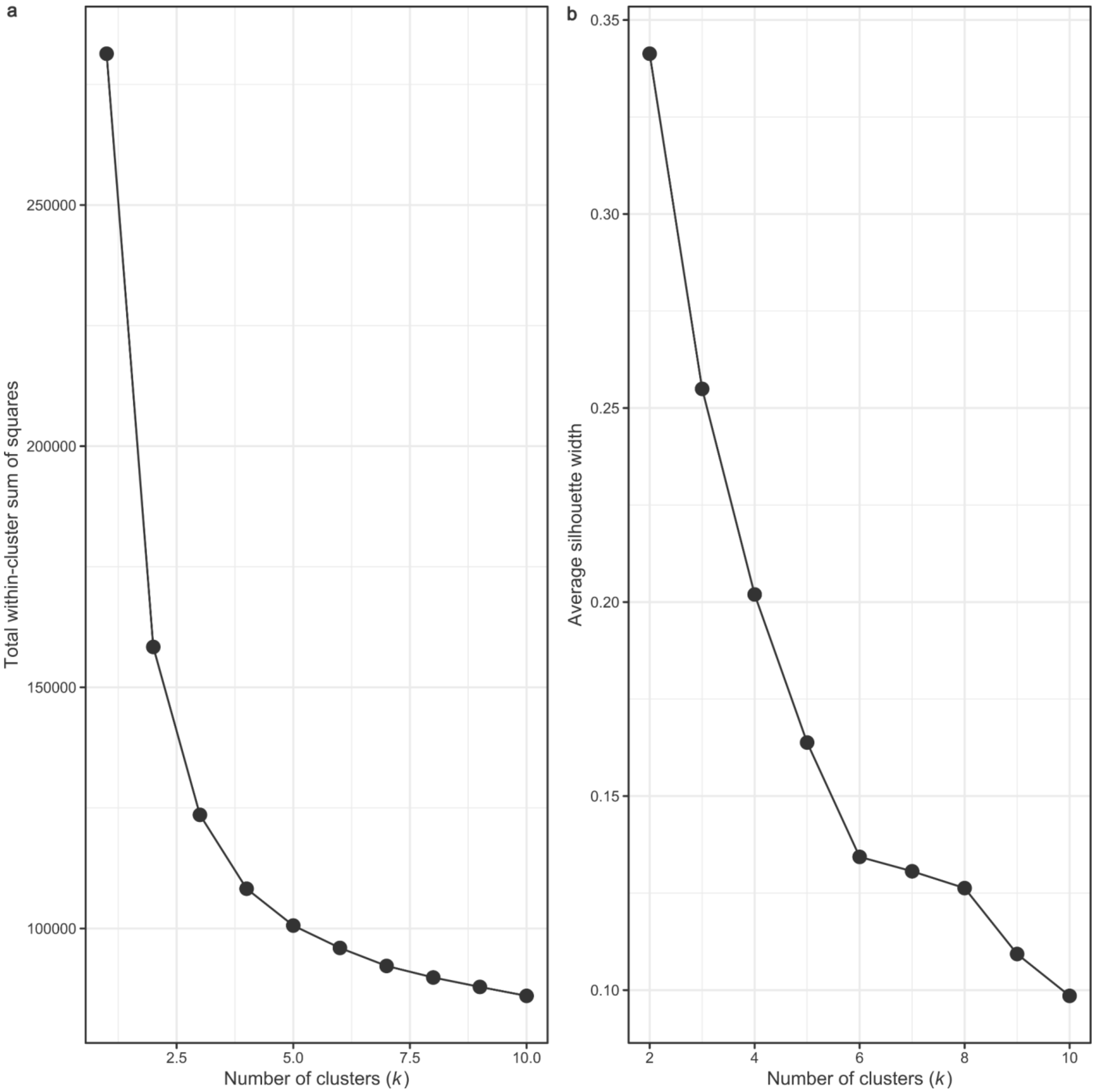

Fig S17. Determination of optimal number of clusters for hierarchical clustering analysis of iBAQ data | (a) Elbow method plot depicting total within-cluster sum of squares against the number of clusters (k). **(b)** Silhouette analysis illustrating the mean silhouette width against k.

References

1. Powers RH. The decomposition of human remains: a biochemical perspective. Forensic Medicine of the Lower Extremity. Totowa, NJ: Humana Press; 2005. pp. 3–15.
2. von Sonntag C, Schuchmann H-P. The elucidation of peroxyl radical reactions in aqueous solution with the help of radiation-chemical methods. Angew Chem, Int Ed Engl. 1991;30: 1229–1253.
3. Chatgilialoglu C, Barata-Vallejo S, Gimisis T. Radical reactions in organic synthesis: Exploring in-, on-, and with-water methods. Molecules. 2024;29: 569.
4. Migliore A, Corni S, Felice RD, Molinari E. Water-mediated electron transfer between protein redox centers. J Phys Chem B. 2007;111: 3774–3781.
5. Malferrari M, Francia F, Venturoli G. Retardation of protein dynamics by trehalose in dehydrated systems of photosynthetic reaction centers. Insights from electron transfer and thermal denaturation kinetics. J Phys Chem B. 2015;119: 13600–13618.
6. Muranov KO. Fenton Reaction in vivo and in vitro. Possibilities and Limitations. Biochemistry (Mosc). 2024;89: S112–S126.
7. Davies MJ. Protein oxidation and peroxidation. Biochem J. 2016;473: 805–825.
8. Pliss E, Pokidova T, Sirik A, Grobov A, Pitsyn N, Machtin V, et al. Kinetic analysis of the reactivity of peroxyl radicals in chain oxidation of unsaturated compounds. React Kinet Mech Catal. 2023;137: 53–76.
9. Figueroa JD, Zárate AM, Fuentes-Lemus E, Davies MJ, López-Alarcón C. Formation and characterization of crosslinks, including Tyr-Trp species, on one electron oxidation of free Tyr and Trp residues by carbonate radical anion. RSC Adv. 2020;10: 25786–25800.
10. Marschner P. Processes in submerged soils – linking redox potential, soil organic matter turnover and plants to nutrient cycling. Plant Soil. 2021;464: 1–12.
11. Schirò G, Weik M. Role of hydration water in the onset of protein structural dynamics. J Phys Condens Matter. 2019;31: 463002.
12. Zheng L, Dong H, Su G, Zhao Q, Zhao M. Radical scavenging activities of Tyr-, Trp-, Cys- and Met-Gly and their protective effects against AAPH-induced oxidative damage in human erythrocytes. Food Chem. 2016;197: 807–813.
13. Zheng L, Zhao Y, Dong H, Su G, Zhao M. Structure–activity relationship of antioxidant dipeptides: Dominant role of Tyr, Trp, Cys and Met residues. J Funct Foods. 2016;21: 485–496.
14. Fuentes-Lemus E, Hägglund P, López-Alarcón C, Davies MJ. Oxidative crosslinking of peptides and proteins: Mechanisms of formation, detection, characterization and quantification. Molecules. 2021;27: 15.
15. Ehrenshaft M, Deterding LJ, Mason RP. Tripping up Trp: Modification of protein tryptophan residues by reactive oxygen species, modes of detection, and biological consequences. Free Radic Biol Med. 2015;89: 220–228.
16. Bellmaine S, Schnellbaecher A, Zimmer A. Reactivity and degradation products of tryptophan in solution and proteins. Free Radic Biol Med. 2020;160: 696–718.
17. Folkes LK, Bartesaghi S, Trujillo M, Wardman P, Radi R. The effects of nitric oxide or oxygen on the stable products formed from the tyrosine phenoxyl radical. Free Radic Res. 2021;55: 141–153.
18. Gatin A, Billault I, Duchambon P, Van der Rest G, Sicard-Roselli C. Oxidative radicals (HO• or N3•) induce several di-tyrosine bridge isomers at the protein scale. Free Radic Biol Med. 2021;162: 461–470.
19. Mukherjee S, Kapp EA, Lothian A, Roberts AM, Vasil'ev YV, Boughton BA, et al. Characterization and identification of dityrosine cross-linked peptides using tandem mass spectrometry. Anal Chem. 2017;89: 6136–6145.
20. Souza JM, Giasson BI, Chen Q, Lee VM, Ischiropoulos H. Dityrosine cross-linking promotes formation of stable alpha -synuclein polymers. Implication of nitrative and oxidative stress in the pathogenesis of neurodegenerative synucleinopathies. J Biol Chem. 2000;275: 18344–18349.
21. Maina MB, Al-Hilaly YK, Serpell LC. Dityrosine cross-linking and its potential roles in Alzheimer's disease. Front Neurosci. 2023;17: 1132670.
22. Drazic A, Winter J. The physiological role of reversible methionine oxidation. Biochim Biophys Acta. 2014;1844: 1367–1382.
23. Levine RL, Moskovitz J, Stadtman ER. Oxidation of methionine in proteins: roles in antioxidant defense and cellular regulation. IUBMB Life. 2000;50: 301–307.
24. Kim G, Weiss SJ, Levine RL. Methionine oxidation and reduction in proteins. Biochim Biophys Acta. 2014;1840: 901–905.
25. Schöneich C. Thiyl radicals and induction of protein degradation. Free Radic Res. 2016;50: 143–149.
26. Weber DS, Warren JJ. A survey of methionine-aromatic interaction geometries in the oxidoreductase class of enzymes: What could Met-aromatic interactions be doing near metal sites? J Inorg Biochem. 2018;186: 34–41.
27. Orabi EA, Esam AM. Predicting structural and energetic changes in Met-aromatic motifs on methionine

oxidation to the sulfoxide and sulfone. Phys Chem Chem Phys. 2018;20: 23132–23141.
28. Wang C, Wang Z, Zeng B, Zheng M, Xiao N, Zhao Z. Fenton-like reaction of the iron(II)-histidine complex generates hydroxyl radicals: implications for oxidative stress and Alzheimer's disease. Chem Commun (Camb). 2021;57: 12293–12296.
29. Liu Y, Sun G, David A, Sayre LM. Model studies on the metal-catalyzed protein oxidation: structure of a possible His-Lys cross-link. Chem Res Toxicol. 2004;17: 110–118.
30. Li X, Lin C, O'Connor PB. Glutamine deamidation: differentiation of glutamic acid and gamma-glutamic acid in peptides by electron capture dissociation. Anal Chem. 2010;82: 3606–3615.
31. Uyaver S. Tyrosine, phenylalanine, and tryptophan undergo self-aggregation in similar and different manners. Atmosphere (Basel). 2022;13: 1448.
32. Saladino J, Liu M, Live D, Sharp JS. Aliphatic peptidyl hydroperoxides as a source of secondary oxidation in hydroxyl radical protein footprinting. J Am Soc Mass Spectrom. 2009;20: 1123–1126.
33. Gebicki S, Gebicki JM. Formation of peroxides in amino acids and proteins exposed to oxygen free radicals. Biochemical Journal. 1993;289: 743–749.
34. Kløve S, Graversen KB, Rasmussen JA, Assis J, Schluter J, Blaser MJ, et al. Early-life Helicobacter pylori infection worsens metabolic state in mice receiving a high-fat diet. Evolutionary Biology. bioRxiv; 2025. Available: https://www.biorxiv.org/content/10.1101/2025.04.27.650497v1.full.pdf
35. Hughes CS, Moggridge S, Müller T, Sorensen PH, Morin GB, Krijgsveld J. Single-pot, solid-phase-enhanced sample preparation for proteomics experiments. Nat Protoc. 2019;14: 68–85.
36. Perez-Riverol Y, Bandla C, Kundu DJ, Kamatchinathan S, Bai J, Hewapathirana S, et al. The PRIDE database at 20 years: 2025 update. Nucleic Acids Res. 2025;53: D543–D553.
37. Korovesi AG, Anagnostopoulos AK, Pierros V, Stravopodis DJ, Tsangaris GT. Normal Mouse Brain Proteome II: Analysis of Brain Regions by High-resolution Mass Spectrometry. Cancer Genomics Proteomics. 2020;17: 757–767.
38. Tyanova S, Temu T, Cox J. The MaxQuant computational platform for mass spectrometry-based shotgun proteomics. Nat Protoc. 2016;11: 2301–2319.
39. Hildonen S, Halvorsen TG, Reubsaet L. Why less is more when generating tryptic peptides in bottom-up proteomics. Proteomics. 2014;14: 2031–2041.
40. Demichev V, Messner CB, Vernardis SI, Lilley KS, Ralser M. DIA-NN: neural networks and interference correction enable deep proteome coverage in high throughput. Nat Methods. 2020;17: 41–44.
41. Gerault M-A, Camoin L, Granjeaud S. DIAgui: a Shiny application to process the output from DIA-NN. Bioinform Adv. 2024;4: vbae001.
42. Pham TV, Henneman AA, Jimenez CR. iq: an R package to estimate relative protein abundances from ion quantification in DIA-MS-based proteomics. Bioinformatics. 2020;36: 2611–2613.
43. Cox J, Hein MY, Luber CA, Paron I, Nagaraj N, Mann M. Accurate proteome-wide label-free quantification by delayed normalization and maximal peptide ratio extraction, termed MaxLFQ. Mol Cell Proteomics. 2014;13: 2513–2526.
44. Lazar C, Gatto L, Ferro M, Bruley C, Burger T. Accounting for the multiple natures of missing values in label-free quantitative proteomics data sets to compare imputation strategies. J Proteome Res. 2016;15: 1116–1125.
45. Liu M, Dongre A. Proper imputation of missing values in proteomics datasets for differential expression analysis. Brief Bioinform. 2021;22.
46. Schwanhäusser B, Busse D, Li N, Dittmar G, Schuchhardt J, Wolf J, et al. Global quantification of mammalian gene expression control. Nature. 2011;473: 337–342.
47. Leek JT, Johnson WE, Parker HS, Jaffe AE, Storey JD. The sva package for removing batch effects and other unwanted variation in high-throughput experiments. Bioinformatics. 2012;28: 882–883.
48. Kassambara A MF. *factoextra: Extract and Visualize the Results of Multivariate Data Analyses*. 2020. Available: https://github.com/kassambara/factoextra
49. McInnes L, Healy J, Saul N, Großberger L. UMAP: Uniform Manifold Approximation and Projection. J Open Source Softw. 2018;3: 861.
50. Ritchie ME, Phipson B, Wu D, Hu Y, Law CW, Shi W, et al. limma powers differential expression analyses for RNA-sequencing and microarray studies. Nucleic Acids Res. 2015;43: e47.
51. Yu G, Wang L-G, Han Y, He Q-Y. clusterProfiler: an R package for comparing biological themes among gene clusters. OMICS. 2012;16: 284–287.
52. Yu G. *enrichplot: Visualization of Functional Enrichment Result*. 2025. Available: https://bioconductor.org/packages/enrichplot
53. Gu Z. Complex heatmap visualization. Imeta. 2022;1: e43.
54. Wood S. mgcv: Mixed GAM Computation Vehicle with Automatic Smoothness Estimation. 2025.

55. Thomas PD, Ebert D, Muruganujan A, Mushayahama T, Albou L-P, Mi H. PANTHER: Making genome-scale phylogenetics accessible to all. Protein Sci. 2022;31: 8–22.
56. Kyte J, Doolittle RF. A simple method for displaying the hydropathic character of a protein. J Mol Biol. 1982;157: 105–132.
57. Cohen J. Statistical Power Analysis for the Behavioral Sciences. Mahwah, NJ: Lawrence Erlbaum Associates; 1988.
58. Osorio D, Rondón-Villarreal P, Torres R. Peptides: A package for data mining of antimicrobial peptides. R J. 2015;7: 4.
59. Kozlowski LP. IPC 2.0: prediction of isoelectric point and pKa dissociation constants. Nucleic Acids Res. 2021;49: W285–W292.
60. Cliff N. Dominance statistics: Ordinal analyses to answer ordinal questions. Psychol Bull. 1993;114: 494–509.
61. Jumper J, Evans R, Pritzel A, Green T, Figurnov M, Ronneberger O, et al. Highly accurate protein structure prediction with AlphaFold. Nature. 2021;596: 583–589.
62. Grant BJ, Rodrigues APC, ElSawy KM, McCammon JA, Caves LSD. Bio3d: an R package for the comparative analysis of protein structures. Bioinformatics. 2006;22: 2695–2696.
63. Erdős G, Dosztányi Z. AIUPred: combining energy estimation with deep learning for the enhanced prediction of protein disorder. Nucleic Acids Res. 2024;52: W176–W181.
64. Ahmad S, Jose da Costa Gonzales L, Bowler-Barnett EH, Rice DL, Kim M, Wijerathne S, et al. The UniProt website API: facilitating programmatic access to protein knowledge. Nucleic Acids Res. 2025;53: W547–W553.
65. Heinze G, Ploner M, Jiricka L, Steiner G. logistf: Firth's Bias-Reduced Logistic Regression. 2025. Available: https://github.com/georgheinze/logistf
